# Supplementary material for: Multi-Tissue Metabolomic Signatures of Five Longevity Interventions Converge on Ergothioneine and Lipid Remodeling in Male UM-HET3 Mice
Source: bioRxiv. 2026 Jul 2:2026.06.24.734388. Preprint. [Version 2] doi: 10.64898/2026.06.24.734388 (PMC13345011; doi:10.64898/2026.06.24.734388)
Supplement: Supplement 1 [file media-1.pdf]

Feature	Plasma Rank	Brain Rank	GFat Rank	IFat Rank	Liver Rank	Muscle Rank	Kidney Rank
TG 48:3 | TG 14:0_16:1_18:2	1	ND	ND	606	ND	ND	ND
TG O-53:9 | TG O-19:5_17:2_17:2	2	ND	ND	ND	ND	ND	ND
TG 56:9 | TG 16:1_18:2_22:6	3	ND	ND	648	ND	ND	ND
N-Methylisoleucine	4	ND	ND	ND	ND	ND	ND
TG 50:3 | TG 16:0_16:1_18:2	5	ND	ND	ND	ND	ND	ND
TG 64:16 | TG 20:4_22:6_22:6	6	ND	ND	ND	ND	ND	ND
Ergothioneine	7	1	ND	377	217	13	940
TG 55:7 | TG 15:0_18:1_22:6	8	ND	ND	ND	ND	ND	ND
TG 62:9 | TG 22:1_18:2_22:6	9	ND	ND	ND	ND	ND	ND
5-Oxo-1-propyl-2-pyrrolidineacetic acid	10	ND	ND	ND	ND	ND	ND
TG 58:5 | TG 22:1_18:2_18:2	11	ND	ND	670	ND	ND	ND
PC 35:4	12	318	ND	ND	528	24	552
TG 60:10 b	13	ND	ND	ND	ND	ND	ND
TG 53:3 | TG 17:0_17:1_19:2	14	ND	ND	ND	ND	ND	ND
N-Acetylaspartylglutamic acid	15	243	ND	362	ND	ND	ND
Taurocholic acid	16	ND	ND	ND	248	944	801
TG 64:17 | TG 20:5_22:6_22:6	17	ND	ND	ND	ND	ND	ND
N-omega-Acetylhistamine	18	ND	ND	345	11	ND	51
DG 38:5	19	496	53	ND	34	794	337
N-Acetyltyrosine	20	ND	ND	ND	ND	ND	ND
TG 66:18 | TG 22:6_22:6_22:6	21	ND	ND	ND	ND	ND	ND
PE 36:2 | PE 18:0_18:2	22	ND	ND	465	ND	ND	ND
SM 36:1;2O	23	ND	ND	ND	ND	ND	ND
3-Hydroxybutyric acid	24	ND	ND	ND	ND	ND	ND
TG 60:13 | TG 18:2_20:5_22:6	25	ND	ND	ND	ND	ND	ND
TG 62:14 | TG 18:2_22:6_22:6	26	ND	ND	698	ND	896	235
DG 36:2 | DG 18:1_18:1	27	ND	ND	37	ND	ND	ND
TG 57:9 | TG 17:1_18:2_22:6	28	ND	ND	661	ND	ND	ND
TG 64:14 | TG 18:2_22:6_24:6	29	ND	ND	ND	ND	ND	ND
TG 51:2 | TG 16:0_17:1_18:1	30	ND	ND	273	ND	ND	ND
PC O-40:8	31	ND	ND	ND	ND	552	ND
2[4-(Trifluoromethoxy)phenyl]cyclopropanecarboxylic acid	32	ND	ND	ND	ND	ND	ND
Sulfamethoxazole	33	ND	ND	ND	ND	ND	ND
3-Hydroxyvaleric acid	34	ND	ND	ND	ND	ND	ND
PI 36:1 | PI 18:0_18:1_a	35	ND	ND	ND	ND	ND	ND
TG 58:8 | TG 18:1_18:2_22:5	36	ND	ND	ND	ND	ND	ND
DG 38:7 | DG 18:2_20:5	37	ND	ND	ND	ND	795	ND
DG 40:8 | DG 18:2_22:6	38	ND	ND	434	ND	801	ND
3-Pyridinemthanol	39	ND	ND	ND	ND	ND	ND
DG 36:2	40	365	40	ND	541	784	667
PC 42:10 | PC 21:5_21:5	41	ND	ND	ND	ND	ND	ND
N-Methyllysine	42	32	ND	ND	ND	ND	208

3-(3-Hydroxyphenyl)propionic acid	43	ND	ND	ND	ND	ND	ND
DG 40:8	44	ND	ND	ND	ND	ND	289
1,5-Anhydro-D-sorbitol	45	ND	ND	ND	ND	ND	ND
1-(Piperidin-4-yl)ethan-1-ol	46	ND	ND	ND	ND	ND	32
PC 42:7	47	ND	ND	ND	ND	36	15
CAR 18:0	48	ND	ND	ND	ND	614	ND
PE P-40:6 | PE P-18:0_22:6	49	ND	ND	ND	ND	643	ND
TG 61:13 | TG 18:2_21:5_22:6	50	ND	ND	ND	ND	ND	ND
SM 42:1;2O	51	ND	ND	ND	ND	ND	ND
LPC 16:1/0:0	52	ND	ND	ND	ND	ND	ND
Cer 40:1;2O | Cer 18:1;2O/22:0	53	ND	ND	ND	ND	ND	ND
SE 29:1/18:2	54	ND	ND	ND	ND	ND	ND
5-alpha-Androstan-3-beta-ol-17-one sulfate	55	ND	ND	ND	ND	ND	ND
O2_PE 36:1	56	ND	ND	ND	ND	ND	ND
N-alpha-Acetyl-arginine	57	307	ND	ND	ND	ND	ND
10.87_603.53	58	ND	ND	ND	ND	ND	ND
Deoxycholic acid	59	ND	ND	ND	ND	ND	ND
TG 60:11 | TG 18:2_20:3_22:6	60	ND	ND	ND	ND	ND	ND
4-Imidazoleacetic acid	61	ND	233	510	ND	ND	ND
PC 40:5	62	477	ND	439	517	508	554
PC 37:5 | PC 17:0_20:5	63	ND	ND	ND	ND	ND	ND
1-Pentadecanoyl-sn-glycero-3-phosphocholine	64	ND	ND	ND	ND	ND	ND
gamma-Glutamyltyrosine	65	ND	ND	ND	ND	ND	ND
PC 36:4 | PC 16:0_20:4	66	ND	ND	ND	ND	ND	ND
N-acetylornithine	67	ND	ND	ND	ND	1078	ND
1-Heptadecanoyl-sn-glycero-3-phosphocholine	68	ND	ND	ND	ND	92	24
LPC 22:1/0:0	69	ND	ND	ND	ND	ND	ND
Naringenin-4'-O-.beta.-D-glucuronide	70	ND	ND	ND	ND	ND	ND
TG O-58:6 | TG O-20:0_16:0_22:6	71	ND	ND	ND	ND	ND	ND
TG 60:8 | TG 20:0_18:2_22:6	72	ND	ND	ND	ND	ND	ND
DG 38:7	73	ND	ND	ND	ND	ND	ND
PC 38:7	74	146	ND	ND	513	498	561
Glutamine	75	457	382	317	461	ND	311
(3-Oxo-2,3-dihydro-4H-1,4-benzoxazin-4-yl)acetic acid	76	ND	ND	ND	ND	ND	ND
(N-(-2-Acetamido))-2-aminoethanesulfonic acid	77	ND	ND	ND	ND	ND	ND
SM 41:1;2O	78	ND	ND	ND	ND	ND	ND
(3E)-4-(1-Hydroxy-2,2,6-trimethyl-4-oxocyclohexyl)but-3-en-2-yl hexopyranoside	79	ND	ND	ND	ND	ND	ND
TG 64:13 | TG 18:1_22:6_24:6	80	ND	ND	ND	ND	ND	ND
3-Methylglutarylcarnitine	81	ND	ND	ND	ND	ND	224
Lactitol	82	486	ND	ND	ND	ND	ND
Homocitrulline	83	ND	ND	ND	ND	ND	ND
PI 36:2	84	ND	ND	ND	ND	668	ND
Nicotinamide	85	560	267	356	ND	420	ND

TG 60:10 | TG 18:0_20:4_22:6	86	ND	ND	ND	ND	ND	ND
12R-Hydroxy-5Z,8Z,10E,14Z-eicosatetraenoic acid	87	ND	ND	ND	ND	ND	ND
2-Hydroxypalmitic acid	88	ND	ND	ND	ND	ND	ND
2,6-Dihydroxybenzoic acid	89	ND	ND	ND	ND	ND	ND
PI 36:4 | PI 16:0_20:4_a	90	ND	ND	ND	ND	ND	ND
FA 24:5	91	ND	ND	354	ND	57	ND
PC O-32:0	92	ND	ND	ND	ND	525	ND
Prolylalanine	93	ND	ND	ND	ND	ND	ND
gamma-Glutamylleucine	94	ND	ND	ND	ND	ND	876
Choline	95	376	354	707	309	766	ND
SM 36:1;2O | SM 18:1;2O/18:0	96	ND	ND	ND	ND	ND	ND
Ala-Thr	97	ND	ND	ND	ND	ND	310
TG 48:0 | TG 16:0_16:0_16:0	98	ND	ND	ND	ND	ND	ND
Gly-Pro	99	ND	378	ND	ND	ND	327
Taurodeoxycholic acid	100	ND	ND	ND	ND	ND	ND
1,4-Cyclohexanedicarboxylic acid	101	ND	ND	ND	ND	ND	ND
SM 36:2;2O	102	ND	ND	ND	ND	ND	ND
PC 37:6 | PC 15:0_22:6	103	ND	ND	ND	ND	274	ND
3-Hydroxy-3-methylglutaric acid_3-Hydroxy-3-methylglutaric acid	104	ND	ND	ND	ND	ND	ND
Suberic acid	105	ND	ND	ND	ND	ND	ND
1-O-Hexadecyl-2-O-acetyl-sn-glyceryl-3-phosphorylcholine	106	ND	ND	ND	ND	ND	ND
2-Hydroxy-4-methylpentanoic acid	107	ND	ND	ND	ND	ND	ND
gamma-Glutamylglutamine	108	ND	ND	ND	ND	ND	ND
PE P-42:6 | PE P-20:0_22:6	109	ND	ND	ND	ND	382	ND
(2R)-7-Methoxy-3-oxo-3,4-dihydro-2H-1,4-benzoxazin-2-yl .beta.-D-glucopyranoside	110	ND	ND	ND	ND	ND	ND
2-Aminonicotinic acid	111	ND	222	ND	ND	ND	ND
N-Acetylglycine	112	ND	ND	ND	ND	ND	ND
SM 33:1;2O | SM 17:1;2O/16:0	113	ND	ND	ND	ND	ND	ND
2-Thiobarbituric acid	114	ND	ND	ND	ND	ND	ND
(4-Methylphenyl)oxidanesulfonic acid	115	ND	ND	ND	ND	ND	ND
(3-Carboxypropyl)trimethylammonium	116	ND	37	35	ND	ND	ND
Methacholine	117	114	329	88	ND	1063	ND
FA 19:0	118	ND	ND	396	ND	ND	ND
Asn-Val	119	ND	ND	ND	ND	ND	ND
1-Stearoyl-2-hydroxy-sn-glycero-3-phosphoethanolamine	120	ND	ND	ND	ND	ND	ND
3-Cyclopentene-1-octanoic acid, 2-(3-hydroxy-1-penten-1-yl)-5-oxo-	121	ND	ND	ND	ND	ND	ND
2-Dec-9-enylpentanedioic acid	122	ND	ND	ND	ND	ND	ND
1-Myristoyl-sn-glycero-3-phosphocholine	123	ND	ND	ND	ND	ND	ND
1-Arachidoyl-2-hydroxy-sn-glycero-3-phosphocholine	124	ND	ND	ND	ND	ND	ND
1-Methylpiperidine-3-carboxylic acid	125	ND	ND	ND	ND	87	ND
DG 36:4 | DG 18:2_18:2	126	ND	ND	161	ND	ND	ND
O2_FA 22:1; (erucic acid) [M-H]-	127	ND	ND	ND	ND	ND	ND
PC 38:3 | PC 18:0_20:3	128	ND	ND	ND	ND	ND	ND

(2E)-2-(Propan-2-yl)but-2-enedioic acid	129	ND	ND	ND	ND	ND	ND
TG 60:14 | TG 18:3_20:5_22:6	130	ND	ND	ND	ND	ND	ND
Galactonic acid	131	ND	ND	ND	ND	ND	ND
(9E,11Z)-8-Hydroxyoctadeca-9,11-dienoic acid	132	ND	ND	ND	ND	ND	ND
TG O-60:6 | TG O-20:0_18:0_22:6	133	ND	ND	ND	ND	ND	ND
Actrarit	134	ND	ND	ND	ND	ND	ND
Ser-His	135	543	ND	ND	64	ND	686
FA 22:6;O	136	ND	ND	ND	ND	ND	ND
Phosphoric acid	137	ND	ND	ND	ND	ND	ND
LPC 19:0/0:0	138	ND	ND	ND	ND	ND	ND
(3-Oxo-2-piperazinyl)acetic acid	139	ND	ND	ND	ND	ND	ND
beta-Hydroxyisovaleric acid	140	ND	ND	ND	ND	ND	ND
HexCer 42:2;2O | HexCer 18:1;2O/24:1	141	ND	ND	294	ND	971	ND
(R)-Butyrylcarnitine	142	ND	162	ND	36	ND	35
2-((2R)-2-Hydroxycyclohexyl)acetic acid	143	ND	ND	ND	ND	ND	ND
3-Oxo-1,8-octanedicarboxylic acid	144	ND	ND	ND	ND	ND	ND
Acetyl-threonine	145	ND	ND	ND	ND	ND	ND
3-Hydroxybutyrylcarnitine	146	250	188	ND	ND	186	219
4,6-Dihydroxypyrimidine	147	ND	ND	ND	ND	ND	ND
Ala-Gln	148	ND	ND	ND	130	208	849
1-Methyladenosine	149	176	ND	ND	50	110	53
SM 32:1;2O | SM 16:1;2O/16:0	150	ND	ND	ND	ND	ND	ND
1-Hexadecyl-sn-glycero-3-phosphocholine	151	ND	ND	ND	ND	ND	57
1-Oleoyl-sn-glycero-3-phosphocholine	152	ND	ND	ND	ND	ND	ND
Methyl-beta-galactopyranoside	153	ND	ND	ND	ND	ND	ND
1-Hexadecanoyl-2-octadecadienoyl-sn-glycero-3-phosphocholine	154	ND	ND	ND	ND	ND	ND
(2R)-3-Hydroxyisovaleroylcarnitine	155	ND	129	ND	41	ND	27
5-Aminosalicylic acid	156	ND	ND	ND	ND	ND	ND
(1,5-Dimethyl-1H-pyrazol-3-yl)methylamine	157	ND	82	23	ND	ND	ND
Cer 42:1;2O | Cer 18:1;2O/24:0	158	ND	ND	ND	ND	ND	281
(2-oxo-2,3-dihydro-1H-indol-3-yl)acetic acid	159	ND	ND	ND	ND	ND	ND
4-Hydroxybenzaldehyde	160	ND	ND	ND	ND	ND	ND
2-Propanamidoacetic acid	161	ND	ND	ND	ND	ND	ND
11-Hydroxyundecanoic acid	162	ND	ND	ND	ND	ND	ND
1-(2-Hydroxyethyl)-2,2,6,6-tetramethyl-4-piperidinol	163	ND	140	ND	ND	ND	ND
CE 16:1	164	ND	ND	ND	ND	ND	ND
PC 41:6	165	ND	ND	ND	ND	ND	573
DG 38:3	166	ND	ND	ND	ND	ND	ND
LPC 14:0/0:0	167	ND	ND	ND	ND	ND	ND
Cholesterol 3-sulfate	168	ND	ND	ND	ND	ND	ND
(S)-2-Ureidopentanedioic acid	169	ND	ND	ND	ND	ND	ND
1-Palmitoyl-2-docosahexaenoyl-sn-glycero-3-phosphocholine	170	ND	ND	ND	ND	ND	72
3[(2-Methylbenzyl)sulfanyl]-1H-1,2,4-triazol-5-ylamine	171	ND	ND	ND	ND	ND	ND

PC 33:2	172	2	ND	ND	551	468	522
FA 15:0	173	ND	ND	ND	537	ND	68
TG 62:13|TG 18:1_22:6_22:6	174	ND	ND	696	ND	895	ND
PE P-36:4|PE P-16:0_20:4	175	ND	ND	ND	ND	ND	ND
DG 36:5	176	ND	152	29	542	789	ND
Ile-Leu	177	ND	ND	ND	ND	ND	375
1-Stearoyl-2-arachidonyl-sn-glycero-3-phosphocholine	178	ND	ND	ND	ND	ND	ND
Hexuronic acid	179	ND	ND	ND	ND	ND	ND
1,2-Diamino-2-methylpropane_1,2-Diamino-2-methylpropane	180	ND	ND	ND	ND	ND	ND
2,6-Diaminopimelic acid	181	ND	ND	ND	ND	ND	71
Pentaethylene glycol	182	ND	ND	ND	ND	ND	ND
PC 35:5	183	ND	ND	ND	ND	ND	ND
1-Methylnicotinamide	184	155	100	195	ND	95	54
Equol	185	ND	ND	ND	227	ND	ND
FA 24:0	186	165	ND	ND	27	ND	898
1-Oleoyl-sn-glycero-3-phosphoethanolamine	187	ND	ND	ND	ND	179	48
2-Amino-3-(dimethylamino)propionic acid	188	ND	ND	ND	ND	ND	ND
3-Aminopyridine	189	ND	175	68	ND	56	ND
4-(1,3-Benzothiazol-2-yl)butanoic acid	190	ND	ND	ND	ND	ND	ND
ST 28:1;O;S	191	ND	ND	ND	ND	ND	ND
1-Palmitoyl-sn-glycero-3-phosphocholine	192	ND	ND	ND	ND	ND	ND
2-Hydroxyphenylacetic acid	193	ND	ND	ND	ND	ND	ND
PI 40:5|PI 18:0_22:5	194	ND	ND	494	ND	685	ND
FA 25:0_a	195	ND	ND	ND	ND	ND	ND
2-Aminoadipic acid	196	ND	ND	ND	ND	ND	ND
TG 58:10|TG 18:2_18:2_22:6	197	ND	ND	663	ND	ND	ND
10-Hydroxydecanoic acid	198	ND	ND	ND	ND	ND	ND
(+)-Muscarine	199	ND	ND	ND	ND	ND	ND
Phenylpyruvic acid	200	ND	ND	ND	ND	ND	ND
PC 34:2;O|PC 16:0_18:2;O_a	201	ND	ND	ND	ND	ND	ND
Sarcosine	202	ND	ND	ND	ND	756	ND
PC O-42:7	203	ND	ND	ND	ND	ND	ND
3-Indoleacetic acid	204	ND	ND	ND	ND	ND	ND
1,4-Dihydroxy-2-naphthoic acid	205	ND	ND	ND	ND	ND	ND
PE O-38:6|PE O-18:2_20:4	206	ND	ND	496	ND	ND	ND
2-Methylglutamic acid	207	ND	ND	ND	ND	255	ND
2-(3-Methylbenzyl)butanedioic acid	208	ND	ND	ND	ND	ND	ND
2-Hydroxybenzaldehyde	209	ND	ND	ND	ND	ND	ND
1-Palmitoyl-2-hydroxy-sn-glycero-3-phosphoethanolamine	210	ND	ND	ND	ND	236	ND
Lactobionic acid	211	ND	ND	ND	ND	ND	ND
Gulono-1,4-lactone	212	ND	ND	ND	ND	ND	ND
2-Aminocaprylic acid	213	ND	ND	ND	ND	ND	ND
Erythronolactone	214	ND	ND	ND	ND	ND	ND

1-Hexadecylpyridinium	215	ND	ND	102	ND	ND	ND
1-Deoxynojirimycin	216	ND	ND	ND	ND	ND	ND
2,3-Dihydroxy-2-methylbutanoic acid	217	ND	ND	ND	ND	ND	ND
Butyrylcholine	218	ND	ND	ND	ND	ND	ND
3-Hydroxy-2-((9Z,12Z)-octadeca-9,12-dienoyloxy)propyl 2-(trimethylazaniumyl)ethyl phosphate	219	ND	ND	ND	ND	ND	8
Xanthurenic acid	220	ND	ND	ND	ND	ND	ND
Turanose	221	ND	ND	ND	ND	ND	ND
1-Methylguanine	222	ND	ND	ND	ND	ND	ND
2,2-Dimethyl-2,3-dihydro-4H-1,3-benzoxazin-4-one	223	ND	ND	ND	ND	ND	ND
Carnitine	224	474	331	565	133	231	600
2-Phosphoglyceric acid	225	ND	ND	ND	ND	ND	ND
2-Deoxyuridine	226	ND	ND	ND	ND	ND	ND
3-Methyl-2-oxovaleric acid	227	ND	ND	ND	ND	ND	ND
Cytosine	228	320	ND	680	131	190	460
Cer 41:1;2O|Cer 18:1;2O/23:0	229	ND	ND	ND	ND	ND	ND
Acetaminophen sulfate	230	ND	ND	ND	ND	ND	ND
2-Amino-3-methoxybenzoic acid	231	ND	ND	ND	ND	ND	ND
PC O-33:2	232	ND	ND	ND	ND	ND	ND
Dihydrocapsaicin	233	ND	ND	ND	ND	ND	ND
1,3-Dimethylbenzene-1-sulfonic acid	234	ND	ND	ND	ND	ND	ND
Pantothenic acid	235	187	1	42	423	91	ND
PC 35:5|PC 15:1_20:4	236	ND	ND	ND	ND	ND	ND
3,3-Dimethylacrylic acid	237	ND	ND	ND	ND	ND	ND
LPC O-20:0	238	ND	ND	ND	ND	ND	ND
1-Acetylimidazole	239	123	ND	ND	ND	ND	ND
Val-Gln	240	ND	ND	ND	ND	958	ND
Alanine	241	ND	353	336	ND	ND	ND
O1_PC p-36:4; or PC o-36:5	242	ND	ND	ND	ND	ND	ND
PI 38:5|PI 18:0_20:5	243	ND	ND	493	ND	ND	ND
PC 33:2|PC 15:0_18:2	244	ND	ND	ND	ND	ND	ND
2-(4-Morpholinyl)-1-phenylethanol	245	ND	ND	ND	ND	ND	ND
4-Hydroxyphenyllactic acid	246	ND	ND	ND	ND	ND	ND
1,3-Cyclohexanedicarboxylic acid	247	ND	ND	ND	ND	ND	ND
Carnosine	248	180	ND	158	ND	130	660
1-Methyl-L-histidine	249	ND	ND	ND	ND	ND	ND
N-Methylalanine	250	ND	203	367	76	ND	ND
5-Hydroxymethyl-6-methyluracil	251	ND	ND	ND	ND	ND	ND
N,N-Bis(2-hydroxyethyl)dodecanamide	252	89	172	179	ND	ND	ND
Undecanedioic acid	253	ND	ND	ND	ND	ND	ND
FA 16:2	254	ND	ND	454	ND	813	ND
2-Deoxyribose	255	ND	ND	ND	ND	ND	ND
2-dGMP	256	154	ND	ND	ND	ND	ND
Citraconic acid	257	ND	ND	ND	ND	ND	ND

TG 50:0 | TG 16:0_16:0_18:0	258	ND	ND	ND	ND	ND	ND
3'-O-Methylcytidine	259	ND	ND	ND	ND	ND	ND
2-Methylbutyryl-L-carnitine	260	ND	187	ND	ND	ND	ND
2-(9-Decenyl)glutaconic acid	261	ND	ND	ND	ND	ND	ND
Acetyl-beta-alanine	262	ND	ND	ND	ND	ND	ND
2'-O-Methyluridine	263	ND	ND	ND	ND	ND	ND
4-Hydroxyhippuric acid	264	48	ND	ND	ND	ND	ND
2-Methoxy-5-nitrophenol	265	ND	ND	ND	ND	ND	ND
5-Hydroxytryptophan	266	ND	ND	ND	ND	ND	239
Abscisic acid	267	ND	ND	ND	ND	ND	ND
Arabitol	268	ND	ND	ND	ND	ND	ND
3-Oxostearic acid	269	ND	ND	ND	ND	ND	ND
4-Hydroxy-6-methyl-2-pyrone	270	ND	ND	ND	ND	ND	ND
FA 18:2;2O	271	ND	ND	ND	ND	ND	ND
8-Hydroxyquinoline-2-carbaldehyde	272	ND	ND	ND	ND	ND	ND
Bradykinin	273	ND	ND	ND	ND	ND	ND
HexCer 40:1;2O | HexCer 18:1;2O/22:0	274	ND	ND	287	ND	ND	ND
2-Isopropylmalic acid	275	ND	ND	ND	ND	ND	ND
3-Hydroxy-3-methyl-2,3-dihydro-1H-indol-2-one	276	ND	ND	ND	ND	ND	ND
5-Butyl-1H-pyrazole-3-carboxylic acid	277	ND	ND	ND	ND	ND	ND
Glucosamine	278	ND	ND	ND	ND	ND	ND
2-Aminoisobutyric acid	279	ND	ND	ND	ND	ND	ND
3-Hydroxyoctadecanoic acid	280	ND	ND	ND	ND	ND	ND
LPE 16:0	281	ND	ND	171	365	1020	401
Betaine aldehyde	282	140	218	744	ND	311	ND
LPC 20:0/0:0	283	ND	ND	ND	ND	ND	ND
TG 58:12 | TG 18:2_18:4_22:6	284	ND	ND	665	ND	865	ND
Cer 34:1;2O | Cer 18:1;2O/16:0	285	ND	ND	ND	ND	ND	ND
Galactosamine-1-phosphate	286	ND	ND	ND	ND	ND	ND
Indoxyl sulfate	287	ND	ND	ND	ND	ND	ND
1,5-Anhydrosorbitol	288	ND	ND	ND	ND	ND	ND
1-Palmitoyl-2-azelaoylphosphatidylcholine	289	ND	ND	ND	ND	ND	917
3-Methylhistidine	290	83	ND	ND	ND	213	61
1,4-Butynediol	291	ND	ND	ND	ND	ND	ND
DG 32:0	292	293	200	ND	83	ND	455
3-Propyl-1H-pyrazole-5-carboxylic acid	293	ND	ND	ND	ND	ND	ND
PC 42:2	294	ND	ND	ND	ND	519	ND
FA 20:4;O	295	ND	ND	ND	ND	ND	ND
2-Hydroxyisobutyric acid	296	ND	ND	ND	ND	ND	ND
3-Carboxy-6-methylcoumarin	297	ND	ND	ND	ND	ND	ND
TG 52:0 | TG 16:0_18:0_18:0	298	ND	ND	61	ND	ND	ND
2-{2-[2-(Methacryloyloxy)ethoxy]ethoxy}ethyl 2-methylacrylate	299	ND	165	ND	ND	221	ND
Glutamic acid	300	238	334	316	353	ND	ND

FA 24:6	301	ND	ND	261	ND	133	ND
SL 32:0;0O	302	ND	ND	ND	ND	ND	ND
Linoleic acid	303	ND	ND	ND	ND	ND	ND
6-Methyl-2-[(2-oxo-2-phenylethyl)sulfanyl]-4(3H)-pyrimidinone	304	ND	ND	ND	ND	ND	ND
2'-Deoxycytidine	305	65	ND	ND	ND	116	916
Lys-Ala	306	68	ND	ND	ND	ND	420
2-(4-Fluorophenyl)acetohydrazide	307	ND	ND	ND	ND	ND	ND
FA 10:0_b	308	ND	ND	ND	ND	ND	ND
TG 60:10|TG 18:1_20:3_22:6	309	ND	ND	ND	ND	ND	ND
1-Stearoyl-2-hydroxy-sn-glycero-3-phosphocholine	310	ND	ND	ND	ND	ND	102
N,N-Dimethylarginine	311	556	371	351	403	265	432
Pyroglutamic acid	312	ND	392	533	449	ND	650
Homovanillic acid sulfate	313	ND	ND	ND	ND	ND	ND
Ala-Ala	314	111	ND	ND	ND	ND	864
Ala-Glu	315	317	ND	ND	ND	ND	110
3-Aminoisobutyric acid	316	ND	ND	ND	ND	ND	ND
Bisphenol A bis(2,3-dihydroxypropyl) ether	317	ND	ND	ND	ND	ND	ND
erythro-Sphingosine-1-phosphate	318	ND	ND	ND	ND	ND	ND
1,2-Dimethylimidazole	319	ND	ND	ND	ND	206	ND
Sorbose	320	ND	ND	ND	ND	ND	ND
3-Phenyllactic acid	321	ND	ND	ND	ND	ND	ND
Glutathione (reduced)	322	348	ND	ND	124	ND	278
2-(1H-Pyrazol-1-yl)benzylamine	323	ND	ND	ND	ND	ND	ND
3-Oxocholic acid	324	ND	ND	ND	ND	ND	ND
Tuberonic acid	325	ND	ND	ND	ND	ND	ND
3,3-Dimethylglutaric acid	326	ND	ND	ND	ND	ND	ND
3-Hydroxydodecanoic acid	327	ND	ND	ND	ND	ND	74
Serotonin	328	444	ND	ND	ND	ND	ND
FA 26:0;O	329	ND	ND	ND	ND	ND	ND
FA 22:2	330	512	ND	ND	308	ND	56
2-Hydroxyquinoline	331	ND	ND	ND	ND	ND	ND
TG 46:2|TG 12:0_16:0_18:2	332	ND	ND	ND	ND	ND	ND
PC O-36:3	333	ND	ND	ND	ND	535	ND
2-Oleoyl-1-palmitoyl-sn-glycero-3-phosphocholine	334	ND	ND	ND	ND	ND	ND
SM 34:1;2O|SM 18:1;2O/16:0	335	ND	ND	ND	ND	ND	ND
DG 36:4	336	23	48	ND	348	788	338
5-Hydroxyvalproic acid	337	ND	ND	ND	ND	ND	ND
FA 20:2	338	466	ND	ND	264	ND	909
LPC 20:1/0:0	339	ND	ND	ND	ND	ND	ND
N-Acetylalanine	340	ND	ND	ND	ND	ND	ND
3-Hydroxysebacic acid	341	ND	ND	ND	ND	ND	ND
LPC 15:0/0:0	342	ND	ND	ND	ND	ND	ND
PC 37:5;O|PC 20:4_17:1;O	343	ND	ND	ND	ND	ND	ND

1,2-dioleoyl-sn-glycero-3-phosphatidylcholine_1,2-dioleoyl-sn-glycero-3-phosphatidylcholine	344	ND	ND	ND	ND	ND	ND
FA 24:0;O	345	ND	ND	ND	ND	ND	ND
PC O-40:6	346	ND	ND	ND	ND	284	ND
2-(Formylamino)benzoic acid	347	ND	ND	ND	ND	ND	ND
PC O-38:4	348	ND	ND	ND	ND	540	ND
3-Galloylgallocatechin	349	ND	ND	ND	ND	ND	ND
Cholesterol	350	36	ND	ND	210	371	227
Betaine	351	363	291	341	92	77	151
3-Hydroxyanthranilic acid	352	ND	ND	ND	ND	ND	ND
4-Fluorobenzene-1,3-dicarboxylic acid	353	ND	ND	ND	ND	ND	ND
Homovanillic acid	354	ND	ND	ND	ND	ND	ND
4-Hydroxy-6-methylnicotinic acid	355	ND	ND	ND	ND	ND	ND
DG 34:2	356	361	66	ND	380	346	452
PC 36:4;3O|PC 18:2_18:2;3O	357	ND	ND	ND	ND	ND	ND
gamma-Glutamyl-alanine	358	ND	ND	ND	ND	ND	ND
LPC 22:0/0:0	359	ND	ND	ND	ND	ND	ND
7.125_186.1124	360	ND	ND	ND	ND	ND	ND
4-Hydroxybenzoic acid	361	ND	ND	ND	ND	ND	ND
6-Hydroxycaproic acid	362	ND	ND	ND	ND	ND	ND
Isoquinoline-3-carboxylic acid	363	ND	ND	ND	ND	ND	ND
Pipecolic acid	364	225	ND	ND	ND	ND	ND
N-Methylglutamic acid	365	77	ND	ND	ND	ND	447
Spermine	366	ND	ND	ND	ND	ND	ND
beta-Homoglutamine	367	ND	ND	ND	ND	76	ND
LPC O-18:0	368	ND	ND	ND	ND	ND	ND
DG 34:1	369	434	73	ND	4	19	453
Ethylmalonic acid	370	ND	ND	ND	ND	ND	ND
Hexaethylene glycol	371	ND	189	238	ND	390	ND
3,5-Dichlorosalicylic acid	372	ND	ND	ND	ND	ND	ND
TG O-55:9|TG O-13:1_21:4_21:4	373	ND	ND	ND	ND	ND	ND
4-Amino-2-methylbenzamide	374	ND	ND	ND	ND	ND	ND
8-(3-Octyl-2-oxiranyl)octanoic acid	375	ND	ND	ND	ND	ND	ND
LPC 23:0/0:0	376	ND	ND	ND	ND	ND	ND
Cer 42:0;2O|Cer 18:0;2O/24:0	377	ND	ND	ND	ND	ND	266
SM 32:1;2O	378	ND	ND	ND	ND	ND	ND
Oleoyl ethylamide	379	ND	ND	ND	ND	ND	ND
3-Methylcrotonylglycine	380	335	ND	ND	ND	ND	ND
Propionylcarnitine	381	200	ND	ND	426	411	631
Mannitol	382	ND	ND	ND	ND	ND	ND
Adenine	383	215	ND	ND	ND	ND	195
PC 39:6	384	422	ND	ND	190	82	558
PC O-40:7	385	ND	ND	ND	ND	349	ND
Guanidinoacetic acid	386	ND	ND	ND	ND	ND	323

PI 38:3 | PI 18:0_20:3	387	ND	ND	491	ND	ND	ND
2-Piperidinecarboxamide	388	ND	ND	724	ND	ND	ND
3-Methyladipic acid	389	ND	ND	ND	ND	ND	ND
N-(2-Furoyl)glycine	390	213	ND	ND	ND	ND	430
4-Hydroxyquinoline	391	236	ND	ND	98	ND	ND
O2_FA 24:1; (nervonic acid)	392	ND	ND	ND	ND	ND	ND
PI 39:4 | PI 19:0_20:4	393	ND	ND	ND	ND	41	ND
N-Acetylcytidine	394	ND	ND	ND	ND	ND	262
PE 34:2	395	ND	ND	ND	493	90	162
Methyl 4-hydroxycinnamate	396	ND	ND	ND	ND	ND	ND
4-Isoxazolepropanoic acid, alpha-amino-2,3-dihydro-5-methyl-3-oxo-	397	ND	ND	ND	ND	ND	ND
FA 21:5	398	ND	ND	374	ND	901	ND
N-acetyltryptophan	399	ND	ND	ND	ND	ND	ND
2-Acetylpyrazine	400	ND	ND	ND	ND	205	ND
Cysteic Acid	401	ND	ND	ND	ND	ND	ND
SM 40:2;2O | SM 15:1;2O/25:1	402	ND	ND	ND	ND	ND	ND
saccharic acid	403	ND	ND	ND	273	ND	ND
2(1H)-Pyridinone	404	ND	ND	ND	ND	ND	ND
P-Coumaric acid	405	ND	ND	ND	ND	ND	ND
Benzeneethanamine, 3,5-dimethoxy-.alpha.-methyl-4-propoxy-	406	ND	ND	ND	ND	ND	ND
epsilon-Dimethyl-lysine	407	ND	ND	ND	ND	ND	ND
Acamprosate	408	ND	ND	ND	ND	ND	ND
PC O-38:7	409	ND	ND	ND	ND	545	ND
3-Hydroxyoctanoic acid	410	ND	ND	ND	ND	ND	ND
SE 28:1/20:3	411	ND	ND	ND	ND	ND	ND
3'-O-Methylguanosine	412	130	ND	ND	ND	ND	ND
5-Methylcytidine	413	ND	ND	ND	ND	ND	ND
TG 60:12 | TG 18:1_20:5_22:6	414	ND	ND	ND	ND	ND	ND
LPC O-16:0	415	ND	ND	ND	ND	334	ND
N-Glycolylneuraminic acid	416	ND	ND	ND	ND	ND	ND
3-Hydroxypropanoic acid	417	ND	ND	ND	ND	ND	ND
2,8-Quinolinediol	418	ND	ND	ND	ND	ND	ND
PE 40:6 | PE 18:0_22:6	419	ND	ND	479	ND	588	ND
O1_FA 20:4; (arachidonic acid)	420	ND	ND	ND	ND	ND	ND
Guanidinopropionic acid	421	ND	ND	ND	ND	ND	ND
LPC 24:0/0:0	422	ND	ND	ND	ND	ND	ND
3-(Trifluoromethyl)cinnamic acid	423	ND	ND	ND	ND	ND	ND
SM 42:1;2O | SM 18:1;2O/24:0	424	ND	ND	ND	ND	ND	ND
Cystine	425	462	ND	679	362	ND	542
Isoxanthopterin	426	ND	ND	ND	ND	ND	ND
TG 58:12 | TG 18:2_20:5_20:5	427	ND	ND	ND	ND	ND	ND
3-Fluoro-5-(methoxycarbonyl)benzoic acid	428	ND	ND	ND	ND	ND	ND
PC O-41:11	429	ND	ND	ND	ND	ND	ND

Creatine	430	239	228	199	313	277	545
DG 34:2|DG 16:0_18:2	431	ND	ND	237	ND	ND	ND
PC O-31:7	432	ND	ND	ND	ND	ND	ND
N-Acetylneuraminic acid	433	338	ND	ND	ND	ND	461
CAR 4:0	434	ND	ND	ND	ND	682	ND
4-Hydroxybenzoic acid propyl ester	435	ND	ND	ND	ND	ND	ND
Citrulline	436	443	359	677	316	347	546
Palmitoyl sphingomyelin	437	ND	ND	ND	ND	ND	ND
Benzyldimethylstearylammonium	438	403	ND	126	ND	ND	ND
PC 40:4|PC 20:0_20:4	439	ND	ND	ND	ND	ND	ND
FA 10:0_a	440	ND	ND	ND	ND	ND	ND
N-Methylproline	441	ND	ND	ND	ND	ND	ND
Cysteinesulfinic acid	442	515	ND	ND	ND	ND	ND
5-Methyl-5,6-Dihydrouracil	443	ND	ND	ND	ND	ND	ND
Glycerophosphocholine	444	ND	279	319	63	308	325
Cer 43:1;2O|Cer 19:1;2O/24:0	445	ND	ND	ND	ND	ND	ND
3,4-Dihydro-3-oxo-2H-(1,4)-benzoxazin-2-ylacetic acid	446	ND	ND	ND	ND	ND	ND
NAE 14:0	447	ND	ND	ND	ND	ND	ND
Docosan-1-amine	448	ND	ND	133	ND	ND	ND
Cysteine-glutathione disulfide	449	425	ND	ND	ND	ND	ND
Glyceraldehyde	450	ND	ND	ND	ND	ND	ND
O4_FA 20:4; (arachidonic acid)	451	ND	ND	ND	ND	ND	ND
CAR 12:0	452	ND	ND	ND	ND	120	ND
PC 38:4|PC 18:0_20:4	453	ND	ND	ND	ND	ND	ND
SM 36:2;2O|SM 19:1;2O/17:1	454	ND	ND	ND	ND	ND	ND
Phenylacetylglycine	455	ND	ND	ND	ND	ND	ND
Cinnamic acid	456	ND	ND	ND	ND	ND	ND
5(Aminocarbonyl)amino]pentanoic acid	457	ND	ND	ND	ND	ND	ND
Cholic acid	458	ND	ND	ND	ND	ND	ND
Asp-Glu	459	ND	ND	ND	ND	ND	232
PC 32:2	460	96	ND	407	549	465	523
Cer 41:0;2O|Cer 18:0;2O/23:0	461	ND	ND	ND	ND	ND	ND
2-Methyl-4-phenylazophenylamine	462	ND	ND	ND	ND	ND	ND
SM 36:2;2O|SM 18:2;2O/18:0	463	ND	ND	ND	ND	ND	ND
Dodecanedioic acid	464	ND	ND	ND	ND	ND	ND
CE 18:3	465	ND	ND	ND	579	ND	ND
Glyceric acid	466	ND	ND	ND	ND	ND	ND
Arginine	467	366	380	338	ND	67	ND
FA 18:3	468	52	ND	ND	370	ND	923
glycerol-alpha-phosphate	469	174	ND	ND	298	1012	891
TG 55:7|TG 16:0_18:2_21:5	470	ND	ND	635	ND	843	762
Hypoxanthine	471	ND	317	274	427	983	378
Ser-Val	472	ND	ND	ND	ND	ND	ND

FA 15:1	473	160	ND	ND	163	ND	935
N,N-Dimethylformamide	474	460	ND	ND	ND	ND	ND
5-Methoxy-3-indoleacetic acid	475	ND	ND	ND	ND	ND	ND
Caffeic acid	476	ND	ND	ND	ND	ND	ND
N-Methyl-L-asparagine	477	ND	ND	366	ND	ND	ND
Gly-Val	478	ND	ND	ND	ND	933	ND
PC 32:2|PC 14:0_18:2	479	ND	ND	ND	ND	ND	ND
Hexadecanedioic acid	480	ND	ND	ND	ND	ND	ND
N-Acetylcysteine	481	ND	ND	ND	ND	ND	ND
4-Methylumbelliferone	482	ND	ND	ND	ND	ND	ND
7,8-Dihydrobiopterin	483	ND	ND	ND	ND	ND	ND
Histidine	484	253	385	307	ND	ND	379
Homogentisic acid	485	ND	ND	ND	ND	ND	ND
2,3-Dimethoxy-5-methylbenzoquinone	486	ND	ND	ND	ND	ND	ND
7-Nitro-2,1,3-benzoxadiazol-4-ylamine	487	ND	ND	ND	ND	ND	ND
Cysteine S-sulfate	488	ND	ND	ND	ND	ND	ND
Triethanolamine	489	ND	132	259	ND	ND	ND
Aleuritic acid	490	ND	ND	ND	ND	ND	ND
LPC 18:0	491	133	ND	206	237	405	408
N-Methylhistidine	492	299	ND	368	ND	ND	ND
3-Ureidopropionic acid	493	ND	ND	ND	ND	ND	ND
Octadecanedioic acid	494	ND	ND	ND	ND	ND	509
3-Acetoxypyridine	495	ND	ND	72	ND	ND	ND
Theanine	496	ND	ND	ND	ND	ND	ND
PE 38:6|PE 16:0_22:6	497	ND	ND	446	ND	ND	ND
FA 21:0	498	ND	ND	ND	ND	ND	ND
Malonyl-L-carnitine	499	ND	ND	ND	ND	ND	414
PC O-34:3	500	ND	ND	ND	ND	533	ND
5-Hydroxy-3,4-dihydro-2(1H)-quinolinone	501	ND	ND	ND	ND	ND	ND
Pro-Leu	502	534	ND	ND	ND	714	ND
N-Tigloylglycine	503	ND	ND	ND	ND	ND	ND
SM 32:1;2O|SM 18:1;2O/14:0	504	ND	ND	ND	ND	ND	ND
CAR 13:0	505	ND	ND	ND	ND	ND	ND
Malic acid	506	ND	ND	ND	ND	ND	ND
FA 14:1	507	132	ND	ND	249	245	936
N8-Acetylspermidine	508	38	ND	ND	ND	ND	440
LPI 18:0	509	ND	ND	303	ND	1044	ND
PE 38:5|PE 18:1_20:4	510	ND	ND	121	ND	ND	ND
Adenosine	511	ND	ND	ND	62	310	146
Abietic acid	512	ND	ND	ND	ND	ND	ND
Thymidine	513	ND	ND	ND	ND	ND	ND
SM 40:1;2O|SM 18:1;2O/22:0	514	ND	ND	ND	ND	ND	ND
LPC 20:4/0:0	515	ND	ND	ND	ND	ND	ND

Thiamine	516	566	ND	131	ND	946	ND
Homoarginine	517	ND	ND	ND	ND	ND	ND
Pro-Gly	518	539	ND	ND	ND	713	ND
Dihydro-4,4-dimethyl-2,3-furandione	519	ND	ND	ND	ND	ND	ND
Imidazole	520	379	ND	252	ND	ND	ND
FA 16:1	521	167	ND	ND	539	ND	933
Prolylphenylalanine	522	ND	ND	ND	ND	ND	ND
FA 18:3;O	523	ND	ND	ND	ND	ND	ND
Gentisinic acid	524	ND	ND	ND	ND	ND	ND
P-Toluenesulfonic acid	525	ND	ND	ND	ND	ND	ND
FA 18:4	526	ND	ND	178	ND	869	ND
4-Acetamidobutyric acid	527	124	ND	ND	ND	ND	ND
Resveratrol-3-O-sulfate	528	ND	ND	ND	ND	ND	ND
FA 18:0;O	529	ND	ND	ND	ND	ND	ND
Cer 40:0;2O | Cer 18:0;2O/22:0	530	ND	ND	ND	ND	ND	657
SM 41:2;2O	531	ND	ND	ND	ND	ND	ND
SM 33:1;2O	532	ND	ND	ND	ND	ND	ND
FA 28:7	533	ND	ND	8	ND	256	ND
CAR 20:1	534	ND	ND	ND	ND	269	ND
DG 36:5 | DG 18:2_18:3	535	ND	ND	ND	ND	ND	ND
2-Hydroxy-5-methoxybenzoic acid	536	ND	ND	ND	ND	ND	ND
PI 38:5 | PI 18:1_20:4	537	ND	ND	77	ND	ND	ND
TG 58:7 | TG 18:1_18:2_22:4	538	ND	ND	ND	ND	ND	787
4-Acetylbutyric acid	539	ND	ND	ND	ND	ND	ND
Isocitric acid lactone	540	ND	ND	ND	ND	ND	ND
LPC 18:2/0:0	541	ND	ND	ND	ND	ND	ND
3-Methylcytidine	542	138	ND	ND	ND	ND	ND
N-Acetyllysine	543	ND	ND	ND	ND	ND	ND
Orotic acid	544	ND	ND	ND	ND	ND	ND
Phenylalanine, methyl ester	545	ND	ND	ND	ND	711	ND
Guanine	546	ND	ND	ND	318	ND	291
5-Hydroxy-3-indoleacetic acid	547	ND	ND	ND	ND	ND	ND
FA 18:2;O	548	ND	ND	ND	ND	ND	ND
DG 40:7 | DG 18:1_22:6	549	ND	ND	432	ND	800	ND
Cer 34:2;2O | Cer 18:1;2O/16:1	550	ND	ND	ND	ND	ND	ND
Triphenyl phosphate	551	ND	ND	ND	ND	ND	ND
Glutaric acid	552	ND	ND	ND	ND	ND	ND
Cyclohexanamine	553	ND	ND	ND	ND	ND	ND
Docosahexanoic acid	554	ND	ND	ND	ND	ND	ND
N-Acetylmethionine	555	ND	ND	364	ND	1067	ND
Adipic acid	556	ND	ND	ND	ND	ND	ND
Leu-Leu	557	ND	ND	ND	ND	ND	ND
Anserine	558	311	322	337	88	585	113

N-Acetyl-epsilon-caprolactam	559	ND	ND	ND	ND	ND	ND
CAR 6:0	560	ND	ND	ND	ND	ND	ND
Indole-3-acetonitrile	561	ND	ND	ND	ND	ND	ND
Pseudouridine	562	ND	ND	ND	ND	ND	ND
PE 34:1 | PE 16:0_18:1	563	ND	ND	462	ND	560	ND
N-Acetylhistidine	564	533	395	363	ND	ND	ND
Ala-Arg	565	ND	ND	335	67	412	822
FA 20:3	566	519	ND	ND	180	ND	908
FA 25:0_b	567	ND	ND	ND	ND	ND	ND
Picolinuric acid	568	ND	ND	ND	ND	ND	ND
Taurine	569	184	379	283	ND	943	ND
LPC O-18:1	570	ND	ND	ND	ND	1015	ND
TG 55:8 | TG 15:0_18:2_22:6	571	ND	ND	ND	ND	844	ND
PC 38:6	572	266	ND	438	305	497	108
PI 38:4;O | PI 18:0_20:4;O	573	ND	ND	ND	ND	ND	ND
TG 62:12 | TG 18:1_22:5_22:6	574	ND	ND	695	ND	894	ND
Phosphocholine	575	314	ND	ND	254	ND	ND
TG 56:8 | TG 16:0_18:2_22:6	576	ND	ND	647	ND	ND	ND
Benzoylmalic acid	577	ND	ND	ND	ND	ND	ND
PC 40:6 | PC 18:0_22:6	578	ND	ND	ND	ND	ND	ND
Cer 42:2;2O | Cer 18:1;2O/24:1	579	ND	ND	ND	ND	ND	ND
LPC 24:1/0:0	580	ND	ND	ND	ND	ND	ND
LPE O-18:2	581	ND	ND	114	ND	1039	ND
TG 43:2;1O | TG 10:0_16:0_17:2;1O	582	ND	ND	ND	ND	ND	ND
LPC 17:1/0:0	583	ND	ND	ND	ND	ND	ND
Icaridin	584	ND	ND	ND	ND	ND	ND
Diisodecyl phthalate	585	ND	ND	ND	ND	ND	ND
PC 34:0	586	309	ND	417	531	384	521
PC O-36:1	587	ND	ND	ND	ND	ND	ND
Gly-Gly	588	ND	ND	ND	ND	ND	ND
LPC 20:5/0:0	589	ND	ND	ND	ND	ND	ND
O2_PE 38:2 isomer b	590	ND	ND	ND	ND	ND	ND
FA 23:0	591	ND	ND	ND	ND	ND	ND
Gabapentin	592	ND	ND	ND	ND	ND	ND
Hydroferulic acid	593	ND	ND	ND	ND	ND	ND
DG 50:1	594	ND	ND	ND	ND	ND	ND
PC 39:4 | PC 19:0_20:4	595	ND	ND	ND	ND	ND	ND
O1_FA 20:5; (eicosapentaenoic acid)	596	ND	ND	ND	ND	ND	ND
N-Acetylmannosamine	597	ND	ND	ND	ND	ND	ND
Ala-Ile	598	245	ND	ND	ND	ND	ND
PC 36:2	599	290	ND	424	529	6	537
PC O-36:6	600	ND	ND	ND	ND	177	ND
Palitantin	601	ND	ND	ND	ND	ND	ND

4-Hydroxyvalproic acid	602	ND	ND	ND	ND	ND	ND
O2_PE 38:2	603	ND	ND	241	ND	ND	ND
LPC 18:3/0:0	604	ND	ND	ND	ND	ND	ND
Cedrin	605	ND	ND	ND	ND	ND	ND
Ethyl sulfate	606	ND	ND	ND	ND	ND	ND
Zinterol	607	ND	ND	ND	ND	ND	ND
4-Pyridoxic acid	608	ND	ND	ND	26	ND	ND
5'-S-Methyl-5'-thioadenosine	609	ND	ND	ND	ND	ND	ND
FA 22:5;O	610	ND	ND	ND	ND	ND	ND
SM 35:2;3O	611	ND	ND	ND	ND	ND	ND
SM 42:3;2O	612	ND	ND	ND	ND	ND	ND
PC O-37:4	613	ND	ND	ND	ND	ND	ND
Acetohydroxamic acid	614	ND	ND	ND	ND	ND	ND
Cer 33:1;2O|Cer 17:1;2O/16:0	615	ND	ND	ND	ND	ND	ND
SE 29:1/20:4	616	ND	ND	ND	ND	ND	ND
PI 38:4|PI 18:0_20:4	617	ND	ND	492	ND	ND	ND
PI 36:2|PI 18:0_18:2_a	618	ND	ND	ND	ND	ND	ND
Putrescine	619	ND	110	504	ND	ND	ND
Galactosamine	620	525	ND	ND	198	ND	944
Hexanoyl-L-carnitine	621	569	ND	ND	ND	977	365
LPC 22:6/0:0	622	ND	ND	ND	ND	ND	ND
TG 47:0|TG 15:0_16:0_16:0	623	ND	ND	198	ND	243	ND
PC O-36:5|PC O-16:1_20:4	624	ND	ND	ND	ND	ND	ND
FA 20:5	625	42	ND	ND	196	ND	905
Acetyl-carnitine	626	ND	ND	ND	ND	ND	ND
Hexanoylglycine	627	ND	ND	ND	ND	ND	ND
PC 32:0|PC 16:0_16:0	628	ND	ND	ND	ND	ND	ND
4.94_805.56	629	ND	ND	ND	ND	ND	ND
Epigallocatechin	630	571	ND	ND	ND	396	ND
Cer 39:1;2O|Cer 17:1;2O/22:0	631	ND	ND	ND	ND	ND	284
Serine	632	ND	387	562	388	759	681
LPE 22:6	633	ND	ND	51	351	1031	ND
Lauroyl-L-carnitine	634	ND	ND	ND	ND	ND	ND
Methylacetate	635	ND	ND	ND	ND	ND	ND
Maleic acid	636	ND	ND	ND	ND	ND	ND
Normetanephrine	637	ND	ND	ND	ND	ND	ND
SM 38:2;2O|SM 18:2;2O/20:0	638	ND	ND	ND	ND	ND	ND
PC O-37:8	639	ND	ND	ND	ND	539	ND
LPC 18:1	640	136	ND	286	543	996	407
PE O-37:4|PE O-21:3_16:1	641	ND	ND	ND	ND	ND	ND
Ketoisovaleric acid	642	ND	ND	ND	ND	ND	ND
N-Cinnamoylglycine	643	ND	ND	ND	ND	ND	ND
PI 34:1|PI 16:0_18:1	644	ND	ND	ND	ND	ND	ND

Xanthosine	645	ND	ND	ND	289	ND	ND
Tetradecanedioic acid	646	ND	ND	ND	ND	ND	ND
FA 18:1;O	647	ND	ND	ND	ND	ND	ND
Propionic acid	648	ND	ND	ND	ND	ND	ND
PC O-38:2	649	ND	ND	ND	ND	ND	ND
Malonic acid	650	ND	ND	ND	ND	ND	ND
phosphoethanolamine	651	197	ND	ND	321	226	857
SM 42:2;2O|SM 19:1;2O/23:1	652	ND	ND	ND	ND	ND	ND
SM 42:3;2O|SM 14:1;2O/28:2	653	ND	ND	ND	ND	ND	ND
Catechol	654	ND	ND	ND	ND	ND	ND
His-Pro	655	380	ND	169	387	244	357
TG 48:2|TG 14:0_16:0_18:2	656	ND	ND	ND	ND	ND	ND
SM 42:2;2O|SM 18:1;2O/24:1	657	ND	ND	ND	ND	ND	ND
Glufosinate	658	ND	ND	ND	ND	ND	ND
Indole-3-carboxaldehyde	659	ND	ND	ND	ND	ND	ND
PC O-32:1;1O|PC O-17:0_15:1;1O	660	ND	ND	ND	ND	ND	ND
PC 36:0	661	ND	ND	ND	ND	479	539
PE 36:4|PE 16:0_20:4	662	ND	ND	15	ND	ND	ND
LPC 16:0/0:0	663	ND	ND	ND	ND	ND	ND
TG 46:2|TG 14:0_16:1_16:1	664	ND	ND	ND	ND	ND	ND
Cer 43:1;2O|Cer 18:1;2O/25:0	665	ND	ND	ND	ND	ND	609
3'-O-Methylinosine	666	ND	ND	ND	ND	ND	ND
Kynurenic acid	667	ND	ND	ND	ND	ND	ND
5.10_807.07	668	ND	ND	ND	ND	ND	ND
TG O-56:6|TG O-18:0_16:0_22:6	669	ND	ND	ND	ND	ND	ND
4-Guanidinobutyric acid	670	182	20	27	105	266	ND
gamma-Aminobutyric acid	671	ND	ND	239	ND	ND	ND
Pyridoxamine	672	53	375	505	369	ND	627
FA 34:1	673	ND	ND	138	ND	32	ND
Corticosterone	674	ND	ND	ND	ND	ND	ND
DG 50:0	675	ND	ND	ND	ND	ND	ND
Ribonic acid gamma-lactone	676	ND	ND	ND	ND	ND	ND
LPI 20:4	677	ND	ND	304	ND	1049	ND
PE 38:4|PE 18:0_20:4	678	ND	ND	468	ND	ND	ND
Psicose	679	ND	ND	ND	ND	ND	ND
CAR 20:5	680	ND	ND	ND	ND	335	ND
Thr-His	681	ND	ND	ND	ND	ND	795
LPC 22:5 a	682	ND	ND	ND	ND	ND	ND
S-Adenosyl-homocysteine	683	10	ND	534	ND	722	648
Tetrabutylammonium	684	ND	ND	ND	ND	ND	ND
DG 34:3	685	ND	176	ND	23	780	451
Dexpanthenol	686	47	ND	55	ND	ND	ND
Cer 46:0;2O|Cer 22:0;2O/24:0	687	ND	ND	ND	ND	ND	ND

PI 36:4 | PI 16:0_20:4_b	688	ND	ND	ND	ND	ND	ND
TG 58:11 | TG 16:0_20:5_22:6	689	ND	ND	ND	ND	ND	ND
PC O-42:6	690	ND	ND	ND	ND	ND	ND
CMPF	691	ND	ND	ND	ND	ND	ND
Stearamide	692	ND	ND	70	ND	ND	ND
Glu-Thr	693	461	ND	ND	127	ND	313
FA 20:3;O	694	ND	ND	ND	ND	ND	ND
Decanoyl-L-carnitine	695	ND	ND	ND	ND	810	ND
TG 52:4;2O | TG 16:0_18:1_18:3;2O	696	ND	ND	ND	ND	ND	ND
LPC O-16:1	697	ND	ND	ND	ND	1013	ND
PC 38:2	698	ND	ND	193	302	491	564
Uracil	699	ND	350	627	30	ND	815
SM 42:2;2O | SM 18:2;2O/24:0	700	ND	ND	ND	ND	ND	ND
O1_FA 20:5; (eicosapentaenoic acid)_b	701	ND	ND	ND	ND	ND	ND
LPC 19:1/0:0	702	ND	ND	ND	ND	ND	ND
Ethylhexadecyldimethylammonium	703	ND	ND	ND	ND	ND	ND
N-Isovalerylglycine	704	ND	ND	ND	ND	ND	ND
Kynurenine	705	ND	ND	ND	ND	ND	ND
P-Anisic acid	706	ND	ND	ND	ND	ND	ND
Vitamin C	707	ND	ND	ND	ND	ND	ND
LPE 18:1	708	ND	ND	301	59	1024	399
Sebacic acid	709	ND	ND	ND	ND	ND	ND
N-Formylmethionine	710	ND	ND	ND	ND	ND	ND
Norleucine	711	ND	ND	ND	ND	ND	ND
PC 36:2;O | PC 18:0_18:2;O	712	ND	ND	ND	ND	ND	ND
N-(4-Hydroxy-3-methylphenyl)acetamide	713	ND	ND	ND	ND	ND	ND
LPE 18:2	714	35	ND	147	573	366	398
DG 36:3	715	13	149	ND	322	785	339
Thiazolidine-4-carboxylic acid	716	94	ND	ND	ND	ND	ND
Urocanic acid	717	ND	ND	32	ND	ND	ND
N-Isobutyrylglycine	718	ND	ND	ND	ND	ND	ND
N,N-Dimethyldodecylamine N-oxide	719	ND	288	257	ND	ND	ND
PC 35:2 | PC 17:0_18:2	720	ND	ND	ND	ND	ND	ND
FA 18:2	721	16	ND	ND	509	ND	926
Cer 43:0;2O | Cer 20:0;2O/23:0	722	ND	ND	ND	ND	ND	ND
N1-(3-Aminopropyl)-N1-methylpropane-1,3-diamine	723	ND	ND	ND	ND	ND	ND
4,5,7-Trihydroxyisoflavone	724	ND	ND	ND	ND	ND	ND
Indolelactic acid	725	ND	ND	ND	ND	ND	ND
PC O-36:2	726	ND	ND	ND	ND	ND	ND
Phenylacetaldehyde	727	ND	ND	ND	ND	ND	ND
PC O-34:1	728	ND	ND	ND	ND	530	ND
PC 38:6 | PC 16:0_22:6	729	ND	ND	ND	ND	ND	ND
FA 17:1	730	ND	ND	146	ND	829	ND

N-Acetyl-D-glucosamine	731	565	ND	ND	374	ND	423
FA 20:4;2O	732	ND	ND	ND	ND	ND	ND
LPE 18:0	733	384	ND	328	104	1023	400
2-Mercaptomethylbenzimidazole	734	ND	ND	124	ND	ND	ND
SM 34:1;3O	735	ND	ND	ND	ND	ND	ND
Mandelic acid	736	ND	ND	ND	ND	ND	ND
Acetylcarnitine	737	416	ND	ND	134	372	ND
TG 60:7 | TG 18:1_20:1_22:5	738	ND	ND	720	ND	ND	ND
Pyridoxal phosphate	739	ND	ND	ND	ND	ND	ND
PC 38:4 | PC 18:1_20:3	740	ND	ND	ND	ND	ND	ND
PC 38:5 | PC 16:0_22:5	741	ND	ND	451	ND	ND	ND
PI 36:5 | PI 16:0_20:5	742	ND	ND	ND	ND	673	ND
Myristoyl-L-carnitine	743	487	ND	ND	ND	ND	ND
Thr-Gln	744	567	ND	ND	ND	949	796
CAR 20:4	745	ND	ND	ND	ND	648	ND
FA 22:6	746	ND	ND	ND	ND	ND	ND
gamma-Glutamylmethionine	747	272	ND	ND	ND	ND	ND
LPC 22:5 b	748	ND	ND	ND	ND	ND	ND
PE 34:2 | PE 16:0_18:2	749	ND	ND	60	ND	ND	ND
FA 17:0	750	ND	ND	ND	335	ND	4
Creatinine	751	ND	42	678	ND	ND	544
PC 36:3 | PC 18:1_18:2	752	ND	ND	ND	ND	ND	ND
Proline	753	ND	388	501	ND	ND	ND
PC O-38:4 | PC O-18:0_20:4	754	ND	ND	ND	ND	ND	ND
LPE O-16:1	755	ND	ND	136	ND	1036	ND
Indole-3-carboxylic acid	756	ND	ND	ND	ND	ND	ND
PC O-32:2	757	ND	ND	ND	ND	ND	ND
FA 32:1	758	ND	ND	ND	ND	ND	ND
Ribose	759	ND	ND	ND	ND	ND	ND
DMSO	760	102	320	ND	ND	ND	ND
LPC 20:2/0:0	761	ND	ND	ND	ND	ND	ND
LPC 20:3/0:0	762	ND	ND	ND	ND	ND	ND
PC O-38:6 | PC O-16:0_22:6	763	ND	ND	ND	ND	ND	ND
PC O-34:3 | PC O-16:1_18:2	764	ND	ND	ND	ND	ND	ND
O1_PC p-38:4; or PC o-38:5; B	765	ND	ND	ND	ND	ND	ND
Allantoin	766	ND	ND	ND	ND	ND	ND
Proline-hydroxyproline	767	ND	ND	ND	ND	ND	632
Salicylic acid	768	ND	ND	ND	ND	ND	ND
Palmitoylcarnitine	769	ND	ND	ND	ND	705	ND
N-alpha-methylhistamine	770	ND	ND	ND	ND	ND	ND
PC O-36:4 | PC O-16:0_20:4	771	ND	ND	ND	ND	ND	ND
TG 45:0 | TG 14:0_15:0_16:0	772	ND	ND	ND	ND	ND	773
CAR 14:0	773	ND	ND	ND	ND	599	ND

5.10_807.57	774	ND	ND	ND	ND	ND	ND
N-Acetyl-valine	775	ND	ND	ND	ND	ND	ND
Pyridoxal	776	ND	ND	ND	206	721	628
N-(1H-Indol-3-ylacetyl)glycine	777	ND	ND	ND	ND	ND	431
Thr-Glu	778	ND	ND	ND	ND	ND	ND
CAR 14:1	779	ND	ND	ND	ND	17	ND
Ferulic acid	780	ND	ND	ND	ND	ND	ND
SM 34:0;2O	781	ND	ND	ND	ND	ND	ND
PC O-37:5	782	ND	ND	ND	ND	ND	ND
Octanoylcarnitine	783	ND	ND	ND	ND	458	ND
FA 18:1	784	504	ND	ND	292	ND	694
TG O-55:2 | TG O-19:1_16:0_20:1	785	ND	ND	ND	ND	ND	ND
FA 27:0	786	ND	ND	ND	ND	ND	ND
PC O-39:10	787	ND	ND	ND	ND	193	ND
Val-Gly	788	ND	ND	ND	ND	ND	ND
N-Acetylleucine	789	ND	ND	ND	ND	ND	ND
FA 36:0	790	ND	ND	ND	ND	ND	ND
PC 41:6 | PC 19:0_22:6	791	ND	ND	ND	ND	514	ND
Cer 40:2;2O | Cer 18:2;2O/22:0	792	ND	ND	ND	ND	ND	656
LPC 22:4/0:0	793	ND	ND	ND	ND	ND	ND
TMAO	794	9	33	ND	ND	ND	ND
PC O-40:4	795	ND	ND	ND	ND	ND	ND
Ribulofuranose	796	ND	ND	ND	ND	ND	ND
TG 58:9 | TG 18:1_18:2_22:6	797	ND	ND	703	ND	ND	ND
PI 36:3 | PI 18:1_18:2	798	ND	ND	36	ND	ND	ND
SM 38:1;2O	799	ND	ND	ND	ND	ND	ND
TG O-60:3 | TG O-22:1_18:1_20:1	800	ND	ND	ND	ND	ND	ND
PC 38:5 | PC 18:1_20:4	801	ND	ND	ND	ND	ND	ND
delta-Hydroxylysine	802	ND	ND	ND	ND	ND	ND
ST 24:1;O5	803	ND	ND	ND	ND	ND	ND
TG 60:11 | TG 18:1_20:4_22:6	804	ND	ND	ND	ND	ND	ND
Daidzein	805	ND	ND	ND	ND	ND	941
Dihydrouracil	806	ND	ND	ND	ND	ND	ND
FA 20:4	807	ND	ND	ND	ND	ND	907
PC 39:6 | PC 17:0_22:6	808	ND	ND	ND	ND	11	ND
FA 22:0	809	475	ND	ND	274	ND	902
TG 53:0 | TG 16:0_18:0_19:0	810	ND	ND	ND	ND	ND	ND
PI 36:3 | PI 16:0_20:3	811	ND	ND	ND	ND	ND	ND
PC 38:6 | PC 18:2_20:4	812	ND	ND	ND	ND	ND	ND
PE 40:7 | PE 18:1_22:6	813	ND	ND	480	ND	589	ND
PC O-38:5 | PC O-18:1_20:4	814	ND	ND	ND	ND	ND	ND
TG O-62:7 | TG O-22:1_18:0_22:6	815	ND	ND	ND	ND	ND	ND
PE O-38:6 | PE O-16:0_22:6	816	ND	ND	ND	ND	ND	ND

Lys-Leu	817	19	ND	ND	ND	1058	ND
Indole-3-acetamide	818	ND	ND	ND	ND	ND	ND
O3_PC 35:2	819	ND	ND	ND	ND	ND	ND
Uridine	820	141	ND	ND	269	ND	ND
SM 40:2;2O	821	ND	ND	ND	ND	ND	ND
N-epsilon-Acetyllysine	822	473	ND	ND	ND	ND	443
Hippuric acid	823	ND	ND	ND	ND	ND	364
PE O-36:5 | PE O-16:1_20:4	824	ND	ND	76	ND	ND	ND
N-(4-Methoxyphenyl)acetamide	825	ND	ND	ND	ND	ND	ND
CAR 18:2	826	ND	ND	ND	ND	641	ND
PE P-38:6 | PE P-16:0_22:6	827	ND	ND	ND	ND	640	ND
PE 36:1 | PE 18:0_18:1	828	ND	ND	464	ND	564	ND
FA 19:1	829	ND	ND	397	ND	878	ND
PC O-34:4	830	ND	ND	ND	ND	ND	ND
TG 60:9 | TG 18:1_18:2_24:6	831	ND	ND	ND	ND	ND	ND
FA 22:1	832	464	ND	ND	355	ND	900
PI 36:3	833	ND	ND	ND	ND	670	619
TG 58:8 | TG 18:1_18:1_22:6	834	ND	ND	675	ND	ND	ND
SM 40:2;2O | SM 18:2;2O/22:0	835	ND	ND	ND	ND	ND	ND
PC 40:5 | PC 18:0_22:5	836	ND	ND	ND	ND	ND	ND
CAR 16:2	837	ND	ND	ND	ND	423	ND
PC 34:3 | PC 16:1_18:2	838	ND	ND	ND	ND	ND	ND
FA 22:5	839	ND	ND	353	ND	175	ND
TG O-51:0 | TG O-17:0_16:0_18:0	840	ND	ND	ND	ND	ND	ND
FA 26:0	841	ND	ND	ND	ND	ND	ND
PC 34:1 | PC 16:0_18:1	842	ND	ND	ND	ND	ND	ND
PI 36:1 | PI 18:0_18:1_b	843	ND	ND	ND	ND	ND	ND
N-Acetylphenylalanine	844	ND	ND	ND	ND	ND	ND
Riboflavin	845	22	ND	ND	66	ND	649
TG 46:0 | TG 14:0_16:0_16:0	846	ND	ND	129	ND	ND	ND
ST 27:1;O;S	847	ND	ND	ND	ND	ND	ND
TG O-48:0 | TG O-17:0_15:0_16:0	848	ND	ND	200	ND	ND	ND
SM 34:2;2O | SM 18:2;2O/16:0	849	ND	ND	ND	ND	ND	ND
PC 36:3 | PC 16:0_20:3	850	ND	ND	ND	ND	ND	ND
PC 36:2 | PC 18:1_18:1	851	ND	ND	ND	ND	ND	ND
Salicylic alcohol	852	ND	ND	ND	ND	ND	ND
PC 32:1 | PC 16:0_16:1	853	ND	ND	ND	ND	ND	ND
m-Toluthioamide	854	ND	ND	ND	ND	ND	ND
Glycitein	855	ND	ND	ND	ND	ND	ND
PC 36:1 | PC 18:0_18:1	856	ND	ND	ND	ND	ND	ND
TG O-60:7 | TG O-20:0_18:1_22:6	857	ND	ND	ND	ND	ND	ND
LPE 20:4	858	159	ND	228	342	1030	397
Iminodiacetic acid	859	ND	ND	ND	ND	ND	ND

TG 54:0 | TG 18:0_18:0_18:0	860	ND	ND	ND	ND	ND	ND
FA 9:0	861	ND	ND	ND	10	ND	ND
PI 38:6 | PI 16:0_22:6	862	ND	ND	ND	ND	681	ND
Apigenin 7-glucuronide	863	ND	ND	ND	ND	ND	ND
N-Methylvaline	864	ND	ND	ND	ND	ND	ND
PC O-38:6	865	ND	ND	ND	ND	543	ND
PI 40:6	866	ND	ND	ND	ND	686	615
PC O-34:2	867	ND	ND	ND	ND	532	ND
Daidzein 4'-sulfate	868	ND	ND	ND	ND	ND	ND
PC 34:2;O | PC 16:0_18:2;O_b	869	ND	ND	ND	ND	ND	ND
FA 24:1	870	516	ND	ND	258	ND	892
PI 40:6 | PI 18:0_22:6	871	ND	ND	526	ND	ND	ND
TG 58:0 | TG 16:0_18:0_24:0	872	ND	ND	ND	ND	ND	ND
TG 60:8 | TG 18:1_20:1_22:6	873	ND	ND	722	ND	889	ND
TG 46:0 | TG 14:0_15:0_17:0	874	ND	ND	ND	ND	ND	ND
PC 40:8 | PC 18:2_22:6	875	ND	ND	ND	ND	ND	ND
FA 20:1	876	191	ND	ND	359	ND	918
DG 36:3 | DG 18:1_18:2	877	ND	ND	267	ND	ND	ND
PC 36:5 | PC 16:0_20:5	878	ND	ND	425	ND	ND	ND
Pro-Gln	879	117	ND	ND	424	712	638
Tuberonic acid glucoside	880	ND	ND	ND	ND	ND	ND
TG O-58:4 | TG O-22:1_18:1_18:2	881	ND	ND	ND	ND	ND	ND
TG 58:7 | TG 18:0_18:1_22:6	882	ND	ND	672	ND	ND	ND
SM 39:1;2O	883	ND	ND	ND	ND	ND	ND
PC 34:2 | PC 16:0_18:2	884	ND	ND	ND	ND	ND	ND
PE O-34:3 | PE O-16:1_18:2	885	ND	ND	455	ND	ND	ND
PC 37:2	886	ND	ND	426	534	37	530
SM 42:3;2O | SM 18:2;2O/24:1	887	ND	ND	ND	ND	ND	ND
PI 36:4	888	ND	ND	ND	ND	671	ND
CAR 16:1	889	ND	ND	ND	ND	605	ND
PC 40:7 | PC 18:1_22:6	890	ND	ND	ND	ND	ND	ND
PC 35:1 | PC 17:0_18:1	891	ND	ND	ND	ND	ND	ND
FA 28:0	892	ND	ND	ND	ND	ND	ND
O2_FA 20:2; (eicosadienoic acid)	893	ND	ND	ND	ND	ND	ND
PE O-38:5 | PE O-18:1_20:4	894	ND	ND	495	ND	619	ND
PC O-41:10	895	ND	ND	ND	ND	ND	ND
PC 33:1 | PC 16:0_17:1	896	ND	ND	ND	ND	ND	ND
TG 54:5 | TG 18:1_18:2_18:2	897	ND	ND	ND	ND	ND	ND
10.42_369.35	898	ND	ND	ND	ND	ND	ND
TG O-54:0 | TG O-20:0_16:0_18:0	899	ND	ND	ND	ND	ND	ND
TG O-57:0 | TG O-17:0_18:0_22:0	900	ND	ND	ND	ND	ND	ND
PE O-36:2 | PE O-18:0_18:2	901	ND	ND	ND	ND	ND	ND
PE O-42:7 | PE O-20:1_22:6	902	ND	ND	471	ND	ND	ND

PE O-38:7 | PE O-16:1_22:6	903	ND	ND	497	ND	ND	ND
N-Carbobenzyloxyleucine	904	ND	ND	ND	ND	ND	ND
TG O-52:0 | TG O-18:0_16:0_18:0	905	ND	ND	ND	ND	ND	ND
PC O-36:5	906	ND	ND	ND	ND	536	ND
PC O-36:4	907	ND	ND	ND	ND	321	ND
PE O-40:7 | PE O-18:1_22:6	908	ND	ND	499	ND	629	ND
TG 59:11 | TG 16:0_21:5_22:6	909	ND	ND	ND	ND	ND	ND
DG 34:1 | DG 16:0_18:1	910	ND	ND	262	ND	ND	ND
methionine	911	ND	ND	ND	ND	1037	ND
TG O-58:3 | TG O-22:1_18:0_18:2	912	ND	ND	ND	ND	ND	ND
O2_FA 20:1; (eicosenoic acid)	913	ND	ND	ND	ND	ND	ND
TG O-50:3 | TG O-16:0_16:0_18:3	914	ND	ND	ND	ND	ND	ND
PC 36:4 | PC 18:2_18:2	915	ND	ND	ND	ND	ND	ND
TG O-57:13 | TG O-21:5_18:4_18:4	916	ND	ND	ND	ND	ND	ND
PE O-36:3 | PE O-18:1_18:2	917	ND	ND	ND	ND	ND	ND
Formononetine	918	ND	ND	ND	ND	ND	ND
CAR 18:1	919	ND	ND	ND	ND	626	ND
TG 52:4;1O | TG 16:0_18:2_18:2;1O	920	ND	ND	ND	ND	ND	ND
PE O-40:4 | PE O-20:0_20:4	921	ND	ND	ND	ND	ND	ND
Valine	922	550	ND	710	ND	ND	809
Methioninesulfoxide	923	ND	ND	350	ND	103	436
PC 37:2 | PC 19:0_18:2	924	ND	ND	ND	ND	ND	ND
TG O-52:1 | TG O-18:0_16:0_18:1	925	ND	ND	ND	ND	ND	ND
TG 60:9 | TG 20:1_18:2_22:6	926	ND	ND	723	ND	ND	ND
TG O-56:1 | TG O-20:0_18:0_18:1	927	ND	ND	ND	ND	ND	ND
TG O-52:2 | TG O-18:0_16:0_18:2	928	ND	ND	ND	ND	ND	ND
Arg-Leu	929	30	ND	ND	ND	357	238
TG 60:1 | TG 16:0_26:0_18:1	930	ND	ND	687	ND	ND	ND
TG 54:9 | TG 18:2_18:3_18:4	931	ND	ND	ND	ND	ND	ND
TG O-50:2 | TG O-16:0_16:0_18:2	932	ND	ND	ND	ND	ND	ND
TG 66:3 | TG 16:0_32:1_18:2	933	ND	ND	ND	ND	ND	ND
TG 49:2 | TG 15:0_16:0_18:2	934	ND	ND	25	ND	ND	ND
TG O-59:3 | TG O-19:1_20:1_20:1	935	ND	ND	ND	ND	ND	ND
PE O-40:5 | PE O-18:0_22:5	936	ND	ND	ND	ND	624	ND
TG 52:6 | TG 16:1_18:2_18:3	937	ND	ND	ND	ND	1087	ND
TG 52:7 | TG 14:0_18:2_20:5	938	ND	ND	ND	ND	ND	ND
TG 52:7 | TG 18:2_18:2_16:3	939	ND	ND	ND	ND	151	765
TG 54:7 | TG 18:2_18:2_18:3	940	ND	ND	ND	ND	ND	ND
TG 54:3 | TG 18:1_18:1_18:1	941	ND	ND	ND	ND	ND	ND
TG 54:2 | TG 16:0_18:1_20:1	942	ND	ND	ND	ND	ND	ND
TG 54:2 | TG 18:0_18:1_18:1	943	ND	ND	618	ND	ND	ND
TG 54:0 | TG 16:0_18:0_20:0	944	ND	ND	ND	ND	ND	ND
TG 53:4 | TG 17:1_18:1_18:2	945	ND	ND	613	ND	ND	ND

TG 53:5 | TG 17:1_18:2_18:2	946	ND	ND	614	ND	ND	ND
TG 53:6 | TG 17:1_18:2_18:3	947	ND	ND	615	ND	826	ND
TG 53:3 | TG 17:0_18:1_18:2	948	ND	ND	612	ND	ND	ND
TG 53:2 | TG 16:0_18:1_19:1	949	ND	ND	643	ND	ND	ND
TG 53:1 | TG 16:0_19:0_18:1	950	ND	ND	642	ND	ND	ND
TG 53:4 | TG 17:0_18:2_18:2	951	ND	ND	ND	ND	ND	ND
TG 52:8 | TG 18:2_18:2_16:4	952	ND	ND	641	ND	ND	ND
TG 50:5 | TG 14:0_18:2_18:3	953	ND	ND	584	ND	ND	ND
TG 50:5 | TG 16:0_18:2_16:3	954	ND	ND	ND	ND	ND	ND
TG 50:6 | TG 16:0_18:2_16:4	955	ND	ND	585	ND	ND	ND
Prostaglandin A3	956	ND	ND	ND	ND	ND	ND
Ala-Asn	957	ND	ND	ND	ND	ND	850
Asn-Ala	958	ND	ND	ND	ND	ND	ND
BA 24:1;O3;T	959	ND	ND	ND	ND	ND	ND
TG 49:3 | TG 15:0_16:1_18:2	960	ND	ND	89	ND	ND	ND
TG 49:0 | TG 15:0_16:0_18:0	961	ND	ND	ND	ND	ND	ND
TG 48:1 | TG 14:0_16:0_18:1	962	ND	ND	570	ND	ND	ND
Liquiritigenin	963	ND	ND	ND	ND	ND	ND
Ala-Leu-Ala-Leu	964	ND	ND	ND	ND	ND	ND
O1_FA 22:6; (docosahexaenoic acid)	965	ND	ND	ND	ND	ND	ND
O1_PC 36:4; A	966	ND	ND	ND	ND	ND	ND
Tri-2-ethylhexyl trimellitate	967	ND	ND	ND	ND	ND	ND
o-Hydroxyhippuric acid	968	ND	ND	ND	ND	ND	ND
Tauroursodeoxycholic acid	969	ND	ND	ND	296	ND	800
Lys-Ile	970	ND	ND	ND	ND	ND	ND
SL 33:0;0O	971	ND	ND	ND	ND	ND	ND
TG 52:4 | TG 16:0_16:0_20:4	972	ND	ND	ND	ND	ND	ND
TG 52:1 | TG 16:0_18:0_18:1	973	ND	ND	599	ND	ND	ND
TG 51:5 | TG 15:0_18:2_18:3	974	ND	ND	597	ND	ND	ND
TG 52:6 | TG 16:0_18:2_18:4	975	ND	ND	ND	ND	ND	ND
TG 52:6 | TG 16:0_16:1_20:5	976	ND	ND	ND	ND	ND	ND
TG 52:6 | TG 14:0_16:0_22:6	977	ND	ND	ND	ND	ND	ND
TG 52:5 | TG 16:0_16:0_20:5	978	ND	ND	ND	ND	ND	ND
TG 52:4 | TG 16:0_18:2_18:2	979	ND	ND	ND	ND	ND	ND
TG 51:1 | TG 16:0_17:0_18:1	980	ND	ND	625	ND	ND	ND
TG 51:3 | TG 16:0_17:1_18:2	981	ND	ND	ND	ND	ND	ND
TG 51:0 | TG 16:0_17:0_18:0	982	ND	ND	624	ND	ND	766
TG 51:4 | TG 15:0_18:2_18:2	983	ND	ND	ND	ND	ND	ND
TG 50:1 | TG 16:0_16:0_18:1	984	ND	ND	278	ND	ND	ND
TG 56:1 | TG 16:0_22:0_18:1	985	ND	ND	ND	ND	ND	ND
PI 37:4 | PI 17:0_20:4	986	ND	ND	201	ND	674	ND
TG 56:0 | TG 16:0_18:0_22:0	987	ND	ND	ND	ND	ND	ND
PE O-38:4 | PE O-18:0_20:4	988	ND	ND	ND	ND	616	ND

Chrysogine	989	ND	ND	676	ND	ND	ND
TG 59:2 | TG 16:0_25:0_18:2	990	ND	ND	ND	ND	ND	ND
TG 58:6 | TG 18:0_18:1_22:5	991	ND	ND	671	ND	ND	ND
TG 58:6 | TG 18:1_20:1_20:4	992	ND	ND	ND	ND	ND	ND
TG 59:1 | TG 16:0_25:0_18:1	993	ND	ND	704	ND	874	ND
TG 59:4 | TG 23:0_18:2_18:2	994	ND	ND	683	ND	ND	ND
Oroxyloside	995	ND	ND	ND	ND	ND	506
TG 58:4 | TG 22:0_18:2_18:2	996	ND	ND	ND	ND	ND	ND
TG 58:3 | TG 22:0_18:1_18:2	997	ND	ND	668	ND	ND	ND
TG 58:2 | TG 22:0_18:1_18:1	998	ND	ND	ND	ND	ND	ND
TG 58:2 | TG 16:0_18:1_24:1	999	ND	ND	667	ND	ND	ND
TG 57:8 | TG 18:1_18:2_21:5	1000	ND	ND	660	ND	860	ND
TG 58:1 | TG 16:0_24:0_18:1	1001	ND	ND	666	ND	ND	ND
PE O-40:6 | PE O-18:0_22:6	1002	ND	ND	ND	ND	627	ND
TG 55:2 | TG 16:0_21:0_18:2	1003	ND	ND	ND	ND	ND	ND
Phe-Leu	1004	ND	ND	ND	ND	ND	ND
O1_FA 18:3; (linolenic acid)	1005	ND	ND	ND	ND	ND	ND
Pencolide	1006	ND	ND	ND	ND	ND	ND
TG 55:3 | TG 19:0_18:1_18:2	1007	ND	ND	631	ND	ND	ND
TG 56:2 | TG 16:0_18:1_22:1	1008	ND	ND	ND	ND	ND	ND
TG 56:4 | TG 18:1_20:1_18:2	1009	ND	ND	71	ND	ND	ND
TG 56:5 | TG 16:0_18:1_22:4	1010	ND	ND	ND	ND	ND	ND
TG 56:5 | TG 18:0_18:1_20:4	1011	ND	ND	ND	ND	ND	ND
TG 56:5 | TG 20:1_18:2_18:2	1012	ND	ND	ND	ND	ND	ND
Isobutyric acid	1013	ND	ND	ND	ND	ND	ND
L-Glutarylcarnitine	1014	ND	ND	ND	259	ND	390
TG 57:1 | TG 16:0_23:0_18:1	1015	ND	ND	651	ND	ND	ND
TG 57:2 | TG 16:0_23:0_18:2	1016	ND	ND	ND	ND	ND	ND
TG 57:3 | TG 21:0_18:1_18:2	1017	ND	ND	ND	ND	856	791
TG 57:4 | TG 21:0_18:2_18:2	1018	ND	ND	ND	ND	ND	ND
TG 57:8 | TG 17:1_18:1_22:6	1019	ND	ND	ND	ND	ND	ND
TG 55:6 | TG 16:0_18:1_21:5	1020	ND	ND	634	ND	842	ND
TG 55:5 | TG 19:1_18:2_18:2	1021	ND	ND	633	ND	841	763
TG O-53:6 | TG O-15:0_19:3_19:3	1022	ND	ND	ND	ND	ND	ND
TG O-50:1 | TG O-16:0_16:0_18:1	1023	ND	ND	ND	ND	ND	ND
TG 62:3 | TG 26:0_18:1_18:2	1024	ND	ND	701	ND	ND	ND
TG O-52:3 | TG O-16:0_18:1_18:2	1025	ND	ND	ND	ND	ND	ND
TG 62:4 | TG 20:1_24:1_18:2	1026	ND	ND	ND	ND	ND	ND
Carbanilide	1027	ND	ND	ND	ND	ND	ND
TG 60:5 | TG 24:1_18:2_18:2	1028	ND	ND	755	ND	ND	ND
Inosine	1029	ND	309	309	416	ND	373
TG 60:2 | TG 18:0_24:0_18:2	1030	ND	ND	ND	ND	ND	ND
TG 60:3 | TG 24:0_18:1_18:2	1031	ND	ND	689	ND	ND	ND

TG 60:4 | TG 18:1_24:1_18:2	1032	ND	ND	690	ND	ND	ND
TG 61:3 | TG 25:0_18:1_18:2	1033	ND	ND	693	ND	892	ND
PC 38:2 | PC 20:0_18:2	1034	ND	ND	ND	ND	ND	ND
TG O-54:1 | TG O-20:0_16:0_18:1	1035	ND	ND	ND	ND	ND	ND
PC 36:4;O | PC 16:0_20:4;O	1036	ND	ND	ND	ND	ND	ND
Gln-Ser	1037	ND	ND	ND	ND	927	135
TG O-54:2 | TG O-20:0_16:0_18:2	1038	ND	ND	ND	ND	ND	ND
TG O-54:3 | TG O-18:0_18:1_18:2	1039	ND	ND	ND	ND	ND	ND
TG O-56:2 | TG O-20:0_18:0_18:2	1040	ND	ND	ND	ND	ND	ND
TG O-56:3 | TG O-20:0_18:1_18:2	1041	ND	ND	ND	ND	ND	ND
TG O-56:4 | TG O-19:2_18:2_19:0	1042	ND	ND	ND	ND	ND	ND
Gln-Ala	1043	508	ND	ND	ND	159	317
TG O-57:2 | TG O-19:0_18:1_20:1	1044	ND	ND	ND	ND	ND	ND
TG O-57:1 | TG O-19:1_16:0_22:0	1045	ND	ND	ND	ND	ND	ND
Glycocholic acid	1046	ND	ND	ND	ND	ND	ND
Glycyltyrosine	1047	ND	ND	ND	ND	ND	324
TG O-59:2 | TG O-19:0_18:2_22:0	1048	ND	ND	ND	ND	ND	ND
TG O-61:3 | TG O-19:1_20:1_22:1	1049	ND	ND	ND	ND	ND	ND
alpha.-Methylkynurenine	1050	ND	ND	ND	ND	ND	ND
Xanthine	1051	ND	360	737	507	ND	808
(1R,6S)-Bicyclo[4.1.0]heptane-7-carboxylic acid	ND	ND	128	ND	ND	ND	ND
(2.beta.,3.alpha.,5.alpha.,16.beta.,17.beta.)-2-(4-Morpholinyl)-16-(1-pyrrolidinyl)androstane-3,17-diol	ND	120	ND	ND	ND	ND	ND
(2E)-2-Cyano-3-(4-morpholinyl)-2-propenamide	ND	ND	ND	ND	47	ND	ND
(2E,4E)-Hexa-2,4-dienoic acid	ND	135	ND	ND	ND	ND	ND
(2S)-pyrrolidine-2-carboxylic acid	ND	ND	62	ND	ND	ND	ND
(3-Aminophenyl)acetonitrile	ND	ND	ND	46	ND	ND	ND
(3-Carboxypropyl)trimethylammonium_a	ND	ND	ND	ND	ND	166	ND
(3-Nitrophenyl)methanamine	ND	ND	94	56	ND	ND	39
(5S)-1,2,3,4,5,6,7,8,9,10-Decahydro-5,9-methanobenzo[8]annulen-11-one	ND	ND	ND	ND	ND	ND	20
(E)-.alpha.-Ionone	ND	ND	91	45	ND	ND	ND
(R)-Pantetheine	ND	ND	ND	ND	38	ND	ND
(Z)-9,12,13-Trihydroxyoctadec-15-enoic acid	ND	ND	ND	65	ND	ND	ND
.beta.-Methylphenethylamine	ND	ND	137	ND	ND	ND	ND
.gamma.-Aminobutyric acid	ND	ND	106	ND	ND	ND	ND
1,2,3,3-Tetramethyl-norbornan-2-ol	ND	ND	ND	137	ND	ND	ND
1,2,3,3-Tetramethylbicyclo[2.2.1]heptan-2-ol	ND	ND	168	ND	ND	ND	ND
1,2,4-benzenetriol	ND	ND	ND	ND	44	194	ND
1,2-Bis(O-decanoyl)-sn-glyceryl-3-phosphorylcholine	ND	ND	ND	ND	ND	ND	88
1,2-Cyclohexanedimethanol, 1,2-dimethanesulfonate, (1R,2R)-	ND	ND	141	ND	ND	ND	ND
1,2-Di-(9Z,12Z,15Z-octadecatrienoyl)-sn-glycero-3-phosphocholine	ND	ND	ND	ND	ND	121	ND
1,2-Diamino-2-methylpropane	ND	ND	102	81	ND	200	ND
1,2-Diarachidonoyl-sn-glycero-3-phosphocholine	ND	ND	ND	ND	ND	227	81
1,2-Dilauroyl-sn-glycero-3-phosphatidylcholine	ND	ND	208	ND	ND	ND	ND

1,2-Dilinoleoylglycerol	ND	ND	ND	ND	ND	134	ND
1,2-anhydro-myo-inositol	ND	158	ND	ND	ND	ND	ND
1,3,5-Benzenetriol	ND	ND	ND	ND	ND	167	ND
1,4,7,10,13,16-Hexaoxacyclooctadecane	ND	ND	ND	105	ND	ND	ND
1,4,7,10-Tetraazacyclododecane	ND	ND	ND	101	ND	ND	ND
1,4-Cyclohexanedione	ND	ND	163	54	ND	ND	ND
1,5-Pentanediamine	ND	ND	310	727	ND	ND	ND
1,5-Pentanediamine_a	ND	ND	ND	ND	ND	219	ND
1,5-anhydroglucitol	ND	85	ND	ND	22	66	31
1-(1Z-Hexadecenyl)-sn-glycero-3-phosphocholine	ND	ND	ND	ND	ND	ND	46
1-(2,5-Dimethylphenoxy)-3-(4-morpholinyl)-2-propanol	ND	66	ND	ND	ND	ND	ND
1-(2-Aminopropoxy)-2-methoxyethane	ND	ND	ND	84	ND	ND	ND
1-(2-Hydroxyethyl)pyrazole	ND	ND	ND	10	ND	62	ND
1-(4-Piperidinyl)-2-pyrrolidinone	ND	ND	ND	93	ND	ND	ND
1-(Methylsulfonyl)-1,4-diazepane	ND	ND	ND	57	ND	ND	ND
1-Acetamidocyclopentane-1-carboxylic acid	ND	ND	179	ND	ND	ND	ND
1-Cyclobutyl-4-piperidinamine	ND	ND	ND	33	ND	ND	ND
1-Cycloheptyl-4-piperidinamine	ND	ND	196	78	ND	ND	ND
1-Docosahexaenoyl-2-stearoyl-sn-glycero-3-phosphocholine	ND	ND	ND	ND	ND	343	ND
1-Ethyl-3-piperidinamine	ND	ND	101	92	ND	ND	ND
1-Ethyl-N4,N5-dimethyl-1H-imidazole-4,5-dicarboxamide	ND	ND	ND	ND	ND	187	ND
1-Hexadecyl-2-(5Z,8Z,11Z,14Z-eicosatetraenoyl)-sn-glycero-3-phosphocholine	ND	ND	ND	ND	ND	ND	30
1-Hexadecyl-2-(9Z-octadecenoyl)-sn-glycero-3-phosphocholine	ND	ND	ND	ND	ND	ND	18
1-Methyl-1H-indole-3-carboxamide	ND	ND	ND	108	ND	ND	ND
1-Methyl-4-piperidinecarboxylic acid	ND	ND	ND	ND	ND	ND	55
1-Methylguanosine	ND	ND	ND	ND	52	ND	60
1-Methylhistamine	ND	ND	ND	ND	ND	111	ND
1-Naphthoic acid	ND	ND	88	153	ND	ND	ND
1-Nitro-3-(4-nitrophenoxy)benzene	ND	ND	ND	ND	ND	ND	62
1-Oleoyl-2-myristoyl-sn-glycero-3-phosphocholine	ND	ND	ND	ND	ND	106	10
1-Palmitoyl-2-arachidonoyl-sn-glycero-3-phosphoserine	ND	ND	ND	ND	ND	ND	82
1-Palmitoyl-2-docosahexaenoyl-sn-glycero-3-phosphocholine_b	ND	ND	ND	ND	ND	146	ND
1-Palmitoyl-2-glutaryl phosphatidylcholine	ND	ND	ND	ND	ND	ND	67
1-Stearoyl-2-arachidonoyl-sn-glycero-3-phospho-(1'-myo-inositol)	ND	ND	ND	ND	ND	101	ND
1-Stearoyl-2-arachidonoyl-sn-glycero-3-phosphoserine	ND	ND	ND	ND	ND	ND	69
1-Stearoyl-2-docosahexaenoyl-sn-glycerol	ND	103	ND	ND	ND	112	75
1-Stearoyl-2-linoleoyl-sn-glycero-3-phospho-L-serine	ND	ND	ND	ND	ND	ND	1
1-Stearoyl-2-myristoyl-sn-glycero-3-phosphocholine	ND	ND	ND	ND	ND	ND	22
1-Stearoyl-2-oleoyl-sn-glycero-3-phosphoethanolamine	ND	ND	ND	ND	ND	230	ND
1-Stearoylglycerol	ND	ND	ND	ND	56	ND	ND
1-kestose	ND	ND	ND	ND	ND	153	25
1-methylgalactose	ND	ND	ND	ND	ND	143	ND
1-monopalmitin	ND	ND	ND	ND	ND	189	ND

1-monostearin	ND	ND	ND	ND	ND	202	28
1.24_193.12	ND	67	ND	ND	ND	ND	ND
10-Aminodecanoic acid	ND	ND	41	21	ND	ND	ND
10-Undecynoic acid	ND	ND	219	244	ND	ND	ND
11,15-Dioxo-9S-hydroxy-5Z-prostenoic acid	ND	ND	ND	ND	85	ND	ND
13-cis-Retinol	ND	ND	ND	ND	32	ND	ND
15.alpha.-Hydroxyculmorin	ND	ND	170	ND	ND	ND	ND
1H-1,2,3-Benzotriazol-1-ylmethanol	ND	ND	ND	96	ND	ND	ND
1H-Indole-3-ethanamine, 5-methoxy-N,N-di-2-propen-1-yl-	ND	31	ND	ND	ND	ND	ND
2'-Deoxyguanosine	ND	74	ND	ND	ND	ND	112
2'-Deoxyguanosine 3'-monophosphate	ND	ND	ND	ND	ND	ND	73
2'-O-Methylinosine	ND	ND	ND	ND	ND	ND	172
2,3,4,5-Tetrahydro-1,4-benzothiazepine	ND	ND	ND	ND	ND	ND	253
2,3-dihydroxybutanoic acid	ND	ND	ND	ND	ND	ND	95
2,4-Dimethylnicotinic acid	ND	ND	ND	ND	ND	304	83
2,4-Dimethylthiazole-5-carboxylic acid	ND	ND	211	738	ND	ND	ND
2,4-hexadienedioic acid	ND	99	ND	ND	ND	ND	104
2,5-dihydroxypyrazine	ND	246	ND	ND	69	78	86
2,6-Di-tert-butyl-4-(4-morpholinylmethyl)phenol	ND	ND	158	ND	ND	ND	ND
2-(1H-Benzimidazol-2-ylthio)-N,N-dimethylethanamine	ND	231	ND	ND	ND	ND	ND
2-(2-Aminoethanesulfonyl)propane	ND	ND	ND	141	ND	ND	ND
2-(2-Anilino-2-oxoethoxy)benzoic acid	ND	ND	ND	ND	ND	ND	77
2-(2-Hydroxyphenyl)-1,3-thiazolidine-4-carboxylic acid	ND	ND	ND	205	ND	ND	ND
2-(2-Quinolyl)ethanol	ND	ND	160	ND	ND	ND	ND
2-(3,8-Dihydroxy-8-(hydroxymethyl)-3-methyl-2-oxodecahydroazulen-5-yl)propan-2-yl hexopyranoside	ND	ND	ND	ND	ND	ND	84
2-(5-Oxovaleryl)phosphatidylcholine	ND	ND	ND	ND	ND	ND	679
2-Amino-2-deoxy-D-glucopyranose	ND	ND	ND	ND	ND	ND	59
2-Amino-3-(2,4-diketo-6,7-dihydro-5H-cyclopenta[d]pyrimidin-1-yl)propionic acid	ND	ND	ND	ND	ND	165	ND
2-Amino-5-phenylpentanoic acid	ND	ND	224	260	ND	ND	ND
2-Amino-N,N-dimethylacetamide	ND	178	ND	ND	ND	ND	ND
2-Arachidonoyl-1-palmitoyl-sn-glycero-3-phosphoethanolamine	ND	ND	ND	ND	ND	ND	16
2-Arachidonoylglycerol	ND	ND	ND	ND	46	ND	65
2-Carbamoylpyridine-3-carboxylic acid	ND	370	ND	ND	ND	ND	883
2-Docosahexaenoyl-1-palmitoyl-sn-glycero-3-phosphoethanolamine	ND	ND	ND	ND	ND	ND	140
2-Docosahexaenoyl-1-stearoyl-sn-glycero-3-phosphoethanolamine	ND	ND	ND	ND	ND	209	49
2-Docosahexaenoyl-1-stearoyl-sn-glycero-3-phosphoserine	ND	ND	ND	ND	ND	174	ND
2-Ethoxy-2-oxoethyl 2-hydroxybenzoate	ND	ND	ND	94	ND	ND	ND
2-Hydroxy-4-(octyloxyl)benzophenone	ND	ND	ND	85	ND	ND	ND
2-LPC 18:2	ND	ND	ND	ND	51	ND	ND
2-Methylbutyryl-carnitine	ND	ND	ND	ND	ND	199	ND
2-Methylnicotinic acid	ND	84	ND	ND	ND	ND	ND
2-Nitrobenzylamine	ND	ND	ND	ND	113	ND	ND
2-Oleoyl-1-stearoyl-sn-glycero-3-phosphoserine	ND	ND	ND	ND	ND	232	100

2-Oxo-2,3-dihydro-3-pyridinecarboxylic acid	ND	ND	ND	ND	61	ND	ND
2-Palmitoyl-rac-glycerol	ND	ND	ND	ND	ND	ND	96
2-Palmitoylglycerol	ND	ND	ND	ND	81	ND	ND
2-Pyridone	ND	62	ND	ND	ND	ND	ND
2-[(Dimethylamino)methyl]cyclohexanone	ND	ND	ND	ND	ND	79	ND
2-[1-(Hydroxymethyl)propyl]-1H-isoindole-1,3(2H)-dione	ND	ND	ND	ND	ND	ND	826
2-deoxypentitol	ND	204	ND	ND	65	ND	111
2-deoxytetronic acid	ND	ND	ND	ND	ND	ND	58
2-ethylcaproic acid	ND	ND	ND	ND	ND	ND	41
2-hydroxybutanoic acid	ND	ND	ND	ND	82	ND	90
2-hydroxyglutaric acid	ND	116	ND	ND	37	85	99
2-hydroxypyrazinyl-2-propenoic acid ethyl ester	ND	ND	ND	ND	ND	170	ND
2-hydroxyvaleric acid	ND	ND	ND	ND	ND	ND	34
2-ketoglucose dimethylacetal	ND	181	ND	ND	48	126	101
2-monopalmitin	ND	ND	ND	ND	ND	89	ND
2-phosphoglyceric acid	ND	ND	ND	ND	ND	ND	79
2-picolinic acid	ND	ND	ND	ND	ND	323	89
3,3-Dimethylpyrrolidin-2-one	ND	ND	343	ND	ND	ND	ND
3,4,5-Trimethoxydihydrocinnamic acid	ND	ND	ND	214	ND	ND	ND
3,4-Difluorobenzenecarboximidamide	ND	ND	348	ND	ND	ND	ND
3,4-Dihydroxyphenylalanine	ND	291	ND	ND	ND	ND	ND
3,6-anhydro-D-galactose	ND	ND	ND	ND	54	162	38
3,6-anhydro-D-glucose	ND	ND	ND	ND	58	ND	ND
3-(2,4-Dimethoxyanilino)-3-oxopropanoic acid	ND	ND	ND	142	ND	ND	ND
3-(3-Methoxybenzyl)piperidine	ND	ND	ND	ND	ND	ND	121
3-(5-Chloro-1,3-benzothiazol-2-yl)propanoic acid	ND	ND	ND	ND	ND	ND	144
3-(6-Amino-9H-purin-9-yl)propanoic acid	ND	ND	115	75	ND	ND	ND
3-(Carboxymethyl)-1-(beta-D-glucopyranosyl)-1H-indole	ND	ND	ND	132	ND	ND	ND
3-Amino-4-methylbenzenesulfonic acid	ND	ND	ND	ND	ND	ND	167
3-Aminoadipic acid	ND	ND	ND	ND	ND	152	245
3-Aminonaphthalene-2-carboxylic acid	ND	ND	ND	ND	ND	ND	190
3-Aminopiperidine-2,6-dione	ND	ND	ND	ND	ND	100	128
3-Aminotyrosine	ND	207	ND	99	ND	ND	78
3-Chloro-2-[(4-methylphenyl)sulfanyl]aniline	ND	ND	364	507	ND	ND	ND
3-Cyclohexyl-1,1-dimethylurea	ND	ND	265	ND	ND	218	ND
3-Ethyl-2-piperazinone	ND	ND	ND	ND	ND	ND	207
3-Hydroxyhexadecanoylcarnitine	ND	ND	ND	ND	ND	212	ND
3-Hydroxyisovaleroylcarnitine	ND	125	ND	203	ND	239	ND
3-Hydroxyoleylcarnitine	ND	ND	ND	ND	ND	267	214
3-Hydroxypicolinic acid	ND	ND	ND	ND	ND	ND	779
3-Hydroxypyridine	ND	ND	ND	73	ND	ND	ND
3-Indoleacrylic acid	ND	ND	ND	ND	ND	ND	177
3-Methyl-L-histidine	ND	ND	198	191	ND	ND	ND

3-aminoisobutyric acid	ND	152	ND	ND	35	ND	130
3-hydroxy-3-methylglutaric acid	ND	220	ND	ND	ND	ND	ND
3-hydroxybutyric acid	ND	ND	ND	ND	99	220	109
3-hydroxypropionic acid	ND	171	ND	ND	94	ND	ND
3-phosphoglycerate	ND	ND	ND	ND	ND	ND	161
3.alpha.-Galactobiose	ND	ND	ND	ND	ND	ND	882
4',5-Dihydroxy-3',6,7-trimethoxyflavone	ND	ND	ND	ND	ND	397	ND
4,8-Dimethylquinolin-2-ol	ND	ND	368	ND	ND	ND	ND
4-(1H-Pyrazol-1-yl)butanoic acid	ND	ND	ND	ND	ND	ND	668
4-(2,6,6-Trimethyl-3-oxocyclohex-1-en-1-yl)butan-2-yl .beta.-D-glucopyranoside	ND	ND	365	ND	ND	ND	ND
4-(Allylamino)benzoic acid	ND	ND	ND	ND	ND	ND	914
4-Acetyl-L-phenylalanine	ND	ND	ND	ND	ND	ND	913
4-Amino-1-butanol	ND	ND	99	144	ND	ND	ND
4-Aminobenzoic acid	ND	46	252	508	ND	ND	ND
4-Aminomethylcyclohexanecarboxylic acid	ND	ND	253	509	ND	380	ND
4-Aminomethyltetrahydropyran	ND	ND	ND	ND	ND	235	ND
4-Cyano-D-phenylalanine	ND	ND	283	ND	ND	ND	ND
4-Hydroxyamphetamine	ND	ND	ND	219	ND	ND	ND
4-Hydroxymandelonitrile	ND	82	ND	ND	ND	ND	ND
4-Imidazoleacrylic acid	ND	ND	13	ND	ND	215	ND
4-Methyl-1H-pyrazole	ND	ND	255	342	ND	ND	ND
4-Methyl-1H-pyrazole_4-Methyl-1H-pyrazole	ND	ND	ND	ND	ND	127	ND
4-Methyl-5-thiazoleethanol?	ND	ND	ND	ND	ND	ND	44
4-Methylpiperidine-1-carboximidamide	ND	18	ND	ND	ND	ND	ND
4-Phenyl-1H-pyrazol-3-ylamine	ND	ND	ND	253	ND	ND	ND
4-Piperidinecarboxamide	ND	ND	ND	116	ND	ND	ND
4-Trifluoromethylpyrimidine-2-carbaldehyde	ND	ND	ND	447	ND	ND	ND
4-aminobutyric acid	ND	112	ND	ND	93	ND	ND
4-chlorophenylisobutylamine	ND	ND	ND	448	ND	ND	ND
4-hydroxybutyric acid	ND	ND	ND	ND	ND	342	156
5'-Methylthioadenosine	ND	192	ND	ND	138	ND	912
5'-deoxy-5'-methylthioadenosine	ND	ND	ND	ND	ND	228	ND
5,6-Dihydro-4H-pyrrolo[3,4-d]thiazole	ND	ND	ND	ND	ND	ND	911
5-(6-Methyl-7-oxooctyl)furan-2(5H)-one	ND	ND	ND	224	ND	ND	ND
5-(Galactosylhydroxy)-L-lysine	ND	ND	ND	ND	ND	ND	910
5-(Hydroxymethyl)-2-furaldehyde	ND	ND	ND	ND	ND	ND	122
5-Amino-1-.beta.-D-ribofuranosyl-1H-imidazole-4-carboxamide	ND	258	ND	ND	ND	ND	287
5-Aminoimidazole-4-carboxamide	ND	ND	ND	ND	201	ND	ND
5-Aminoorotic acid	ND	ND	ND	263	ND	ND	ND
5-Chloro-1H-indole-3-carbaldehyde	ND	ND	79	ND	ND	ND	ND
5-Hydroxymethylcytosine	ND	121	ND	ND	ND	ND	ND
5-Methoxy-2-nitrophenylamine	ND	ND	ND	ND	20	248	288
5-Methylisoxazol-3-amine	ND	ND	223	ND	ND	ND	ND

5-Methyluridine	ND	ND	ND	ND	ND	ND	9
5-aminovaleric acid	ND	ND	ND	ND	186	ND	221
6-Biopterin	ND	ND	ND	ND	117	ND	145
6-Methylnicotinic acid	ND	ND	ND	ND	ND	ND	200
6-Phenylthiomorpholin-3-one	ND	ND	377	ND	ND	ND	ND
6-deoxyglucitol	ND	ND	ND	ND	ND	ND	153
7-Keto-8-aminopelargonic acid	ND	44	70	ND	ND	ND	ND
7-Methylguanine	ND	26	ND	ND	ND	ND	ND
8-Methylcaffeine	ND	ND	113	19	ND	ND	ND
8-Nitro-7-methoxyisoquinoline	ND	ND	ND	ND	ND	ND	258
8-Oxo-2-deoxyadenosine	ND	374	61	ND	ND	ND	ND
8-Oxononanoic acid	ND	ND	299	248	ND	ND	ND
AC 02:0	ND	ND	ND	449	ND	ND	ND
AC 03:0	ND	ND	ND	110	ND	ND	ND
AC 06:0	ND	ND	ND	118	ND	ND	ND
AC 12:0	ND	235	ND	ND	ND	ND	ND
AC 14:0	ND	ND	ND	ND	ND	ND	175
AC 14:1	ND	20	ND	ND	ND	ND	ND
AC 16:0	ND	156	ND	172	ND	ND	176
AC 16:1	ND	ND	ND	266	ND	ND	127
AC 18:0	ND	217	ND	450	ND	ND	159
AC 18:1	ND	157	ND	270	ND	ND	97
AC 18:2	ND	421	ND	148	ND	ND	23
AC 2-Methylbutyryl-	ND	ND	ND	332	ND	ND	ND
AC 20:0	ND	ND	ND	ND	ND	ND	174
AC 20:1	ND	ND	ND	ND	ND	ND	70
AC 20:2	ND	ND	ND	ND	ND	ND	236
AC 20:3	ND	ND	ND	ND	ND	ND	134
AC 20:4	ND	ND	ND	ND	ND	ND	131
AC 21:0	ND	ND	ND	ND	ND	ND	119
AC 21:3	ND	ND	ND	ND	ND	ND	166
AC 22:0	ND	ND	ND	ND	ND	ND	209
AC 22:1	ND	ND	ND	ND	ND	ND	26
AC 22:2	ND	ND	ND	ND	ND	ND	103
AC 22:6	ND	ND	ND	ND	ND	ND	186
AC 24:0	ND	ND	ND	ND	ND	ND	187
AC 24:1	ND	ND	ND	ND	ND	ND	40
AC 24:2	ND	ND	ND	ND	ND	ND	192
AC 3-Hydroxybutyryl	ND	ND	ND	333	ND	ND	ND
AC Malonyl-	ND	ND	ND	334	ND	ND	ND
ALDOPC	ND	ND	ND	ND	87	ND	ND
AMP	ND	ND	ND	ND	132	ND	ND
Ac-Asp-Glu	ND	ND	ND	ND	ND	242	ND

Acetyl coenzyme A	ND	40	ND	ND	ND	ND	ND
Acetyl-L-carnitine	ND	ND	ND	ND	ND	ND	196
Acylcarnitine 18:3	ND	ND	ND	ND	119	ND	ND
Adenosine 5'-diphosphoribose	ND	ND	ND	ND	418	ND	263
Adenosine 5'-monophosphate	ND	ND	ND	ND	ND	ND	867
Adenosine-3-monophosphate	ND	ND	ND	ND	ND	ND	866
Adenylosuccinic acid	ND	190	ND	ND	84	ND	865
Adipoyl-L-carnitine	ND	ND	ND	ND	71	ND	197
Adrenochrome	ND	323	ND	ND	ND	ND	ND
Ala-Glu.1	ND	367	ND	ND	ND	ND	ND
Ala-Ile-Lys	ND	ND	ND	ND	ND	ND	848
Ala-Leu	ND	ND	ND	ND	ND	102	ND
Ala-Lys	ND	ND	ND	ND	112	383	847
Ala-Phe	ND	ND	ND	ND	ND	43	ND
Ala-Pro	ND	ND	ND	ND	ND	ND	846
Ala-Ser	ND	ND	ND	ND	ND	427	ND
Allopurinol riboside	ND	ND	ND	ND	ND	157	844
Amantadine	ND	ND	ND	ND	ND	ND	164
Amobarbital	ND	119	ND	ND	ND	ND	ND
Androstane-3,17-diol	ND	228	ND	ND	ND	ND	ND
Androsterone	ND	360	ND	ND	ND	ND	ND
Aniline	ND	ND	181	230	ND	ND	ND
Arachidonoylcarnitine	ND	ND	ND	ND	ND	268	ND
Arachidonoylglycine	ND	456	ND	ND	ND	ND	ND
Arg-Asn	ND	ND	ND	ND	ND	ND	843
Arg-Asp	ND	506	ND	ND	ND	ND	842
Arg-Gln	ND	ND	ND	ND	ND	ND	841
Arg-Glu	ND	404	ND	ND	79	263	ND
Arg-Gly	ND	410	ND	ND	ND	324	840
Arg-Met	ND	33	ND	ND	ND	ND	ND
Arg-Phe	ND	278	ND	ND	ND	ND	ND
Arg-Pro	ND	ND	ND	ND	ND	ND	168
Arg-Ser	ND	483	ND	ND	ND	ND	ND
Arg-Tyr	ND	143	ND	ND	ND	ND	ND
Arg-Val	ND	480	ND	ND	ND	421	213
Argininosuccinic acid	ND	521	ND	339	150	70	834
Asn-Arg	ND	ND	ND	ND	118	391	833
Asn-His	ND	ND	ND	ND	ND	414	832
Asn-Lys	ND	ND	ND	ND	ND	114	ND
Asp-Ala	ND	ND	ND	ND	ND	314	ND
Asp-Arg	ND	ND	ND	ND	ND	156	ND
Asp-His	ND	ND	ND	ND	ND	ND	831
Asparagine	ND	ND	ND	ND	ND	ND	830

Aspartic acid	ND	371	370	ND	ND	ND	829
Avobenzone	ND	282	ND	ND	ND	ND	ND
Azelaic acid	ND	ND	ND	175	ND	ND	ND
Benzyl alcohol	ND	ND	339	340	ND	ND	ND
Benzyldimethyltetradecylammonium	ND	ND	248	90	ND	ND	ND
Benzyltrimethylammonium	ND	ND	ND	80	ND	ND	ND
Biotin	ND	490	ND	ND	ND	ND	ND
Bis(2-ethylhexyl) adipate	ND	ND	ND	139	ND	ND	ND
Butyrylcarnitine	ND	ND	ND	ND	ND	147	ND
CAR 14:2	ND	ND	ND	ND	ND	322	ND
CAR 15:0	ND	ND	ND	ND	ND	378	ND
CAR 16:0	ND	ND	ND	ND	ND	604	ND
CAR 17:0	ND	ND	ND	ND	ND	608	ND
CAR 17:1	ND	ND	ND	ND	ND	612	ND
CAR 18:3	ND	ND	ND	ND	ND	97	ND
CAR 20:0	ND	ND	ND	ND	ND	296	ND
CAR 20:2	ND	ND	ND	ND	ND	646	ND
CAR 20:3	ND	ND	ND	ND	ND	647	ND
CAR 22:1	ND	ND	ND	ND	ND	191	ND
CAR 22:2	ND	ND	ND	ND	ND	662	ND
CAR 22:4	ND	ND	ND	ND	ND	663	ND
CAR 22:5	ND	ND	ND	ND	ND	664	ND
CAR 22:6	ND	ND	ND	ND	ND	665	ND
CAR 24:5	ND	ND	ND	ND	ND	117	ND
CDP ethanolamine	ND	ND	ND	ND	225	ND	ND
CE 18:1	ND	170	ND	ND	45	ND	828
CE 18:2	ND	ND	ND	ND	222	ND	827
CE 20:4	ND	389	ND	ND	ND	ND	292
CE 20:5	ND	ND	ND	ND	354	ND	825
CE 22:2	ND	ND	ND	ND	299	ND	824
CE 22:6	ND	328	ND	ND	226	ND	823
CL 68:2 | CL 16:0_16:0_18:1_18:1	ND	ND	ND	187	ND	ND	ND
CL 68:2 | CL 16:0_18:1_16:0_18:1	ND	ND	ND	ND	ND	693	ND
CL 68:3 | CL 16:0_18:1_16:0_18:2	ND	ND	ND	ND	ND	704	ND
CL 70:4 | CL 16:0_18:1_18:1_18:2	ND	ND	ND	ND	ND	706	ND
CL 70:5 | CL 16:0_18:2_18:1_18:2	ND	ND	ND	ND	ND	709	ND
CL 70:6 | CL 16:1_18:2_18:1_18:2	ND	ND	ND	ND	ND	710	ND
CL 70:7 | CL 16:1_18:2_18:2_18:2	ND	ND	ND	ND	ND	716	ND
CL 72:5 | CL 18:1_18:1_18:1_18:2	ND	ND	ND	ND	ND	717	ND
CL 72:6 | CL 18:1_18:2_18:1_18:2	ND	ND	ND	ND	ND	718	ND
CL 72:7 | CL 18:1_18:2_18:2_18:2	ND	ND	ND	595	ND	719	ND
CL 72:8 | CL 18:2_18:2_18:2_18:2	ND	ND	ND	564	ND	4	ND
CL 74:11 | CL 16:1_18:2_18:2_22:6	ND	ND	ND	ND	ND	115	ND

CL 74:7 | CL 18:1_18:2_18:1_20:3	ND	ND	ND	ND	ND	22	ND
CL 74:8 | CL 16:1_20:3_18:1_20:3	ND	ND	ND	ND	ND	732	ND
CL 74:9 | CL 18:2_18:2_18:2_20:3	ND	ND	ND	ND	ND	733	ND
CL 76:10 | CL 18:1_18:2_18:1_22:6	ND	ND	ND	ND	ND	738	ND
CL 76:11 | CL 18:1_18:2_18:2_22:6	ND	ND	ND	ND	ND	742	ND
CL 76:12 | CL 18:2_18:2_18:2_22:6	ND	ND	ND	ND	ND	544	ND
CL 80:15 | CL 18:1_22:6_18:2_22:6	ND	ND	ND	ND	ND	360	ND
CL 80:16 | CL 18:2_22:6_18:2_22:6	ND	ND	ND	ND	ND	744	ND
Carbamic acid, N-(2,2-diphenylacetyl)-, ethyl ester	ND	ND	237	ND	ND	ND	ND
Castanospermine	ND	58	ND	ND	ND	ND	36
Cer 34:1;3O | Cer 18:1;2O/16:0;(2OH)	ND	ND	ND	183	ND	ND	ND
Cer 34:2;2O | Cer 18:2;2O/16:0	ND	ND	ND	ND	ND	ND	659
Cer 34:3;2O | Cer 18:2;2O/16:1	ND	ND	ND	ND	ND	ND	202
Cer 36:2;2O | Cer 18:2;2O/18:0	ND	ND	ND	ND	ND	ND	87
Cer 37:2;2O | Cer 18:2;2O/19:0	ND	ND	ND	ND	ND	ND	199
Cer 38:2;2O | Cer 18:2;2O/20:0	ND	ND	ND	ND	ND	ND	107
Cer 39:2;2O | Cer 18:2;2O/21:0	ND	ND	ND	ND	ND	ND	120
Cer 40:0;3O | Cer 17:0;2O/23:0;O	ND	ND	ND	ND	ND	ND	138
Cer 41:2;2O | Cer 18:2;2O/23:0	ND	ND	ND	ND	ND	ND	655
Cer 42:0;3O | Cer 17:0;2O/25:0;O	ND	ND	ND	ND	ND	ND	653
Cer 42:0;3O | Cer 18:0;3O/24:0	ND	ND	ND	176	ND	ND	ND
Cer 42:2;2O | Cer 18:2;2O/24:0	ND	ND	ND	ND	ND	ND	610
Cer 42:2;3O | Cer 18:1;2O/24:1;(2OH)	ND	ND	ND	134	ND	ND	ND
Cer 42:3;2O | Cer 18:2;2O/24:1	ND	ND	ND	ND	ND	ND	92
Cer 42:4;2O | Cer 22:3;2O/20:1	ND	ND	ND	ND	ND	ND	132
Cer 43:2;2O | Cer 18:1;2O/25:1	ND	ND	ND	ND	ND	ND	608
Cer 44:0;3O | Cer 18:0;3O/26:0	ND	ND	ND	156	ND	ND	ND
Cer 44:1;2O | Cer 20:1;2O/24:0	ND	ND	ND	ND	ND	ND	47
Cer 44:2;2O | Cer 18:2;2O/26:0	ND	ND	ND	ND	ND	ND	42
Cer d32:1	ND	ND	ND	ND	122	ND	607
Cer d33:1	ND	ND	ND	229	57	ND	606
Cer d34:0	ND	ND	ND	ND	86	ND	605
Cer d34:0 | Cer 18:0;2O/16:0	ND	ND	ND	566	ND	313	ND
Cer d34:1	ND	449	ND	ND	31	ND	604
Cer d34:1 | Cer 18:1;2O/16:0	ND	ND	ND	567	ND	135	ND
Cer d34:2	ND	241	107	215	142	ND	603
Cer d35:1 | Cer 17:1;2O/18:0	ND	ND	ND	568	ND	48	ND
Cer d36:0 | Cer 18:0;2O/18:0	ND	ND	ND	ND	ND	746	ND
Cer d36:1	ND	494	ND	ND	253	ND	216
Cer d36:1 | Cer 18:1;2O/18:0	ND	ND	ND	272	ND	749	ND
Cer d36:2 | Cer 18:2;2O/18:0	ND	ND	ND	149	ND	750	ND
Cer d37:1 | Cer 18:1;2O/19:0	ND	ND	ND	ND	ND	29	ND
Cer d38:0 | Cer 20:0;2O/18:0	ND	ND	ND	210	ND	251	ND

Cer d38:1	ND	346	ND	ND	77	ND		602
Cer d38:1 | Cer 18:1;2O/20:0	ND	ND	ND	265	ND	755	ND	
Cer d38:2 | Cer 18:2;2O/20:0	ND	ND	ND	6	ND	ND	ND	
Cer d39:1	ND	ND	ND	ND	151	ND		601
Cer d40:0	ND	ND	ND	ND	236	ND	ND	
Cer d40:1	ND	297	ND	ND	219	ND		448
Cer d40:1 | Cer 18:1;2O/22:0	ND	ND	ND	739	ND	764	ND	
Cer d40:2	ND	ND	ND	ND	24	765		551
Cer d40:2 | Cer 18:2;2O/22:0	ND	ND	ND	208	ND	ND	ND	
Cer d41:0 | Cer 18:0;2O/23:0	ND	ND	ND	170	ND	ND	ND	
Cer d41:1	ND	386	ND	ND	101	ND		550
Cer d41:1 | Cer 18:1;2O/23:0	ND	ND	ND	524	ND	10	ND	
Cer d41:2 | Cer 18:2;2O/23:0	ND	ND	ND	24	ND	ND	ND	
Cer d42:0	ND	ND	ND	ND	ND	119	ND	
Cer d42:0 | Cer 18:0;2O/24:0	ND	ND	ND	223	ND	ND	ND	
Cer d42:1	ND	572	ND	ND	336	ND		549
Cer d42:1 | Cer 18:1;2O/24:0	ND	ND	ND	741	ND	5	ND	
Cer d42:2	ND	426	ND	ND	314	ND		548
Cer d42:2 | Cer 18:1;2O/24:1	ND	ND	ND	277	ND	63	ND	
Cer d42:3 | Cer 18:1;2O/24:2	ND	ND	ND	ND	ND	183	ND	
Cer d42:3 | Cer 18:2;2O/24:1	ND	ND	ND	743	ND	ND	ND	
Cer d43:0 | Cer 17:0;2O/26:0	ND	ND	ND	ND	ND	98	ND	
Cer d43:1	ND	ND	64	168	ND	ND		547
Cer d43:1 | Cer 17:1;2O/26:0	ND	ND	ND	ND	ND	139	ND	
Cer d44:0 | Cer 18:0;2O/26:0	ND	ND	ND	112	ND	125	ND	
Cer d44:1	ND	ND	ND	ND	ND	279	ND	
Cer d44:1 | Cer 18:1;2O/26:0	ND	ND	ND	197	ND	ND	ND	
Cinnamaldehyde	ND	230	ND	ND	ND	ND	ND	
CoQ10	ND	ND	ND	ND	ND	247	ND	
CoQ8	ND	ND	ND	ND	ND	185	ND	
CoQ9	ND	ND	59	ND	ND	358	ND	
Coniferylaldehyde	ND	107	ND	ND	ND	ND	ND	
Cortisol	ND	43	ND	ND	ND	ND	ND	
Creatine phosphate	ND	ND	ND	ND	ND	315	ND	
Cyclic adenosine diphosphate ribose	ND	479	ND	ND	ND	ND	ND	
Cys-Gly	ND	ND	ND	ND	ND	ND		543
Cystathionine	ND	265	ND	ND	ND	ND		171
Cytarabine	ND	ND	ND	ND	ND	ND		541
Cytidine	ND	211	257	207	ND	ND	ND	
Cytidine 2',3'-cyclic monophosphoric acid	ND	208	ND	ND	ND	ND		225
Cytidine 5'-diphosphate ethanolamine	ND	353	ND	ND	ND	ND	ND	
Cytidine 5'-diphosphocholine	ND	442	ND	ND	384	771		540
Cytidine 5'-monophosphate	ND	ND	ND	ND	310	ND	ND	

Cytidine-5'-monophosphate	ND	ND	ND	ND	ND	ND	332
D-(+)-Trehalose	ND	ND	ND	ND	2	ND	459
D-Fructose 6-phosphate	ND	ND	ND	ND	120	ND	458
D-Glucosaminic acid	ND	ND	ND	ND	ND	ND	457
D-Glucose 6-phosphate	ND	ND	ND	ND	128	ND	198
D-Leucyl-L-arginine	ND	ND	ND	ND	ND	ND	456
D-Ribose 5-phosphate	ND	ND	ND	ND	293	ND	ND
DAG 18:0_18:1	ND	ND	ND	ND	193	ND	ND
DAG 18:0_22:6	ND	ND	ND	ND	161	ND	ND
DG 30:0	ND	ND	166	ND	ND	ND	ND
DG 30:0 | DG 14:0_16:0	ND	ND	ND	681	ND	298	ND
DG 30:1 | DG 14:0_16:1	ND	ND	ND	12	ND	136	ND
DG 30:2 | DG 12:0_18:2	ND	ND	ND	192	ND	428	ND
DG 32:0 | DG 16:0_16:0	ND	ND	ND	209	ND	772	ND
DG 32:1	ND	423	28	181	16	773	454
DG 32:2 | DG 14:0_18:2	ND	ND	ND	86	ND	ND	ND
DG 32:2 | DG 16:1_16:1	ND	ND	ND	ND	ND	776	ND
DG 32:3 | DG 14:1_18:2	ND	ND	ND	202	ND	413	ND
DG 33:1 | DG 15:0_18:1	ND	ND	ND	276	ND	777	ND
DG 33:2 | DG 15:0_18:2	ND	ND	ND	39	ND	778	ND
DG 34:0	ND	419	ND	ND	325	ND	ND
DG 34:0 | DG 16:0_18:0	ND	ND	ND	69	ND	ND	ND
DG 34:3 | DG 16:0_18:3	ND	ND	ND	520	ND	ND	ND
DG 34:3 | DG 16:1_18:2	ND	ND	ND	280	ND	ND	ND
DG 34:4 | DG 16:1_18:3	ND	ND	ND	184	ND	781	ND
DG 35:1 | DG 17:0_18:1	ND	ND	ND	185	ND	47	ND
DG 35:2	ND	ND	ND	ND	ND	ND	450
DG 35:2 | DG 17:0_18:2	ND	ND	ND	522	ND	ND	ND
DG 35:2 | DG 17:1_18:1	ND	ND	ND	128	ND	782	ND
DG 35:3	ND	ND	ND	ND	ND	ND	449
DG 35:3 | DG 17:1_18:2	ND	ND	ND	194	ND	783	ND
DG 36:0	ND	337	ND	ND	ND	ND	45
DG 36:1	ND	ND	75	ND	540	81	191
DG 36:1 | DG 18:0_18:1	ND	ND	ND	243	ND	ND	ND
DG 36:2 | DG 18:0_18:2	ND	ND	ND	16	ND	ND	ND
DG 36:4 | DG 16:0_20:4	ND	ND	ND	48	ND	ND	ND
DG 37:2	ND	ND	ND	151	ND	ND	ND
DG 37:3 | DG 19:1_18:2	ND	ND	ND	486	ND	790	ND
DG 37:7	ND	ND	ND	487	ND	ND	80
DG 37:8	ND	ND	ND	232	ND	ND	ND
DG 38:1 | DG 20:0_18:1	ND	ND	ND	ND	ND	52	ND
DG 38:2	ND	ND	ND	ND	ND	ND	243
DG 38:2 | DG 18:1_20:1	ND	ND	ND	122	ND	791	ND

DG 38:3 | DG 20:1_18:2	ND	ND	ND	245	ND	793	ND
DG 38:4 | DG 18:0_20:4	ND	ND	ND	488	ND	ND	ND
DG 38:4 | DG 18:2_20:2	ND	ND	ND	ND	ND	303	ND
DG 38:5 | DG 16:0_22:5	ND	ND	ND	152	ND	ND	ND
DG 38:6	ND	ND	ND	ND	306	ND	125
DG 38:6 | DG 16:0_22:6	ND	ND	ND	100	ND	53	ND
DG 38:7 | DG 16:1_22:6	ND	ND	ND	489	ND	ND	ND
DG 39:7	ND	ND	50	547	ND	ND	ND
DG 39:8	ND	ND	65	ND	ND	ND	ND
DG 39:9	ND	ND	ND	516	ND	ND	ND
DG 40:3 | DG 22:1_18:2	ND	ND	ND	ND	ND	797	ND
DG 40:5	ND	ND	ND	ND	ND	ND	182
DG 40:5 | DG 18:0_22:5	ND	ND	ND	ND	ND	118	ND
DG 40:6 | DG 18:1_22:5	ND	ND	ND	517	ND	798	ND
DG 40:7	ND	ND	ND	ND	ND	ND	336
DG 40:7 | DG 18:2_22:5	ND	ND	ND	433	ND	ND	ND
DG 40:9 | DG 18:3_22:6	ND	ND	ND	ND	ND	803	ND
DG 41:10	ND	ND	ND	2	ND	ND	ND
DG 41:6	ND	ND	ND	435	ND	ND	ND
DG 41:7	ND	ND	130	59	ND	2	ND
DG 41:8	ND	ND	259	436	ND	1	ND
DG 41:9	ND	ND	92	255	ND	804	ND
DG 42:7	ND	ND	ND	437	ND	ND	184
DG 43:10	ND	ND	4	127	ND	44	ND
DG 43:11	ND	ND	35	514	ND	ND	ND
DG 43:8	ND	ND	274	3	ND	15	ND
DG 43:9	ND	ND	80	515	ND	805	ND
DG 44:8	ND	ND	ND	485	ND	ND	ND
DG 44:9	ND	ND	167	ND	ND	809	ND
DG 45:12	ND	ND	ND	218	ND	ND	ND
DG 45:8	ND	ND	ND	174	ND	ND	ND
DG 47:7	ND	ND	ND	375	ND	ND	ND
DL-2-Aminocaprylic acid	ND	ND	ND	ND	ND	ND	335
DL-Arginine	ND	ND	ND	ND	ND	ND	334
DL-Lanthionine	ND	ND	ND	ND	ND	ND	91
DL-Tryptophan, methyl ester	ND	ND	ND	ND	ND	ND	297
DMPE 36:1	ND	ND	ND	ND	ND	ND	333
DMPE 40:6	ND	ND	ND	ND	ND	ND	942
Decanoyl-carnitine	ND	81	ND	ND	ND	ND	ND
Diethyl (carboxymethylamino)methylenemalonate	ND	ND	ND	376	ND	ND	ND
Diethylcarbamazine	ND	7	ND	ND	ND	ND	ND
Dimethyl sulfoxide	ND	ND	ND	ND	ND	150	ND
Dimethyllysine	ND	ND	ND	ND	522	ND	ND

Dimetilan	ND	ND	ND	ND	ND	811	ND
Docosahexaenoylglycine	ND	522	ND	ND	ND	ND	ND
Dodeca-2(E),4(E)-dienoic acid	ND	ND	207	ND	ND	ND	ND
Dodecanoic acid, 12-[[(cyclohexylamino)carbonyl]amino]-	ND	ND	341	ND	ND	ND	ND
Ecgonine	ND	ND	ND	ND	324	ND	ND
Ethanolamine	ND	ND	325	378	ND	293	ND
Ethyl 2-acetylpentanoate	ND	ND	ND	154	ND	ND	ND
Ethyl 4-amino-1-piperidinecarboxylate	ND	387	ND	ND	ND	ND	ND
Ethyl 4-oxo-3-piperidinecarboxylate	ND	ND	ND	47	ND	ND	ND
FA 10:0	ND	91	ND	ND	78	ND	37
FA 11:0	ND	345	ND	ND	202	ND	939
FA 12:0	ND	88	ND	ND	17	ND	938
FA 13:0	ND	234	ND	ND	8	ND	163
FA 14:0	ND	ND	ND	ND	339	ND	937
FA 14:0 (myristic acid)	ND	ND	ND	452	ND	ND	ND
FA 14:1 (physeteric acid)	ND	ND	ND	159	ND	ND	ND
FA 15:4	ND	ND	ND	ND	ND	288	ND
FA 16:0	ND	151	ND	275	538	ND	934
FA 16:1 (palmitoleic acid)	ND	ND	ND	453	ND	812	ND
FA 16:3	ND	ND	ND	430	ND	817	ND
FA 16:4	ND	ND	ND	111	ND	818	ND
FA 17:0 (margaric acid)	ND	ND	ND	431	ND	ND	ND
FA 17:2	ND	ND	ND	329	ND	836	ND
FA 18:0	ND	145	ND	ND	319	ND	932
FA 18:1 (oleic acid)	ND	ND	ND	ND	ND	848	ND
FA 18:1; (oleic acid)	ND	ND	ND	330	ND	ND	ND
FA 18:2 (linoleic acid)	ND	ND	ND	41	ND	866	ND
FA 18:3 (linolenic acid)	ND	ND	ND	331	ND	867	ND
FA 18:5	ND	ND	ND	ND	ND	876	ND
FA 19:2	ND	ND	ND	398	ND	879	ND
FA 20:0	ND	316	ND	ND	245	ND	ND
FA 20:1 (eicosenoic acid)	ND	ND	ND	370	ND	885	ND
FA 20:2 (eicosadienoic acid)	ND	ND	ND	371	ND	886	ND
FA 20:3 (homo-gamma-linolenic acid)	ND	ND	ND	372	ND	887	ND
FA 20:4 (arachidonic acid)	ND	ND	ND	189	ND	888	ND
FA 20:5 (eicosapentaenoic acid)	ND	ND	ND	373	ND	899	ND
FA 21:1	ND	ND	ND	264	ND	406	ND
FA 22:1 (erucic acid)	ND	ND	ND	325	ND	ND	ND
FA 22:2 (docosadienoic acid)	ND	ND	ND	326	ND	903	ND
FA 22:3	ND	ND	ND	327	ND	907	ND
FA 22:4	ND	ND	ND	240	ND	912	ND
FA 22:6 (docosahexaenoic acid)	ND	ND	ND	249	ND	ND	ND
FA 23:1	ND	ND	ND	186	ND	913	ND

FA 24:1 (nervonic acid)	ND	ND	ND	268	ND	922	ND
FA 24:2	ND	ND	ND	125	ND	93	ND
FA 24:4	ND	ND	ND	123	ND	ND	ND
FA 25:1	ND	ND	ND	355	ND	ND	ND
FA 26:1	ND	ND	ND	135	ND	ND	ND
FA 40:6	ND	ND	ND	740	ND	ND	ND
FA 44:10	ND	ND	ND	7	ND	ND	ND
FAD	ND	352	ND	ND	521	ND	671
FAHFA 34:1;O|FAHFA 16:0/18:1;O	ND	ND	ND	725	ND	ND	ND
GM3 36:1;2O	ND	ND	ND	ND	ND	925	ND
Galacto-N-biose	ND	ND	ND	ND	72	ND	945
Gentiobiose	ND	ND	ND	ND	ND	ND	943
Geranic acid	ND	39	ND	ND	ND	ND	ND
GlcCer d34:1	ND	ND	ND	ND	ND	ND	319
GlcCer d40:1	ND	400	ND	726	107	ND	318
GlcCer d41:1	ND	ND	ND	ND	165	ND	ND
GlcCer d42:1	ND	298	ND	ND	241	ND	307
GlcCer d42:2	ND	415	ND	343	364	ND	277
Gln-Arg	ND	ND	ND	ND	ND	386	ND
Gln-Gln	ND	ND	ND	ND	ND	149	ND
Gln-Glu	ND	527	ND	ND	ND	176	ND
Gln-Lys	ND	ND	ND	ND	74	926	ND
Gln-Thr	ND	500	ND	ND	484	929	316
Glu-Ala	ND	454	ND	ND	ND	ND	ND
Glu-Asp	ND	257	ND	ND	ND	ND	ND
Glu-Gln	ND	385	ND	ND	ND	ND	13
Glu-Gln.1	ND	210	ND	ND	ND	ND	ND
Glu-Glu-Arg	ND	175	ND	ND	ND	ND	ND
Glu-Gly-Arg	ND	ND	ND	344	ND	ND	259
Glu-His	ND	520	ND	ND	ND	ND	315
Glu-Phe	ND	ND	ND	ND	ND	ND	314
Glu-Pro-Arg	ND	ND	ND	ND	ND	931	ND
Glu-Ser	ND	ND	ND	ND	ND	327	ND
Glucose-1-phosphate	ND	ND	ND	ND	218	ND	312
Glucose-6-phosphate	ND	ND	ND	315	ND	ND	ND
Glutarylcarnitine	ND	78	ND	ND	ND	ND	ND
Glutathione (oxidized)	ND	570	ND	ND	139	379	928
Glutathionesulfonic acid	ND	333	ND	ND	ND	ND	223
Gly-Arg	ND	451	ND	ND	166	ND	331
Gly-Gly-Gly	ND	437	ND	ND	ND	ND	330
Gly-His	ND	ND	ND	ND	392	ND	329
Gly-Lys	ND	ND	ND	ND	211	932	328
Gly-Pro-Lys	ND	ND	ND	318	ND	ND	ND

Gly-val	ND	ND	ND	ND	39	ND	326
Glycerol 1-myristate	ND	355	ND	ND	ND	ND	ND
Glycine	ND	ND	381	320	ND	ND	ND
Goralatide	ND	409	ND	ND	ND	ND	ND
Guanidinosuccinic acid	ND	ND	ND	ND	229	934	240
Guanosine	ND	357	ND	ND	279	ND	322
Guanosine 5'-monophosphate	ND	285	ND	ND	173	ND	321
HMBA	ND	468	ND	ND	ND	ND	ND
Heptadecasphing-4-enine	ND	ND	262	ND	ND	399	ND
Heptadecasphinganine	ND	8	ND	ND	ND	ND	ND
Heptanedioic acid, 1-(2-cyclopentylidenehydrazide)	ND	ND	297	ND	ND	ND	ND
Hex2Cer 40:1	ND	ND	ND	ND	ND	ND	320
Hex2Cer 40:2	ND	ND	ND	ND	ND	ND	210
Hex2Cer 41:1	ND	ND	ND	ND	ND	ND	354
Hex2Cer 41:2	ND	ND	ND	ND	ND	ND	353
Hex2Cer 42:1	ND	ND	ND	ND	ND	ND	352
Hex2Cer 42:3	ND	ND	ND	ND	ND	ND	351
Hex2Cer 43:2	ND	ND	ND	ND	ND	ND	303
Hex3Cer 34:1	ND	ND	ND	ND	ND	ND	350
Hex3Cer 34:2	ND	ND	ND	ND	ND	ND	349
Hex3Cer 38:1	ND	ND	ND	ND	ND	ND	348
Hex3Cer 40:1	ND	ND	ND	ND	ND	ND	347
Hex3Cer 40:2	ND	ND	ND	ND	ND	ND	346
Hex3Cer 41:1	ND	ND	ND	ND	ND	ND	345
Hex3Cer 41:2	ND	ND	ND	ND	ND	ND	344
Hex3Cer 42:1	ND	ND	ND	ND	ND	ND	343
Hex3Cer 42:2	ND	ND	ND	ND	ND	ND	342
Hex3Cer 42:3	ND	ND	ND	ND	ND	ND	341
HexCer 34:2	ND	ND	ND	ND	ND	ND	340
HexCer 36:1;2O	ND	ND	ND	321	ND	ND	ND
HexCer 36:1;2O|HexCer 18:1;2O/18:0	ND	ND	ND	ND	ND	936	ND
HexCer 38:1	ND	ND	ND	ND	ND	ND	188
HexCer 38:1;2O	ND	ND	ND	322	ND	ND	ND
HexCer 38:1;2O|HexCer 18:1;2O/20:0	ND	ND	ND	ND	ND	938	ND
HexCer 38:1;3O	ND	ND	ND	ND	ND	939	ND
HexCer 38:1;3O|HexCer 16:1;2O/22:0;O	ND	ND	ND	323	ND	ND	ND
HexCer 38:1;3O|HexCer 18:1;2O/20:0;O	ND	ND	ND	ND	ND	940	ND
HexCer 40:0	ND	ND	ND	ND	ND	ND	66
HexCer 40:0;2O	ND	ND	ND	324	ND	941	ND
HexCer 40:0;2O|HexCer 18:0;2O/22:0	ND	ND	ND	ND	ND	942	ND
HexCer 40:0;3O	ND	ND	ND	ND	ND	947	ND
HexCer 40:1;3O	ND	ND	ND	ND	ND	953	ND
HexCer 40:1;3O|HexCer 16:1;2O/24:0;O	ND	ND	ND	288	ND	ND	ND

HexCer 40:1;3O|HexCer 18:1;2O/22:0;O	ND	ND	ND	ND	ND	959	ND
HexCer 40:2;2O|HexCer 18:1;2O/22:1	ND	ND	ND	ND	ND	960	ND
HexCer 41:1;2O	ND	ND	ND	289	ND	ND	ND
HexCer 41:1;2O|HexCer 18:1;2O/23:0	ND	ND	ND	ND	ND	962	ND
HexCer 41:1;3O	ND	ND	ND	ND	ND	963	ND
HexCer 41:1;3O|HexCer 16:1;2O/25:0;O	ND	ND	ND	290	ND	ND	ND
HexCer 41:1;3O|HexCer 18:1;2O/23:0;O	ND	ND	ND	ND	ND	965	ND
HexCer 41:2	ND	ND	ND	ND	ND	ND	137
HexCer 41:3	ND	ND	ND	ND	ND	ND	366
HexCer 42:0	ND	ND	ND	ND	ND	ND	275
HexCer 42:0;2O	ND	ND	ND	291	ND	ND	ND
HexCer 42:0;2O|HexCer 18:0;2O/24:0	ND	ND	ND	ND	ND	966	ND
HexCer 42:0;3O	ND	ND	ND	ND	ND	967	ND
HexCer 42:1;2O|HexCer 18:1;2O/24:0	ND	ND	ND	292	ND	968	ND
HexCer 42:1;3O	ND	ND	ND	ND	ND	969	ND
HexCer 42:1;3O|HexCer 16:1;2O/26:0;O	ND	ND	ND	293	ND	ND	ND
HexCer 42:1;3O|HexCer 18:1;2O/24:0;O	ND	ND	ND	ND	ND	970	ND
HexCer 42:2;3O	ND	ND	ND	ND	ND	973	ND
HexCer 42:2;3O|HexCer 16:1;2O/26:1;O	ND	ND	ND	295	ND	ND	ND
HexCer 42:2;3O|HexCer 18:1;2O/24:1;O	ND	ND	ND	ND	ND	974	ND
HexCer 42:3;2O	ND	ND	ND	296	ND	975	ND
HexCer 42:3;2O|HexCer 18:1;2O/24:2	ND	ND	ND	ND	ND	976	ND
Hexadecyltrimethylammonium	ND	ND	272	297	ND	ND	ND
Hexamethylcyclotrisiloxane	ND	268	ND	ND	ND	ND	ND
His-Ala	ND	179	ND	298	ND	978	363
His-Asn	ND	ND	ND	ND	ND	ND	362
His-Asp	ND	432	ND	ND	ND	ND	361
His-Gln	ND	511	ND	ND	70	979	360
His-Glu	ND	531	ND	ND	ND	ND	359
His-Gly	ND	ND	ND	ND	ND	330	358
His-Leu	ND	129	ND	ND	ND	ND	ND
His-Met	ND	ND	ND	ND	ND	216	ND
His-Ser	ND	493	ND	ND	ND	ND	356
His-Thr	ND	ND	ND	ND	257	ND	355
His-Tyr	ND	5	ND	ND	ND	377	ND
His-Val	ND	484	ND	299	ND	980	380
Histamine	ND	34	367	300	ND	69	ND
Hydroxylysine	ND	ND	ND	ND	ND	ND	254
Ile-Arg	ND	394	ND	308	ND	ND	ND
Ile-Glu	ND	ND	ND	ND	ND	984	ND
Ile-Gly-Lys	ND	ND	ND	ND	ND	ND	377
Ile-His	ND	ND	ND	ND	413	49	376
Ile-Ile	ND	ND	ND	ND	414	26	ND

Ile-Met	ND	ND	ND	ND	ND	38	ND
Ile-Pro	ND	ND	ND	ND	ND	ND	374
Ile-Pro-Arg	ND	498	ND	ND	ND	ND	ND
Ile-Pro-Ile	ND	ND	ND	ND	ND	54	ND
Ile-Ser	ND	ND	ND	ND	ND	986	ND
Indarubicin	ND	ND	ND	91	ND	ND	ND
Inosine 5'-monophosphate	ND	ND	ND	ND	ND	987	ND
Inosine-5'-monophosphate	ND	101	ND	ND	19	ND	372
Isoleucine	ND	ND	383	310	ND	ND	ND
Isoproturon	ND	ND	266	ND	ND	ND	ND
Isouron	ND	71	ND	ND	ND	ND	ND
Isovaleryl-L-carnitine	ND	ND	ND	ND	285	ND	43
Ketamine	ND	ND	ND	34	ND	ND	ND
L-.beta.-Homoglutamine	ND	ND	ND	ND	ND	ND	371
L-.beta.-Homolysine	ND	ND	ND	ND	ND	ND	370
L-.beta.-Homomethionine	ND	ND	ND	ND	ND	ND	369
L-.gamma.-Glutamyl-L-glutamic acid	ND	ND	ND	ND	ND	ND	368
L-Arginine, methyl ester	ND	ND	ND	ND	ND	ND	367
L-Cysteine S-sulfate	ND	ND	ND	ND	ND	ND	392
L-Cysteine-glutathione disulfide	ND	ND	ND	ND	323	ND	272
L-Glutamic acid, dimethyl ester	ND	ND	ND	ND	ND	ND	391
L-Homocitrulline	ND	ND	ND	ND	ND	ND	389
L-Leucine, methyl ester	ND	ND	242	ND	ND	ND	ND
L-Phenylalanine, methyl ester	ND	ND	ND	311	ND	ND	ND
L-Proline	ND	ND	ND	312	ND	ND	ND
L-Propionylcarnitine	ND	330	ND	ND	ND	ND	388
L-Saccharopine	ND	ND	ND	ND	284	ND	387
L-Tryptophan	ND	ND	ND	313	ND	ND	386
L-Valinamide	ND	ND	361	177	ND	ND	ND
L-gamma-Glutamyl-L-glutamic acid	ND	ND	ND	ND	280	ND	ND
LPC 17:1	ND	ND	ND	ND	382	ND	385
LPC 20:0	ND	ND	ND	ND	553	ND	384
LPC 20:1	ND	ND	ND	ND	294	ND	383
LPC 20:2	ND	ND	ND	ND	ND	ND	382
LPC 22:6	ND	ND	ND	ND	554	ND	ND
LPC 14:0	ND	507	ND	ND	288	990	294
LPC 15:0	ND	ND	ND	ND	555	348	148
LPC 16:0	ND	244	ND	314	557	991	381
LPC 16:1	ND	284	ND	285	343	993	193
LPC 17:0	ND	ND	ND	ND	373	994	ND
LPC 17:1	ND	ND	ND	ND	ND	995	ND
LPC 18:2	ND	362	ND	220	544	998	406
LPC 18:3	ND	ND	ND	ND	400	1000	405

LPC 19:0	ND	ND	ND	ND	ND	86	ND
LPC 20:0	ND	446	ND	ND	ND	271	ND
LPC 20:1	ND	413	ND	ND	ND	ND	ND
LPC 20:2	ND	334	ND	ND	290	ND	ND
LPC 20:3	ND	186	ND	ND	417	1001	404
LPC 20:4	ND	260	ND	106	571	1005	ND
LPC 20:4.1	ND	ND	ND	ND	ND	1007	ND
LPC 20:5	ND	216	ND	ND	ND	270	403
LPC 22:4	ND	144	ND	ND	572	ND	ND
LPC 22:5	ND	ND	ND	ND	224	401	402
LPC 22:5.1	ND	ND	ND	ND	ND	389	ND
LPC 22:6	ND	312	ND	246	ND	1008	ND
LPC 22:6.1	ND	ND	ND	ND	ND	1011	ND
LPC 24:0	ND	ND	ND	ND	ND	355	ND
LPE 16:0.1	ND	ND	ND	ND	315	1021	ND
LPE 22:5	ND	ND	ND	ND	ND	65	ND
LPE 22:6.1	ND	ND	ND	ND	ND	1034	ND
LPE O-18:1	ND	ND	ND	302	ND	1038	ND
LPG 16:0	ND	ND	ND	ND	ND	1042	ND
LPS 18:0	ND	ND	ND	305	ND	1051	ND
LPS 22:6	ND	ND	ND	ND	ND	1052	ND
Lactosylceramide d42:2	ND	ND	ND	ND	ND	ND	396
Lactoylglutathione	ND	ND	ND	ND	ND	1053	ND
Leu-Ala	ND	ND	ND	ND	ND	ND	395
Leu-Arg	ND	ND	ND	ND	141	1054	394
Leu-Lys	ND	ND	ND	ND	103	1056	393
Leu-Pro	ND	ND	ND	ND	ND	ND	421
Leu-Pro-Arg	ND	568	ND	ND	ND	ND	ND
Leu-Ser	ND	ND	ND	ND	ND	214	ND
Leu-Val	ND	283	ND	ND	ND	ND	ND
Leucine	ND	ND	384	306	574	ND	ND
Linoleoylcarnitine	ND	ND	ND	ND	109	ND	ND
Lumichrome	ND	ND	ND	ND	ND	ND	29
Lys-Asn	ND	532	ND	ND	ND	ND	419
Lys-Gln	ND	ND	ND	ND	ND	ND	418
Lys-Glu	ND	564	ND	ND	ND	1057	417
Lys-Gly	ND	529	ND	ND	ND	ND	ND
Lys-Phe	ND	109	ND	ND	ND	ND	ND
Lys-Pro	ND	ND	ND	ND	ND	297	416
Lys-Thr	ND	359	ND	ND	ND	ND	ND
Lys-Tyr	ND	108	ND	ND	ND	ND	ND
Lys-Val	ND	27	ND	347	ND	1059	ND
Lysine	ND	3	393	348	169	113	415

Malonyl-L-carnitine_Malonyl-L-carnitine	ND	ND	ND	ND	189	ND	ND
Malonyl-carnitine	ND	ND	ND	ND	ND	1060	ND
Maltose	ND	ND	ND	ND	6	ND	21
Maltotetraose	ND	ND	ND	ND	ND	ND	413
Maltotriose	ND	ND	ND	ND	15	ND	412
Mannosamine	ND	ND	ND	ND	136	ND	ND
Melamine	ND	ND	ND	ND	ND	1061	ND
Memantine	ND	ND	ND	ND	ND	ND	411
Met-Arg	ND	ND	ND	ND	ND	1062	ND
Met-Phe	ND	ND	ND	ND	ND	359	ND
Metanephrine	ND	ND	ND	ND	ND	ND	295
Metformin	ND	ND	201	281	ND	ND	ND
Methacholine cation	ND	ND	ND	ND	ND	ND	410
Methadone	ND	306	ND	ND	ND	ND	ND
Methionine	ND	562	394	349	402	ND	409
Methioninesulfoxide A	ND	ND	316	ND	ND	ND	ND
Methyl 1-piperazinecarboxylate	ND	526	ND	ND	ND	ND	ND
Methyl DL-pyroglutamate	ND	ND	ND	ND	ND	ND	435
Methyl jasmonate	ND	276	ND	20	ND	ND	ND
Methyl-histidine_b	ND	ND	ND	ND	ND	336	ND
Methylecgonine	ND	ND	ND	ND	ND	ND	434
Methylpropionic acid	ND	ND	142	173	ND	ND	ND
Metoprolol acid	ND	513	ND	ND	ND	ND	ND
Miglitol	ND	ND	120	ND	ND	ND	ND
Muramic acid	ND	ND	ND	ND	ND	ND	433
Muscarine	ND	ND	ND	ND	ND	131	ND
Myricetin	ND	ND	ND	ND	146	ND	ND
Myristamidopropyl dimethylamine	ND	ND	ND	ND	250	ND	ND
Myristoyl ethanolamide	ND	356	ND	ND	ND	ND	ND
Myristoyl-carnitine	ND	ND	ND	ND	ND	1064	ND
N,N'-Diacetylcystine	ND	561	ND	ND	ND	ND	ND
N,N-Dibutyl-N'-(3-chloro-2-methylphenyl)urea	ND	ND	ND	143	ND	ND	ND
N,N-Diethyl-2-aminoethanol	ND	15	ND	ND	ND	ND	ND
N-(1-Amino-3,3-dimethyl-1-oxobutan-2-yl)-1-pentyl-1H-indole-3-carboxamide	ND	ND	ND	62	ND	ND	ND
N-(15Z-Tetracosenoyl)-1-.beta.-galactosylsphing-4-enine	ND	427	ND	ND	ND	ND	ND
N-(2'-Oxobi(cyclohex)yl)acetamide	ND	ND	44	ND	ND	ND	ND
N-(2-Chlorobenzyl)-2-propanamine	ND	ND	ND	ND	137	ND	ND
N-(3-(Aminomethyl)benzyl)acetamidine	ND	ND	ND	165	ND	ND	ND
N-(3-Methoxypropyl)-9H-purin-6-amine	ND	ND	ND	352	ND	ND	ND
N-(5-Amino-1,3,4-thiadiazol-2-yl)methanesulfonamide	ND	ND	ND	213	ND	ND	ND
N-(Octadecanoyl)sphing-4-enine-1-phosphocholine	ND	ND	ND	ND	ND	1065	ND
N-(Piperidin-4-yl)methanesulfonamide	ND	ND	215	ND	ND	ND	ND
N-(tert-Butyl)-4-phenylbutanamide	ND	ND	192	ND	ND	ND	ND

N-.alpha.-(tert-Butoxycarbonyl)-L-histidine	ND	ND	390	360	ND	ND	ND
N-.alpha.-Acetyl-L-arginine	ND	ND	ND	ND	ND	ND	429
N-.alpha.-Acetyl-L-ornithine	ND	ND	43	361	ND	ND	428
N-3-Hydroxydecanoyl-L-homoserine lactone	ND	ND	ND	ND	ND	ND	427
N-Acetyl-D-galactosamine	ND	ND	ND	ND	ND	ND	426
N-Acetyl-D-galactosamine-6-phosphate	ND	ND	ND	ND	ND	ND	425
N-Acetyl-D-galactosaminitol	ND	ND	ND	ND	ND	ND	424
N-Acetyl-D-glucosamine 6-phosphate	ND	ND	ND	ND	200	ND	ND
N-Acetyl-D-lactosamine	ND	ND	ND	ND	ND	ND	422
N-Acetyl-L-Prolinamide	ND	ND	ND	221	ND	ND	ND
N-Acetyl-L-carnosine	ND	322	ND	ND	ND	1066	ND
N-Acetyl-lactosamine	ND	ND	ND	ND	ND	210	ND
N-Acetyl-leucine	ND	ND	161	ND	ND	ND	ND
N-Acetyl-methionine	ND	528	ND	ND	ND	ND	ND
N-Acetylhistamine	ND	ND	ND	ND	ND	129	ND
N-Acetylneuraminic acid, 2,3-dehydro-2-deoxy-	ND	105	ND	ND	ND	ND	ND
N-Arachidonoyl-gamma-aminobutyric acid	ND	499	ND	ND	ND	ND	ND
N-Arachidonoyltaurine	ND	514	ND	ND	ND	ND	ND
N-Benzyl-N,N-dimethyl-1-hexadecanaminium	ND	ND	ND	279	ND	ND	ND
N-Carboxyethyl-.gamma.-aminobutyric acid	ND	41	ND	ND	ND	ND	ND
N-Cyclohexyl-N'-[2-(1H-imidazol-4-yl)ethyl]urea	ND	ND	ND	365	ND	ND	ND
N-Cyclohexylcyclohexanecarboxamide	ND	163	ND	ND	ND	ND	ND
N-Decyl-N,N-dimethyl-1-decanaminium	ND	ND	ND	155	ND	ND	ND
N-Desmethylvenlafaxine	ND	ND	241	ND	ND	ND	ND
N-Formylnornicotine	ND	ND	ND	ND	ND	1068	ND
N-Hydroxy-3,4-methylenedioxyamphetamine	ND	351	ND	ND	ND	ND	ND
N-Isobutyl-3-methylbutanamide	ND	ND	ND	ND	ND	402	ND
N-Methyl-asparagine	ND	ND	227	ND	ND	ND	ND
N-Methyl-histidine	ND	ND	ND	ND	ND	1069	ND
N-Methyl-serine	ND	ND	ND	ND	ND	1070	ND
N-Methylleucine	ND	ND	ND	ND	ND	ND	7
N-Methylphenylalanine	ND	ND	ND	ND	ND	ND	446
N-Nervonoyl-D-erythro-sphingosylphosphorylcholine	ND	ND	ND	ND	ND	387	ND
N-Nitroimidazolidin-2-imine	ND	ND	ND	ND	ND	ND	445
N-[2-(4-Methoxyphenyl)ethyl]-3-methyl-2-butenamide	ND	ND	358	369	ND	ND	ND
N-[3-(1H-Imidazol-1-yl)propyl]-2-(4-methylphenyl)acetamide	ND	ND	ND	250	ND	ND	ND
N-acetyl-D-galactosamine	ND	ND	ND	ND	75	ND	ND
N-acetyl-D-mannosamine	ND	ND	ND	ND	175	ND	ND
N-acetylaspartic acid	ND	126	ND	ND	ND	1071	ND
N-acetylmannosamine	ND	ND	ND	ND	ND	ND	444
N-acetylmethionine	ND	ND	ND	ND	ND	1077	ND
N-alpha-Acetyl-L-arginine	ND	ND	ND	ND	33	ND	ND
N-epsilon-dimethyl-lysine	ND	ND	ND	ND	ND	1082	ND

N-n-Butylpropionamide	ND	541	ND	ND	ND	ND	ND
N.epsilon.-Dimethyl-L-lysine	ND	ND	ND	346	ND	ND	442
N1,N12-Diethylspermine	ND	ND	ND	115	ND	ND	ND
N2-(1-Oxo-4-phenylbutyl)-L-glutamine	ND	14	ND	ND	ND	ND	ND
N2-Methylguanosine	ND	553	ND	ND	ND	ND	441
NAE 16:0	ND	ND	ND	ND	ND	429	ND
NAE 18:0	ND	ND	ND	382	ND	430	ND
NAE 18:1	ND	ND	ND	269	ND	431	ND
NAE 18:3	ND	ND	ND	ND	ND	345	ND
NAE 20:4	ND	ND	ND	ND	ND	432	ND
NAE 22:5	ND	ND	ND	233	ND	ND	ND
NAE 23:1	ND	ND	ND	ND	ND	300	ND
NG,NG-Dimethyl-L-arginine	ND	ND	ND	383	ND	ND	ND
Nicotinamide adenine dinucleotide	ND	ND	ND	ND	ND	ND	439
Nicotinamide riboside	ND	ND	ND	ND	ND	433	ND
Nicotinamide riboside cation	ND	ND	ND	ND	102	ND	438
Norcarane-7-carboxylic acid	ND	ND	ND	357	ND	ND	ND
Norvaline	ND	ND	363	ND	ND	ND	ND
Nudifloramide	ND	203	ND	ND	ND	ND	ND
O-Desmethyl-cis-tramadol	ND	ND	148	ND	ND	ND	ND
O-Desmethylvenlafaxine	ND	ND	373	ND	ND	ND	ND
O1_CE 18:3	ND	ND	ND	ND	242	ND	ND
O1_Cer d34:1	ND	ND	ND	ND	106	ND	437
O1_Cer d36:1	ND	397	ND	ND	ND	ND	ND
O1_Cer d38:1	ND	219	ND	ND	ND	ND	ND
O1_Cer d40:1	ND	221	ND	ND	135	ND	169
O1_Cer d41:1	ND	ND	ND	ND	ND	ND	475
O1_Cer d42:1	ND	267	ND	ND	60	ND	ND
O1_Cer d42:2	ND	492	ND	358	243	ND	474
O1_Ceramide d34:1	ND	ND	139	ND	ND	ND	473
O1_Ceramide d40:1	ND	ND	205	ND	ND	ND	ND
O1_Ceramide d42:2	ND	ND	243	ND	ND	ND	ND
O1_Ceramide d44:1	ND	ND	ND	216	ND	ND	ND
O1_FA 14:0	ND	ND	ND	167	ND	ND	ND
O1_FA 14:1	ND	ND	ND	ND	ND	ND	472
O1_FA 15:1	ND	ND	ND	ND	3	ND	471
O1_FA 15:4	ND	ND	ND	ND	ND	33	ND
O1_FA 16:0	ND	ND	ND	ND	ND	409	ND
O1_FA 16:1	ND	ND	ND	ND	145	ND	215
O1_FA 16:2	ND	ND	ND	13	ND	434	ND
O1_FA 18:2	ND	ND	ND	359	331	435	470
O1_FA 18:3	ND	ND	ND	97	179	436	469
O1_FA 18:4	ND	ND	ND	ND	ND	437	ND

O1_FA 20:3	ND	ND	ND	150	287	ND	ND	
O1_FA 20:4	ND	45	ND	63	ND	438		468
O1_FA 20:5	ND	ND	ND	9	ND	ND		467
O1_FA 22:6	ND	ND	ND	387	ND	439	ND	
O1_GlcCer d34:1	ND	ND	ND	ND	ND	ND		466
O1_GlcCer d40:1	ND	ND	ND	388	ND	ND		255
O1_GlcCer d41:1	ND	ND	ND	ND	216	ND	ND	
O1_GlcCer d42:1	ND	ND	ND	ND	575	ND	ND	
O1_GlcCer d42:2	ND	229	ND	ND	372	440	ND	
O1_LPC 20:1	ND	ND	ND	ND	ND	ND		465
O1_LPC 18:1	ND	ND	ND	ND	361	ND	ND	
O1_LPC 18:2	ND	ND	ND	ND	204	318	ND	
O1_LPC 20:2	ND	ND	ND	ND	311	ND	ND	
O1_LPC 20:3	ND	ND	ND	ND	266	ND	ND	
O1_PC 34:2	ND	224	ND	ND	267	ND		464
O1_PC 34:3	ND	ND	ND	ND	567	ND		463
O1_PC 36:3	ND	ND	ND	ND	366	ND	ND	
O1_PC 36:4	ND	372	ND	ND	261	ND		462
O1_PC 36:5	ND	57	ND	ND	330	441	ND	
O1_PC 36:6	ND	ND	ND	ND	ND	ND		489
O1_PC 38:4	ND	ND	ND	ND	235	ND	ND	
O1_PC 38:6	ND	ND	ND	ND	129	442	ND	
O1_PC 38:7	ND	ND	ND	ND	ND	419		488
O1_PC 40:5	ND	ND	ND	ND	181	ND	ND	
O1_PC 40:6	ND	ND	ND	ND	160	ND	ND	
O1_PC 40:7	ND	ND	ND	ND	568	ND		283
O1_PE 38:4	ND	ND	ND	ND	ND	443	ND	
O1_PE 38:4 B	ND	ND	ND	389	ND	ND	ND	
O1_PE 38:6	ND	ND	ND	ND	385	ND		487
O1_SHexCer 40:1	ND	ND	ND	390	ND	ND	ND	
O1_SHexCer 40:1;2O	ND	ND	ND	ND	ND	40	ND	
O1_SHexCer 42:1	ND	ND	ND	391	ND	ND	ND	
O1_SHexCer 42:1;2O	ND	ND	ND	ND	ND	283	ND	
O1_SHexCer 42:2;2O	ND	ND	ND	ND	ND	444	ND	
O1_SM d34:1	ND	ND	ND	119	ND	128	ND	
O1_SM d40:1	ND	ND	ND	ND	ND	ND		486
O1_SM d42:1	ND	ND	ND	ND	ND	ND		5
O1_TAG 42:1	ND	ND	ND	ND	91	ND	ND	
O1_TAG 44:1	ND	ND	ND	ND	256	ND	ND	
O1_TAG 48:4	ND	ND	ND	66	ND	ND	ND	
O1_TAG 49:1	ND	ND	ND	ND	ND	ND		485
O1_TAG 49:2	ND	ND	ND	ND	255	ND		484
O1_TAG 49:3	ND	ND	ND	ND	334	ND	ND	

O1_TAG 50:1	ND	ND	ND	ND	569	ND	483
O1_TAG 50:2	ND	ND	ND	ND	570	ND	482
O1_TAG 51:1	ND	ND	ND	ND	ND	ND	481
O1_TAG 51:2	ND	ND	ND	ND	ND	ND	480
O1_TAG 51:3	ND	ND	ND	ND	ND	ND	479
O1_TAG 52:2	ND	ND	ND	ND	182	ND	478
O1_TAG 52:3	ND	ND	ND	ND	559	ND	477
O1_TAG 52:4	ND	ND	ND	392	ND	ND	476
O1_TAG 52:5	ND	ND	ND	393	ND	ND	503
O1_TAG 53:1	ND	ND	ND	ND	ND	ND	502
O1_TAG 53:3	ND	ND	ND	ND	ND	ND	501
O1_TAG 53:4	ND	ND	ND	ND	ND	ND	500
O1_TAG 53:5	ND	ND	ND	ND	ND	ND	499
O1_TAG 54:3	ND	ND	ND	ND	271	ND	ND
O1_TAG 54:4	ND	ND	ND	394	561	ND	498
O1_TAG 54:5	ND	ND	ND	395	ND	ND	497
O1_TAG 55:1	ND	ND	ND	ND	ND	ND	496
O1_TAG 55:2	ND	ND	ND	ND	252	ND	ND
O1_TAG 55:3	ND	ND	ND	ND	ND	ND	495
O1_TAG 56:7	ND	ND	ND	ND	562	ND	ND
O1_TAG 57:2	ND	ND	ND	ND	563	ND	ND
O1_TG 41:1	ND	ND	ND	236	ND	ND	ND
O1_TG 43:2	ND	ND	ND	379	ND	ND	ND
O1_TG 43:3	ND	ND	ND	217	ND	ND	ND
O1_TG 45:4|TG 10:0_18:2_17:2;1O	ND	ND	ND	ND	ND	445	ND
O1_TG 45:5	ND	ND	ND	380	ND	ND	ND
O1_TG 50:2	ND	ND	ND	ND	ND	72	ND
O1_TG 50:3	ND	ND	ND	ND	ND	446	ND
O1_TG 50:4	ND	ND	ND	381	ND	447	ND
O1_TG 52:3	ND	ND	ND	409	ND	ND	ND
O1_TG 52:3|TG 16:0_18:1_18:2;1O	ND	ND	ND	ND	ND	180	ND
O1_TG 52:4	ND	ND	ND	410	ND	ND	ND
O1_TG 52:4|TG 16:0_18:1_18:3;1O	ND	ND	ND	ND	ND	448	ND
O1_TG 52:5	ND	ND	ND	411	ND	ND	ND
O1_TG 52:5|TG 16:0_18:2_18:3;1O	ND	ND	ND	ND	ND	449	ND
O1_TG 52:6	ND	ND	ND	412	ND	ND	ND
O1_TG 52:6|TG 16:1_18:2_18:3;1O	ND	ND	ND	ND	ND	137	ND
O1_TG 54:4	ND	ND	ND	413	ND	ND	ND
O1_TG 54:4|TG 18:1_18:2_18:1;1O	ND	ND	ND	ND	ND	237	ND
O1_TG 54:5	ND	ND	ND	11	ND	ND	ND
O1_TG 54:5|TG 18:1_18:2_18:2;1O	ND	ND	ND	ND	ND	374	ND
O1_TG 54:6	ND	ND	ND	414	ND	ND	ND
O1_TG 54:6|TG 18:1_18:2_18:3;1O	ND	ND	ND	ND	ND	450	ND

O1_free fatty acid 16:0	ND	ND	3	ND	ND	ND	ND
O1_free fatty acid 16:2	ND	ND	15	ND	ND	ND	ND
O1_free fatty acid 18:0	ND	ND	2	ND	ND	ND	ND
O1_free fatty acid 18:2	ND	ND	68	ND	ND	ND	ND
O1_free fatty acid 18:3	ND	ND	300	ND	ND	ND	ND
O1_free fatty acid 20:4	ND	ND	25	ND	ND	ND	ND
O1_free fatty acid 20:5	ND	ND	286	ND	ND	ND	ND
O1_free fatty acid 22:6	ND	ND	22	ND	ND	ND	ND
O1_phosphatidylethanolamine 38:4	ND	ND	231	ND	ND	ND	ND
O1_sphingomyelin d34:1	ND	ND	11	ND	ND	ND	ND
O1_triacylglyceride 41:1	ND	ND	30	ND	ND	ND	ND
O1_triacylglyceride 50:4	ND	ND	151	ND	ND	ND	ND
O1_triacylglyceride 52:3	ND	ND	77	ND	ND	ND	ND
O1_triacylglyceride 52:4	ND	ND	323	ND	ND	ND	ND
O1_triacylglyceride 52:5	ND	ND	302	ND	ND	ND	ND
O1_triacylglyceride 54:5	ND	ND	123	ND	ND	ND	ND
O1_triacylglyceride 54:6	ND	ND	84	ND	ND	ND	ND
O2_Cer d40:1	ND	ND	ND	44	ND	ND	ND
O2_Cer d42:1	ND	ND	ND	ND	ND	ND	305
O2_Cer d42:2	ND	ND	ND	234	ND	ND	ND
O2_Ceramide d38:1	ND	ND	281	ND	ND	ND	ND
O2_Ceramide d40:1	ND	ND	159	ND	ND	ND	ND
O2_Ceramide d42:2	ND	ND	276	ND	ND	ND	ND
O2_FA 16:1	ND	ND	ND	ND	247	ND	ND
O2_FA 18:0	ND	ND	ND	17	ND	ND	ND
O2_FA 18:1	ND	ND	ND	384	ND	ND	ND
O2_FA 18:2	ND	ND	ND	109	ND	451	494
O2_FA 18:3	ND	ND	ND	ND	340	452	ND
O2_FA 18:3 A	ND	ND	ND	53	ND	ND	ND
O2_FA 20:5	ND	ND	ND	ND	116	ND	493
O2_FA 22:5	ND	ND	ND	385	ND	ND	ND
O2_FA 22:6	ND	ND	ND	386	238	453	ND
O2_GlcCer d34:1	ND	ND	ND	ND	ND	ND	492
O2_LPC 20:2	ND	ND	ND	ND	ND	257	491
O2_LPC 18:3	ND	ND	ND	ND	234	ND	ND
O2_PC 34:1	ND	ND	ND	ND	ND	ND	300
O2_PC 35:3	ND	ND	ND	ND	ND	ND	490
O2_PC 35:4	ND	ND	ND	ND	9	ND	ND
O2_PC 36:2	ND	ND	ND	ND	ND	ND	515
O2_PC 36:4	ND	ND	ND	ND	564	454	211
O2_PC 38:4	ND	ND	ND	ND	223	ND	514
O2_PC 38:6	ND	ND	ND	ND	397	455	ND
O2_PC 40:3	ND	ND	ND	ND	ND	ND	17

O2_PC 40:6	ND	ND	ND	ND	207	ND	513
O2_PC 40:7	ND	ND	ND	ND	251	ND	512
O2_PC 40:8	ND	ND	ND	ND	ND	ND	511
O2_PC p-32:1/PC o-32:2	ND	ND	ND	ND	565	ND	ND
O2_PC p-34:1/PC o-34:2	ND	ND	ND	ND	178	ND	ND
O2_PC p34:1/PC o34:2	ND	408	ND	ND	ND	ND	ND
O2_PE 36:4	ND	ND	ND	ND	566	ND	ND
O2_PE 38:4	ND	ND	ND	ND	303	ND	ND
O2_PE 38:6	ND	ND	ND	ND	556	ND	ND
O2_PE 40:6	ND	ND	ND	ND	398	ND	ND
O2_PE o-40:5	ND	ND	ND	428	ND	ND	ND
O2_PE o-40:6	ND	ND	ND	429	ND	ND	ND
O2_PE o-40:8	ND	ND	ND	103	ND	ND	ND
O2_SM d34:1	ND	ND	ND	ND	ND	456	ND
O2_TAG 54:2	ND	ND	ND	ND	ND	ND	510
O2_TG 52:3	ND	ND	ND	399	ND	ND	ND
O2_TG 52:5	ND	ND	ND	400	ND	ND	ND
O2_TG 54:6	ND	ND	ND	82	ND	ND	ND
O2_free fatty acid 18:0	ND	ND	17	ND	ND	ND	ND
O2_free fatty acid 18:1	ND	ND	321	ND	ND	ND	ND
O2_free fatty acid 18:2	ND	ND	295	ND	ND	ND	ND
O2_free fatty acid 18:3 A	ND	ND	67	ND	ND	ND	ND
O2_free fatty acid 18:3 B	ND	ND	336	ND	ND	ND	ND
O2_free fatty acid 20:4	ND	ND	249	ND	ND	ND	ND
O2_free fatty acid 22:6	ND	ND	263	ND	ND	ND	ND
O2_phosphatidylcholine 38:5	ND	ND	280	ND	ND	ND	ND
O2_phosphatidylethanolamine 38:2	ND	ND	72	ND	ND	ND	ND
O2_triacylglyceride 50:3	ND	ND	210	ND	ND	ND	ND
O2_triacylglyceride 52:3	ND	ND	229	ND	ND	ND	ND
O2_triacylglyceride 52:4	ND	ND	202	ND	ND	ND	ND
O2_triacylglyceride 54:4	ND	ND	303	ND	ND	ND	ND
O2_triacylglyceride 54:5	ND	ND	32	ND	ND	ND	ND
O2_triacylglyceride 54:6	ND	ND	6	ND	ND	ND	ND
O3_FA 18:1	ND	ND	ND	212	ND	ND	ND
O3_FA 18:3	ND	ND	ND	ND	195	ND	64
O3_FA 20:5	ND	ND	ND	ND	199	ND	ND
O3_LPC 20:2	ND	ND	ND	ND	270	457	ND
O3_LPC 20:1	ND	ND	ND	ND	545	ND	ND
O3_LPC 20:5	ND	ND	ND	ND	123	ND	ND
O3_PE o-40:7	ND	ND	ND	401	ND	ND	ND
O3_TAG 50:4	ND	ND	ND	ND	ND	ND	304
O3_free fatty acid 18:1	ND	ND	87	ND	ND	ND	ND
O4_FA 20:4	ND	270	ND	ND	ND	ND	ND

O4_PC 38:6	ND	ND	ND	ND	341	ND	ND
Oleamide	ND	ND	ND	79	ND	ND	ND
Oleoyl-L-carnitine	ND	ND	ND	ND	246	ND	508
Oleoyl-carnitine	ND	ND	ND	ND	ND	392	ND
Ornithine	ND	262	277	402	191	229	507
PC 18:1_14:0	ND	ND	ND	ND	356	ND	ND
PC 25:0	ND	ND	ND	ND	220	ND	ND
PC 26:0	ND	332	ND	ND	ND	ND	ND
PC 28:0	ND	ND	ND	ND	157	ND	505
PC 30:0	ND	59	ND	403	13	459	504
PC 30:1 | PC 14:0_16:1	ND	ND	ND	ND	ND	460	ND
PC 31:0	ND	354	ND	404	21	461	525
PC 31:0 | PC 15:0_16:0	ND	ND	ND	ND	ND	462	ND
PC 31:1	ND	347	ND	ND	390	463	ND
PC 32:0	ND	263	ND	405	546	464	205
PC 32:1	ND	438	ND	406	550	225	524
PC 32:3	ND	ND	ND	ND	ND	282	155
PC 33:0	ND	164	ND	ND	307	9	11
PC 33:1	ND	455	ND	408	552	466	ND
PC 33:1.1	ND	ND	ND	ND	ND	467	ND
PC 33:3	ND	ND	ND	ND	ND	369	ND
PC 34:1	ND	465	ND	418	209	469	520
PC 34:2	ND	98	ND	419	532	470	3
PC 34:3	ND	431	ND	420	525	471	183
PC 34:3.1	ND	ND	ND	ND	ND	472	ND
PC 34:4	ND	439	ND	ND	393	473	519
PC 35:0	ND	ND	ND	ND	ND	68	33
PC 35:1	ND	377	ND	421	214	474	518
PC 35:2	ND	495	ND	28	526	475	517
PC 35:2.1	ND	ND	ND	ND	ND	476	ND
PC 35:3	ND	ND	ND	422	527	477	516
PC 35:4.1	ND	ND	ND	ND	ND	478	ND
PC 36:1	ND	113	ND	423	ND	480	538
PC 36:3	ND	50	ND	258	530	481	536
PC 36:3.1	ND	ND	ND	ND	ND	482	535
PC 36:4	ND	6	ND	5	547	483	534
PC 36:5	ND	259	ND	226	548	295	533
PC 36:5.1	ND	ND	ND	ND	ND	484	ND
PC 36:6	ND	189	ND	ND	533	398	532
PC 36:6 | PC 14:0_22:6	ND	ND	ND	ND	ND	485	ND
PC 36:7	ND	ND	ND	ND	ND	ND	531
PC 37:1	ND	ND	ND	ND	ND	486	ND
PC 37:3	ND	ND	ND	ND	53	294	529

PC 37:4	ND	150	ND	ND	535	487	528
PC 37:5	ND	95	ND	ND	125	488	527
PC 37:6	ND	476	ND	ND	536	489	6
PC 37:7	ND	ND	ND	ND	ND	ND	526
PC 38:1	ND	340	ND	ND	510	490	273
PC 38:3	ND	441	ND	427	511	492	563
PC 38:3.1	ND	ND	ND	ND	ND	493	ND
PC 38:4	ND	502	ND	40	395	494	63
PC 38:5	ND	445	ND	ND	512	495	562
PC 38:5.1	ND	ND	ND	ND	ND	496	ND
PC 38:7 | PC 16:1_22:6	ND	ND	ND	ND	ND	499	ND
PC 38:8	ND	ND	ND	ND	ND	500	560
PC 38:8 | PC 16:2_22:6	ND	ND	ND	ND	ND	501	ND
PC 39:4	ND	64	ND	ND	514	395	559
PC 39:5 | PC 17:0_22:5	ND	ND	ND	ND	ND	502	ND
PC 39:7	ND	ND	ND	ND	ND	ND	557
PC 39:7 | PC 17:1_22:6	ND	ND	ND	ND	ND	503	ND
PC 39:8	ND	ND	ND	ND	ND	60	ND
PC 40:0	ND	ND	ND	ND	ND	ND	556
PC 40:11	ND	ND	ND	ND	ND	ND	2
PC 40:1 | PC 22:0_18:1	ND	ND	ND	ND	ND	504	ND
PC 40:2	ND	ND	ND	ND	ND	505	ND
PC 40:4	ND	ND	ND	ND	518	506	555
PC 40:4.1	ND	ND	ND	ND	ND	507	ND
PC 40:5.1	ND	ND	ND	ND	ND	509	ND
PC 40:6	ND	4	ND	440	519	50	553
PC 40:6.1	ND	ND	ND	ND	ND	510	ND
PC 40:7	ND	305	ND	441	520	511	576
PC 40:8	ND	375	ND	120	523	14	575
PC 40:8.1	ND	ND	ND	ND	ND	512	ND
PC 40:9	ND	ND	ND	ND	ND	ND	574
PC 40:9 | PC 18:3_22:6	ND	ND	ND	ND	ND	240	ND
PC 41:1	ND	ND	ND	ND	ND	513	ND
PC 41:7	ND	ND	ND	ND	ND	515	572
PC 42:10	ND	177	ND	ND	350	516	571
PC 42:10 | PC 20:4_22:6	ND	ND	ND	ND	ND	416	ND
PC 42:11	ND	ND	ND	ND	ND	517	ND
PC 42:1 | PC 24:0_18:1	ND	ND	ND	442	ND	518	ND
PC 42:5	ND	ND	ND	ND	239	ND	ND
PC 42:6	ND	398	ND	ND	524	520	302
PC 42:8	ND	ND	ND	ND	ND	23	570
PC 42:9	ND	ND	ND	ND	ND	521	569
PC 44:10	ND	ND	ND	ND	ND	132	ND

PC 44:11	ND	ND	ND	ND	ND	ND	568
PC 44:11 | PC 22:5_22:6	ND	ND	ND	ND	ND	522	ND
PC 44:12	ND	ND	ND	ND	ND	ND	567
PC 44:12 | PC 22:6_22:6	ND	ND	ND	ND	ND	523	ND
PC 44:6	ND	ND	ND	ND	ND	ND	566
PC 44:8	ND	ND	ND	ND	ND	ND	203
PC 45:11	ND	ND	ND	ND	ND	ND	565
PC 46:12	ND	ND	ND	ND	ND	ND	250
PC 46:6	ND	ND	ND	ND	ND	ND	590
PC 46:7	ND	ND	ND	ND	ND	ND	115
PC 60:11	ND	ND	ND	ND	ND	ND	589
PC 62:12	ND	ND	ND	ND	ND	ND	588
PC 64:12	ND	ND	ND	ND	ND	ND	587
PC 64:13	ND	ND	ND	ND	ND	ND	586
PC 64:15	ND	ND	ND	ND	ND	ND	585
PC 64:17	ND	ND	ND	ND	ND	ND	584
PC 66:17	ND	ND	ND	ND	ND	ND	583
PC O-30:0	ND	ND	ND	ND	ND	524	ND
PC O-32:1	ND	ND	ND	ND	ND	526	ND
PC O-32:1.1	ND	ND	ND	ND	ND	527	ND
PC O-33:6	ND	ND	ND	ND	ND	528	ND
PC O-34:0	ND	ND	ND	ND	ND	529	ND
PC O-34:1.1	ND	ND	ND	ND	ND	531	ND
PC O-34:2.1	ND	ND	ND	ND	ND	195	ND
PC O-34:3.1	ND	ND	ND	ND	ND	534	ND
PC O-36:5.1	ND	ND	ND	ND	ND	537	ND
PC O-36:7	ND	ND	ND	ND	ND	538	ND
PC O-37:9	ND	ND	ND	ND	ND	168	ND
PC O-38:5	ND	ND	ND	ND	ND	541	ND
PC O-38:5.1	ND	ND	ND	ND	ND	542	ND
PC O-38:6.1	ND	ND	ND	ND	ND	952	ND
PC O-38:7 | PC O-16:1_22:6	ND	ND	ND	ND	ND	546	ND
PC O-39:7	ND	ND	ND	ND	ND	548	ND
PC O-40:5	ND	ND	ND	ND	ND	549	ND
PC O-40:8 | PC O-18:2_22:6	ND	ND	ND	ND	ND	84	ND
PC O-40:9	ND	ND	ND	ND	ND	554	ND
PC o-32:0	ND	ND	ND	443	ND	ND	ND
PC o-32:1	ND	ND	ND	58	ND	ND	ND
PC o-34:1	ND	ND	ND	444	ND	ND	ND
PC o-34:2	ND	ND	ND	38	ND	ND	ND
PC o-36:4	ND	ND	ND	188	ND	ND	ND
PC o-36:5	ND	ND	ND	204	ND	ND	ND
PC o-37:2 | PC o-19:0_18:2	ND	ND	ND	415	ND	ND	ND

PC o-38:5	ND	ND	ND	416	ND	ND	ND
PC o-38:6	ND	ND	ND	461	ND	ND	ND
PC p-36:1/PC o-36:2	ND	ND	ND	ND	500	ND	ND
PC p-42:4/PC o-42:5	ND	ND	ND	ND	367	ND	ND
PC p38:2	ND	331	ND	ND	ND	ND	ND
PC p42:3	ND	368	ND	ND	ND	ND	ND
PE 32:0 | PE 16:0_16:0	ND	ND	ND	ND	ND	172	ND
PE 32:1	ND	ND	ND	ND	ND	ND	582
PE 32:1 | PE 16:0_16:1	ND	ND	ND	ND	ND	25	ND
PE 34:0	ND	ND	ND	ND	ND	557	ND
PE 34:0.1	ND	ND	ND	ND	ND	558	ND
PE 34:1	ND	ND	ND	ND	ND	559	ND
PE 34:3	ND	ND	ND	ND	ND	ND	581
PE 34:3 | PE 16:1_18:2	ND	ND	ND	463	ND	376	ND
PE 35:1	ND	ND	ND	ND	ND	ND	580
PE 35:2	ND	ND	ND	ND	ND	ND	579
PE 35:2 | PE 17:0_18:2	ND	ND	ND	160	ND	ND	ND
PE 36:1	ND	206	ND	ND	494	563	578
PE 36:2	ND	63	ND	ND	495	8	577
PE 36:2.1	ND	ND	ND	ND	ND	16	ND
PE 36:3	ND	86	ND	ND	344	567	106
PE 36:3 | PE 18:1_18:2	ND	ND	ND	466	ND	568	ND
PE 36:4	ND	193	ND	ND	496	569	599
PE 36:4.1	ND	ND	ND	ND	ND	570	ND
PE 36:5 | PE 16:0_20:5	ND	ND	ND	256	ND	571	ND
PE 36:5 | PE 16:1_20:4	ND	ND	ND	271	ND	ND	ND
PE 36:6	ND	ND	ND	ND	ND	171	ND
PE 36:6 | PE 14:0_22:6	ND	ND	ND	ND	ND	573	ND
PE 37:4	ND	ND	ND	ND	ND	ND	158
PE 37:4 | PE 17:0_20:4	ND	ND	ND	ND	ND	574	ND
PE 38:1	ND	ND	ND	ND	ND	ND	598
PE 38:2	ND	ND	ND	ND	ND	ND	114
PE 38:3 | PE 18:0_20:3	ND	ND	ND	467	ND	575	ND
PE 38:4	ND	399	ND	ND	497	576	160
PE 38:5	ND	ND	ND	ND	ND	ND	597
PE 38:5 | PE 16:0_22:5	ND	ND	ND	ND	ND	577	ND
PE 38:5 | PE 18:0_20:5	ND	ND	ND	445	ND	ND	ND
PE 38:6	ND	247	ND	ND	277	578	596
PE 38:6.1	ND	ND	ND	ND	ND	579	ND
PE 38:7	ND	ND	ND	ND	ND	ND	595
PE 38:7 | PE 16:1_22:6	ND	ND	ND	476	ND	580	ND
PE 39:4 | PE 19:0_20:4	ND	ND	ND	ND	ND	163	ND
PE 39:6	ND	ND	ND	ND	ND	ND	594

PE 39:6 | PE 17:0_22:6	ND	ND	ND	ND	ND	582	ND
PE 39:7	ND	ND	ND	ND	ND	583	ND
PE 39:7.1	ND	ND	ND	ND	ND	584	ND
PE 40:4	ND	ND	ND	ND	ND	262	116
PE 40:4 | PE 18:0_22:4	ND	ND	ND	477	ND	ND	ND
PE 40:5	ND	ND	ND	ND	ND	586	593
PE 40:5 | PE 18:0_22:5	ND	ND	ND	478	ND	587	ND
PE 40:6	ND	161	ND	ND	498	ND	592
PE 40:7	ND	ND	ND	ND	ND	ND	591
PE 40:8	ND	ND	ND	ND	ND	ND	290
PE 40:8 | PE 18:2_22:6	ND	ND	ND	481	ND	590	ND
PE 40:9	ND	ND	ND	ND	ND	591	141
PE 41:6	ND	ND	ND	ND	ND	592	ND
PE 42:10	ND	ND	ND	ND	ND	593	624
PE 42:10 | PE 20:4_22:6	ND	ND	ND	ND	ND	594	ND
PE 42:11	ND	ND	ND	ND	ND	595	ND
PE 42:1 | PE 24:0_18:1	ND	ND	ND	482	ND	596	ND
PE 42:6	ND	ND	ND	ND	ND	597	623
PE 42:6 | PE 20:0_22:6	ND	ND	ND	ND	ND	598	ND
PE 42:7 | PE 20:1_22:6	ND	ND	ND	ND	ND	108	ND
PE 42:8	ND	ND	ND	ND	ND	ND	149
PE 42:8 | PE 20:2_22:6	ND	ND	ND	ND	ND	600	ND
PE 42:9 | PE 20:3_22:6	ND	ND	ND	ND	ND	601	ND
PE 44:10 | PE 22:4_22:6	ND	ND	ND	ND	ND	602	ND
PE 44:11	ND	ND	ND	ND	ND	ND	622
PE 44:11 | PE 22:5_22:6	ND	ND	ND	ND	ND	603	ND
PE 44:12 | PE 22:6_22:6	ND	ND	ND	ND	ND	292	ND
PE 44:7	ND	ND	ND	ND	ND	ND	256
PE 44:9 | PE 22:3_22:6	ND	ND	ND	ND	ND	309	ND
PE O-32:2 | PE O-16:1_16:1	ND	ND	ND	483	ND	ND	ND
PE O-34:1 | PE O-18:1_16:0	ND	ND	ND	ND	ND	606	ND
PE O-34:2	ND	ND	ND	ND	ND	607	ND
PE O-34:2 | PE O-16:1_18:1	ND	ND	ND	484	ND	ND	ND
PE O-34:3	ND	ND	ND	ND	ND	272	ND
PE O-35:2 | PE O-17:1_18:1	ND	ND	ND	456	ND	ND	ND
PE O-36:2	ND	ND	ND	ND	ND	609	ND
PE O-36:2 | PE O-18:1_18:1	ND	ND	ND	457	ND	ND	ND
PE O-36:3 | PE O-18:2_18:1	ND	ND	ND	458	ND	610	ND
PE O-36:4 | PE O-18:3_18:1	ND	ND	ND	459	ND	611	ND
PE O-36:5	ND	ND	ND	ND	ND	250	ND
PE O-36:6	ND	ND	ND	ND	ND	613	ND
PE O-36:6 | PE O-16:1_20:5	ND	ND	ND	460	ND	ND	ND
PE O-37:7 | PE O-15:1_22:6	ND	ND	ND	ND	ND	354	ND

PE O-38:2 | PE O-20:1_18:1	ND	ND	ND	ND	ND	615	ND
PE O-38:5 | PE O-16:0_22:5	ND	ND	ND	ND	ND	617	ND
PE O-38:5 | PE O-16:1_22:4	ND	ND	ND	30	ND	618	ND
PE O-38:6	ND	ND	ND	ND	ND	620	ND
PE O-38:7	ND	ND	ND	ND	ND	621	ND
PE O-38:7 | PE O-18:3_20:4	ND	ND	ND	26	ND	ND	ND
PE O-38:8 | PE O-16:2_22:6	ND	ND	ND	ND	ND	622	ND
PE O-39:7 | PE O-17:1_22:6	ND	ND	ND	145	ND	623	ND
PE O-40:5 | PE O-18:1_22:4	ND	ND	ND	ND	ND	625	ND
PE O-40:5 | PE O-20:1_20:4	ND	ND	ND	ND	ND	31	ND
PE O-40:6 | PE O-18:1_22:5	ND	ND	ND	498	ND	628	ND
PE O-40:7 | PE O-18:2_22:5	ND	ND	ND	469	ND	630	ND
PE O-40:8	ND	ND	ND	ND	ND	631	ND
PE O-40:8 | PE O-18:2_22:6	ND	ND	ND	470	ND	ND	ND
PE O-40:9 | PE O-18:3_22:6	ND	ND	ND	4	ND	ND	ND
PE O-41:7 | PE O-19:1_22:6	ND	ND	ND	ND	ND	632	ND
PE P-34:1	ND	ND	ND	472	ND	633	ND
PE P-34:2 | PE P-16:0_18:2	ND	ND	ND	ND	ND	634	ND
PE P-35:1 | PE P-17:0_18:1	ND	ND	ND	ND	ND	635	ND
PE P-36:1	ND	ND	ND	473	ND	636	ND
PE P-36:2	ND	ND	ND	ND	ND	637	ND
PE P-36:4	ND	ND	ND	ND	ND	638	ND
PE P-38:4	ND	ND	ND	474	ND	ND	ND
PE P-38:5	ND	ND	ND	ND	ND	639	ND
PE P-40:4	ND	ND	ND	ND	ND	351	ND
PE P-40:5	ND	ND	ND	ND	ND	642	ND
PE P-40:7 | PE P-18:1_22:6	ND	ND	ND	ND	ND	644	ND
PE P-40:8 | PE P-18:2_22:6	ND	ND	ND	ND	ND	645	ND
PE o-38:6 | PE O-18:1_20:5	ND	ND	ND	475	ND	ND	ND
PE p-34:1/PE o-34:2	ND	ND	ND	ND	213	ND	ND
PE p-38:5/PE o-38:6	ND	ND	ND	ND	502	ND	ND
PE p-40:4/PE o-40:5	ND	ND	ND	ND	501	ND	ND
PE p-40:5/PE o-40:6	ND	ND	ND	ND	391	ND	ND
PE p38:3	ND	73	ND	ND	ND	ND	ND
PE p40:7	ND	275	ND	ND	ND	ND	ND
PG 32:0 | PG 16:0_16:0	ND	ND	ND	ND	ND	289	ND
PG 32:1 | PG 16:0_16:1	ND	ND	ND	ND	ND	223	ND
PG 34:1 | PG 16:0_18:1	ND	ND	ND	506	ND	649	ND
PG 34:2 | PG 16:0_18:2	ND	ND	ND	511	ND	650	ND
PG 35:1 | PG 16:0_19:1	ND	ND	ND	ND	ND	651	ND
PG 36:1 | PG 18:0_18:1	ND	ND	ND	ND	ND	652	ND
PG 36:2 | PG 18:0_18:2	ND	ND	ND	512	ND	653	ND
PG 36:3 | PG 16:0_20:3	ND	ND	ND	ND	ND	654	ND

PG 36:3 | PG 18:1_18:2	ND	ND	ND	104	ND	ND	ND
PG 36:4 | PG 16:0_20:4	ND	ND	ND	ND	ND	655	ND
PG 36:4 | PG 18:2_18:2	ND	ND	ND	67	ND	ND	ND
PG 38:4 | PG 18:0_20:4	ND	ND	ND	ND	ND	656	ND
PG 38:5 | PG 16:0_22:5	ND	ND	ND	ND	ND	657	ND
PG 38:6 | PG 16:0_22:6	ND	ND	ND	ND	ND	658	ND
PG 40:6 | PG 18:0_22:6	ND	ND	ND	ND	ND	659	ND
PG 40:7 | PG 18:1_22:6	ND	ND	ND	ND	ND	660	ND
PG 40:8 | PG 18:2_22:6	ND	ND	ND	ND	ND	661	ND
PG 44:12 | PG 22:6_22:6	ND	ND	ND	ND	ND	64	ND
PGPC	ND	261	ND	ND	504	ND	620
PI 18:0_20:4	ND	ND	ND	ND	508	ND	ND
PI 32:0 | PI 16:0_16:0	ND	ND	ND	ND	ND	381	ND
PI 32:1	ND	ND	ND	ND	ND	285	ND
PI 34:0 | PI 16:0_18:0	ND	ND	ND	ND	ND	319	ND
PI 34:2	ND	ND	ND	ND	ND	666	ND
PI 34:2 | PI 16:0_18:2	ND	ND	ND	513	ND	ND	ND
PI 36:1	ND	ND	ND	ND	ND	667	ND
PI 36:1 | PI 18:0_18:1	ND	ND	ND	14	ND	ND	ND
PI 36:2.1	ND	ND	ND	ND	ND	669	ND
PI 36:2 | PI 18:0_18:2	ND	ND	ND	164	ND	ND	ND
PI 36:4.1	ND	ND	ND	ND	ND	672	ND
PI 36:4 | PI 16:0_20:4	ND	ND	ND	490	ND	ND	ND
PI 38:3	ND	ND	ND	ND	ND	675	618
PI 38:4	ND	ND	ND	ND	ND	676	617
PI 38:4.1	ND	ND	ND	ND	ND	677	ND
PI 38:5	ND	ND	ND	ND	ND	678	308
PI 38:5.1	ND	ND	ND	ND	ND	679	ND
PI 38:6	ND	ND	ND	ND	ND	680	178
PI 39:6 | PI 17:0_22:6	ND	ND	ND	ND	ND	683	ND
PI 40:4 | PI 18:0_22:4	ND	ND	ND	ND	ND	684	ND
PI 40:6.1	ND	ND	ND	ND	ND	687	ND
PI 40:7	ND	ND	ND	ND	ND	ND	269
PI 40:7 | PI 18:1_22:6	ND	ND	ND	ND	ND	688	ND
PI 40:8 | PI 18:2_22:6	ND	ND	ND	ND	ND	689	ND
POVPC	ND	344	ND	ND	422	ND	268
PS 18:0_18:2	ND	ND	ND	ND	377	ND	ND
PS 36:1 | PS 18:0_18:1	ND	ND	ND	527	ND	690	ND
PS 36:2 | PS 18:0_18:2	ND	ND	ND	528	ND	ND	ND
PS 36:2 | PS 18:1_18:1	ND	ND	ND	ND	ND	691	ND
PS 38:4 | PS 18:0_20:4	ND	ND	ND	529	ND	692	ND
PS 38:5 | PS 18:1_20:4	ND	ND	ND	530	ND	ND	ND
PS 38:6	ND	ND	ND	ND	ND	144	ND

PS 38:6 | PS 16:0_22:6	ND	ND	ND	ND	ND	694	ND
PS 39:6 | PS 17:0_22:6	ND	ND	ND	ND	ND	695	ND
PS 40:1 | PS 22:0_18:1	ND	ND	ND	ND	ND	696	ND
PS 40:5 | PS 18:0_22:5	ND	ND	ND	531	ND	697	ND
PS 40:6 | PS 18:0_22:6	ND	ND	ND	196	ND	698	ND
PS 40:7 | PS 18:1_22:6	ND	ND	ND	ND	ND	699	ND
PS 40:8 | PS 18:2_22:6	ND	ND	ND	ND	ND	700	ND
PS 42:1 | PS 24:0_18:1	ND	ND	ND	ND	ND	701	ND
PS 44:11	ND	ND	ND	ND	ND	702	ND
PS 44:11 | PS 22:5_22:6	ND	ND	ND	ND	ND	703	ND
PS 44:12 | PS 22:6_22:6	ND	ND	ND	ND	ND	178	ND
Palmitoleoyl ethanolamide	ND	453	ND	ND	ND	ND	ND
Palmitoyleicosapentaenoyl phosphatidylcholine	ND	ND	ND	ND	ND	258	ND
Pantetheine	ND	ND	ND	ND	ND	707	ND
Pantethine	ND	ND	ND	ND	ND	708	ND
Phe-Ala	ND	ND	ND	ND	ND	ND	612
Phe-Arg	ND	ND	ND	ND	ND	30	611
Phe-Lys	ND	ND	ND	ND	ND	ND	640
Phenacyltriphenylphosphonium	ND	ND	ND	231	ND	ND	ND
Phenethylamine	ND	25	ND	ND	ND	ND	ND
Phenylacetaldehyde B	ND	118	ND	ND	ND	ND	ND
Phenylalanine	ND	ND	389	500	ND	ND	ND
Phenylalanine methyl ester	ND	ND	ND	ND	371	ND	ND
Phomalone	ND	ND	ND	64	ND	ND	ND
Phosphotyrosine	ND	428	ND	ND	ND	ND	ND
Piperine	ND	51	ND	ND	ND	ND	ND
Pregabalin	ND	21	ND	ND	ND	ND	ND
Pro-Ala	ND	546	ND	ND	ND	ND	ND
Pro-Arg	ND	540	ND	ND	ND	ND	ND
Pro-Asn	ND	545	ND	ND	ND	ND	639
Pro-Asp	ND	547	ND	ND	ND	ND	ND
Pro-Glu	ND	544	ND	ND	ND	ND	ND
Pro-Leu-Lys	ND	ND	ND	ND	ND	715	ND
Pro-Lys	ND	535	ND	ND	ND	ND	637
Pro-Pro	ND	509	ND	ND	ND	ND	636
Pro-Pro_b	ND	ND	ND	ND	ND	301	ND
Pro-Ser	ND	537	ND	ND	ND	302	635
Pro-Ser-Arg	ND	ND	ND	ND	42	ND	634
Pro-Thr	ND	536	ND	ND	425	326	633
Propamocarb	ND	ND	ND	502	ND	ND	ND
Propanoic acid, 3-[[[2-[(aminoiminomethyl)amino]-4-thiazolyl]methyl]thio]-	ND	ND	372	503	ND	ND	ND
Prophosphatidylinositolonylcarnitine	ND	ND	311	ND	ND	ND	ND
Protoporphyrin IX	ND	ND	ND	ND	389	ND	ND

Pterine	ND	ND	ND	ND	328	ND	630
Purine	ND	538	ND	ND	415	ND	629
Pyridine	ND	ND	306	ND	ND	720	ND
Pyridoxine	ND	ND	ND	ND	ND	ND	626
PyroGlu-Asn-Lys	ND	ND	ND	ND	ND	ND	625
PyroGlu-Glu-Lys	ND	ND	ND	ND	ND	ND	661
PyroGlu-Gly-Arg	ND	ND	ND	ND	ND	ND	652
PyroGlu-Gly-Lys	ND	ND	ND	ND	ND	ND	651
S-Adenosyl-methionine	ND	524	ND	ND	148	723	265
SHexCer 36:1;2O	ND	ND	ND	535	ND	724	ND
SHexCer 38:1;2O	ND	ND	ND	536	ND	725	ND
SHexCer 40:1;2O	ND	ND	ND	537	ND	726	ND
SHexCer 41:1;2O	ND	ND	ND	538	ND	727	ND
SHexCer 42:0;2O	ND	ND	ND	539	ND	728	ND
SHexCer 42:1;2O	ND	ND	ND	540	ND	729	ND
SHexCer 42:2;2O	ND	ND	ND	541	ND	730	ND
SHexCer 42:3;2O	ND	ND	ND	542	ND	731	ND
SM d32:1	ND	503	ND	ND	28	352	ND
SM d32:2	ND	ND	ND	ND	ND	ND	173
SM d33:1	ND	17	ND	130	7	104	ND
SM d34:0	ND	80	ND	543	347	734	646
SM d34:1	ND	292	ND	544	450	735	645
SM d34:2	ND	452	ND	251	345	736	644
SM d35:1	ND	ND	ND	ND	ND	737	ND
SM d36:0	ND	97	ND	546	5	375	222
SM d36:1	ND	172	ND	525	283	739	19
SM d36:2	ND	142	ND	553	96	740	ND
SM d37:1	ND	29	ND	107	451	741	643
SM d38:0	ND	12	ND	ND	ND	393	ND
SM d38:1	ND	202	ND	ND	295	743	642
SM d38:1 | SM 14:1;2O/24:0	ND	ND	ND	554	ND	ND	ND
SM d38:2	ND	395	ND	163	452	276	641
SM d39:1	ND	37	ND	87	152	745	672
SM d39:2	ND	ND	ND	ND	ND	ND	105
SM d40:0	ND	440	ND	ND	396	7	270
SM d40:1	ND	199	ND	555	421	747	180
SM d40:2	ND	122	ND	556	320	748	670
SM d41:1	ND	302	ND	ND	405	34	212
SM d41:1 | SM 18:1;2O/23:0	ND	ND	ND	557	ND	ND	ND
SM d41:2	ND	ND	ND	558	406	160	669
SM d42:0	ND	ND	ND	ND	408	751	285
SM d42:1	ND	430	ND	ND	407	752	152
SM d42:1 | SM 18:1;2O/24:0	ND	ND	ND	559	ND	ND	ND

SM d42:2	ND	ND	ND	ND	412	753	14
SM d42:2|SM 18:1;2O/24:1	ND	ND	ND	560	ND	ND	ND
SM d42:3	ND	393	ND	561	363	754	249
SM d43:1	ND	429	ND	ND	409	71	666
SM d43:2	ND	542	ND	ND	410	ND	665
SM d44:2	ND	463	ND	ND	ND	ND	ND
Saccharopine	ND	194	ND	ND	ND	ND	ND
Ser-Ala	ND	ND	ND	ND	ND	ND	664
Ser-Arg	ND	ND	ND	ND	346	757	663
Ser-Asn	ND	ND	ND	ND	ND	ND	662
Ser-Gly	ND	ND	ND	ND	ND	ND	687
Ser-Lys	ND	ND	ND	ND	ND	758	685
Ser-Pro	ND	ND	ND	ND	ND	ND	684
Ser-Ser	ND	ND	ND	ND	ND	ND	683
Ser-Thr	ND	ND	ND	ND	ND	ND	682
Spermidine	ND	ND	ND	ND	ND	760	ND
Splitomicin	ND	ND	366	ND	ND	ND	ND
Stachydrine	ND	ND	ND	ND	ND	761	ND
Stearoyl-L-carnitine	ND	ND	ND	ND	221	762	680
Styrene	ND	ND	376	ND	ND	ND	ND
Sucrose	ND	ND	238	563	ND	ND	293
TAG 40:0	ND	315	ND	ND	286	ND	678
TAG 40:1	ND	349	ND	ND	262	ND	677
TAG 42:0	ND	249	ND	ND	ND	ND	676
TAG 42:1	ND	ND	ND	ND	411	ND	675
TAG 42:2	ND	ND	ND	ND	349	ND	674
TAG 42:3	ND	ND	ND	ND	ND	ND	117
TAG 44:0	ND	459	ND	ND	278	ND	673
TAG 44:1	ND	ND	ND	ND	378	ND	702
TAG 44:2	ND	ND	ND	ND	ND	ND	231
TAG 46:0	ND	201	ND	ND	233	ND	701
TAG 46:1	ND	436	ND	ND	368	ND	700
TAG 46:2	ND	214	ND	ND	231	ND	699
TAG 46:3	ND	ND	ND	ND	383	ND	698
TAG 46:4	ND	ND	ND	ND	ND	ND	697
TAG 46:5	ND	ND	ND	ND	ND	ND	696
TAG 48:0	ND	264	ND	ND	282	ND	695
TAG 48:1	ND	485	ND	ND	428	ND	260
TAG 48:2	ND	343	ND	ND	429	ND	693
TAG 48:3	ND	ND	ND	ND	430	ND	692
TAG 48:4	ND	ND	ND	ND	431	ND	691
TAG 49:0	ND	378	ND	ND	432	ND	690
TAG 49:1	ND	254	ND	ND	433	ND	689

TAG 49:2	ND		433	ND	ND		434	ND		688
TAG 49:3	ND		308	ND	ND		435	ND		717
TAG 50:0	ND	ND		ND	ND		301	ND		716
TAG 50:1	ND		488	ND	ND	ND		ND		715
TAG 50:2	ND		279	ND	ND		111	ND		714
TAG 50:3	ND		469	ND	ND		436	ND	ND	
TAG 50:4	ND		296	ND	ND		437	ND		713
TAG 50:5	ND	ND		ND	ND		438	ND		712
TAG 51:1	ND		481	ND	ND		439	ND		711
TAG 51:2	ND		127	ND	ND		357	ND		710
TAG 51:3	ND		396	ND	ND		440	ND		709
TAG 51:4	ND	ND		ND	ND		441	ND		708
TAG 51:5	ND	ND		ND	ND	ND		ND		707
TAG 52:0	ND		407	ND	ND		442	ND		206
TAG 52:1	ND		552	ND	ND		443	ND	ND	
TAG 52:2	ND		559	ND	ND	ND		ND		706
TAG 52:3	ND		491	ND	ND	ND		ND	ND	
TAG 52:4	ND		128	ND	ND		444	ND	ND	
TAG 52:5	ND	ND		ND	ND		445	ND		705
TAG 52:6	ND	ND		ND	ND		446	ND		704
TAG 53:0	ND		222	ND	ND	ND		ND	ND	
TAG 53:1	ND		471	ND	ND		447	ND		703
TAG 53:2	ND	ND		ND	ND		448	ND		732
TAG 53:3	ND	ND		ND	ND		456	ND		731
TAG 53:4	ND	ND		ND	ND		455	ND		730
TAG 53:5	ND	ND		ND	ND		454	ND		729
TAG 54:0	ND		324	ND	ND	ND		ND		728
TAG 54:1	ND		557	ND	ND		337	ND		727
TAG 54:2	ND		558	ND	ND		453	ND	ND	
TAG 54:3	ND		271	ND	ND	ND		ND	ND	
TAG 54:4	ND		286	ND	ND		459	ND		150
TAG 54:5	ND		382	ND	ND		460	ND		726
TAG 54:6	ND		448	ND	ND		467	ND		725
TAG 54:7	ND		414	ND	ND	ND		ND		724
TAG 54:8	ND	ND		ND	ND		468	ND		723
TAG 55:1	ND		505	ND	ND		457	ND		722
TAG 55:2	ND		60	ND	ND		458	ND		721
TAG 55:3	ND	ND		ND	ND		483	ND		720
TAG 56:0	ND		391	ND	ND	ND		ND	ND	
TAG 56:1	ND		518	ND	ND		462	ND		719
TAG 56:10	ND	ND		ND	ND		463	ND		718
TAG 56:2	ND		554	ND	ND		464	ND		747
TAG 56:3	ND		390	ND	ND		465	ND		746

TAG 56:4	ND	472	ND	ND	1	ND	126
TAG 56:5	ND	ND	ND	ND	466	ND	745
TAG 56:6	ND	195	ND	ND	515	ND	744
TAG 56:7	ND	28	ND	ND	516	ND	743
TAG 56:8	ND	ND	ND	ND	469	ND	742
TAG 56:9	ND	ND	ND	ND	470	ND	741
TAG 57:1	ND	227	ND	ND	ND	ND	740
TAG 57:2	ND	131	ND	ND	471	ND	739
TAG 58:0	ND	ND	ND	ND	164	ND	ND
TAG 58:1	ND	501	ND	ND	472	ND	738
TAG 58:10	ND	ND	ND	ND	473	ND	737
TAG 58:2	ND	555	ND	ND	474	ND	736
TAG 58:3	ND	470	ND	ND	475	ND	735
TAG 58:4	ND	ND	ND	ND	476	ND	734
TAG 58:5	ND	294	ND	ND	477	ND	733
TAG 58:6	ND	447	ND	ND	478	ND	761
TAG 58:8	ND	173	ND	ND	479	ND	760
TAG 58:9	ND	489	ND	ND	480	ND	759
TAG 59:2	ND	383	ND	ND	481	ND	758
TAG 59:3	ND	ND	ND	ND	ND	ND	757
TAG 60:1	ND	373	ND	ND	482	ND	52
TAG 60:11	ND	162	ND	ND	486	ND	ND
TAG 60:2	ND	478	ND	ND	485	ND	756
TAG 60:3	ND	54	ND	ND	487	ND	755
TAG 60:4	ND	ND	ND	ND	488	ND	754
TAG 60:5	ND	ND	ND	ND	ND	ND	753
TAG 60:6	ND	ND	ND	ND	491	ND	752
TAG 62:1	ND	ND	ND	ND	ND	ND	12
TAG 62:2	ND	295	ND	ND	492	ND	751
TAG 62:3	ND	205	ND	ND	ND	ND	750
TAG 62:4	ND	ND	ND	ND	376	ND	749
TAG 64:2	ND	ND	ND	ND	489	ND	748
TAG 64:3	ND	ND	ND	ND	490	ND	777
TAG 64:4	ND	110	ND	ND	499	ND	776
TG 36:0	ND	ND	ND	532	ND	ND	ND
TG 38:0 | TG 10:0_12:0_16:0	ND	ND	ND	1	ND	ND	ND
TG 38:1 | TG 10:0_10:0_18:1	ND	ND	ND	571	ND	ND	ND
TG 40:0	ND	ND	ND	ND	ND	763	ND
TG 40:0 | TG 10:0_14:0_16:0	ND	ND	ND	572	ND	ND	ND
TG 40:1	ND	ND	ND	ND	ND	12	ND
TG 41:2 | TG 12:0_14:0_15:2	ND	ND	ND	573	ND	ND	ND
TG 41:4 | TG 11:0_15:2_15:2	ND	ND	ND	574	ND	ND	ND
TG 42:0 | TG 12:0_14:0_16:0	ND	ND	ND	575	ND	ND	ND

TG 42:1	ND	ND	ND	ND	ND	140	ND
TG 42:2	ND	ND	ND	ND	ND	35	ND
TG 42:2 | TG 12:0_12:0_18:2	ND	ND	ND	576	ND	ND	ND
TG 42:3	ND	ND	ND	ND	ND	767	ND
TG 42:3 | TG 10:0_14:0_18:3	ND	ND	ND	577	ND	ND	ND
TG 43:1 | TG 9:0_16:0_18:1	ND	ND	ND	578	ND	ND	ND
TG 43:2 | TG 9:0_16:0_18:2	ND	ND	ND	ND	ND	768	ND
TG 43:3 | TG 12:0_16:1_15:2	ND	ND	ND	579	ND	769	ND
TG 44:0	ND	ND	ND	ND	ND	770	ND
TG 44:0 | TG 14:0_14:0_16:0	ND	ND	ND	548	ND	ND	ND
TG 44:1	ND	ND	ND	549	ND	75	ND
TG 44:2	ND	ND	ND	ND	ND	155	ND
TG 44:2 | TG 10:0_16:0_18:2	ND	ND	ND	550	ND	ND	ND
TG 44:3	ND	ND	ND	ND	ND	367	ND
TG 44:3 | TG 10:0_16:0_18:3	ND	ND	ND	ND	ND	ND	775
TG 44:3 | TG 8:0_18:1_18:2	ND	ND	ND	551	ND	ND	ND
TG 44:4 | TG 8:0_18:2_18:2	ND	ND	ND	552	ND	774	774
TG 45:1 | TG 12:0_15:0_18:1	ND	ND	ND	117	ND	ND	ND
TG 45:1 | TG 14:0_15:0_16:1	ND	ND	ND	ND	ND	ND	772
TG 45:2 | TG 12:0_15:0_18:2	ND	ND	ND	586	ND	ND	ND
TG 45:2 | TG 15:0_14:1_16:1	ND	ND	ND	ND	ND	ND	771
TG 45:3 | TG 9:0_18:1_18:2	ND	ND	ND	587	ND	775	ND
TG 45:4 | TG 9:0_18:2_18:2	ND	ND	ND	588	ND	331	ND
TG 46:0	ND	ND	ND	ND	ND	385	ND
TG 46:1	ND	ND	ND	ND	ND	320	ND
TG 46:1 | TG 14:0_16:0_16:1	ND	ND	ND	589	ND	ND	ND
TG 46:2	ND	ND	ND	590	ND	779	ND
TG 46:3	ND	ND	ND	591	ND	158	ND
TG 46:4	ND	ND	ND	ND	ND	275	ND
TG 46:4 | TG 10:0_18:2_18:2	ND	ND	ND	592	ND	ND	ND
TG 46:5	ND	ND	ND	ND	ND	55	ND
TG 46:5 | TG 10:0_18:2_18:3	ND	ND	ND	593	ND	ND	ND
TG 47:0 | TG 14:0_16:0_17:0	ND	ND	ND	ND	ND	ND	770
TG 47:1	ND	ND	ND	49	ND	ND	ND
TG 47:1 | TG 15:0_16:0_16:1	ND	ND	ND	ND	ND	88	769
TG 47:2	ND	ND	ND	50	ND	ND	ND
TG 47:2 | TG 14:0_15:0_18:2	ND	ND	ND	ND	ND	74	ND
TG 47:2 | TG 16:0_15:1_16:1	ND	ND	ND	ND	ND	ND	768
TG 47:3	ND	ND	ND	594	ND	ND	ND
TG 47:3 | TG 13:0_16:1_18:2	ND	ND	ND	ND	ND	786	ND
TG 47:4	ND	ND	ND	98	ND	787	ND
TG 48:0	ND	ND	ND	ND	ND	204	ND
TG 48:0 | TG 14:0_16:0_18:0	ND	ND	ND	242	ND	ND	ND

TG 48:1	ND	ND	ND	ND	ND	51	ND
TG 48:2	ND	ND	ND	ND	ND	105	ND
TG 48:2 | TG 14:0_16:1_18:1	ND	ND	ND	605	ND	ND	ND
TG 48:3	ND	ND	ND	ND	ND	145	ND
TG 48:4	ND	ND	ND	ND	ND	792	ND
TG 48:4 | TG 14:1_16:1_18:2	ND	ND	ND	607	ND	ND	ND
TG 48:5	ND	ND	ND	ND	ND	122	ND
TG 48:5 | TG 12:0_18:2_18:3	ND	ND	ND	608	ND	ND	ND
TG 48:5 | TG 16:0_16:1_16:4	ND	ND	ND	609	ND	ND	ND
TG 49:0	ND	ND	ND	ND	ND	254	ND
TG 49:0 | TG 16:0_16:0_17:0	ND	ND	ND	610	ND	ND	ND
TG 49:1	ND	ND	ND	ND	ND	350	ND
TG 49:1 | TG 15:0_16:0_18:1	ND	ND	ND	18	ND	ND	ND
TG 49:2	ND	ND	ND	ND	ND	796	ND
TG 49:3	ND	ND	ND	ND	ND	332	ND
TG 49:4 | TG 15:1_16:1_18:2	ND	ND	ND	ND	ND	124	767
TG 49:4 | TG 16:0_15:2_18:2	ND	ND	ND	611	ND	ND	ND
TG 49:5	ND	ND	ND	580	ND	ND	ND
TG 49:5 | TG 13:0_18:2_18:3	ND	ND	ND	ND	ND	799	ND
TG 50:1	ND	ND	ND	ND	ND	61	ND
TG 50:2	ND	ND	ND	ND	ND	249	ND
TG 50:2 | TG 16:0_16:1_18:1	ND	ND	ND	582	ND	ND	ND
TG 50:3	ND	ND	ND	583	ND	802	ND
TG 50:4	ND	ND	ND	ND	ND	107	ND
TG 50:5	ND	ND	ND	ND	ND	333	ND
TG 50:7 | TG 16:1_18:2_16:4	ND	ND	ND	43	ND	ND	ND
TG 51:1	ND	ND	ND	ND	ND	356	ND
TG 51:2	ND	ND	ND	ND	ND	806	ND
TG 51:3	ND	ND	ND	691	ND	807	ND
TG 51:4	ND	ND	ND	ND	ND	808	ND
TG 51:4 | TG 16:1_17:1_18:2	ND	ND	ND	596	ND	ND	ND
TG 51:5	ND	ND	ND	ND	ND	246	ND
TG 51:6 | TG 15:1_18:2_18:3	ND	ND	ND	ND	ND	368	ND
TG 51:6 | TG 15:2_18:2_18:2	ND	ND	ND	598	ND	ND	ND
TG 52:0	ND	ND	ND	ND	ND	426	ND
TG 52:1	ND	ND	ND	ND	ND	46	ND
TG 52:2	ND	ND	ND	ND	ND	338	ND
TG 52:2 | TG 16:0_18:1_18:1	ND	ND	ND	600	ND	ND	ND
TG 52:3	ND	ND	ND	ND	ND	814	ND
TG 52:3 | TG 16:0_18:1_18:2	ND	ND	ND	180	ND	ND	ND
TG 52:4	ND	ND	ND	ND	ND	815	ND
TG 52:4 | TG 16:1_18:1_18:2	ND	ND	ND	601	ND	ND	ND
TG 52:5	ND	ND	ND	602	ND	816	ND

TG 52:6	ND	ND	ND	603	ND	ND	ND
TG 52:7 | TG 16:2_18:2_18:3	ND	ND	ND	604	ND	ND	ND
TG 52:8 | TG 14:1_16:1_22:6	ND	ND	ND	ND	ND	819	ND
TG 53:0	ND	ND	ND	ND	ND	820	ND
TG 53:1	ND	ND	ND	ND	ND	821	ND
TG 53:2	ND	ND	ND	ND	ND	822	ND
TG 53:3	ND	ND	ND	ND	ND	823	ND
TG 53:4	ND	ND	ND	ND	ND	824	ND
TG 53:5	ND	ND	ND	ND	ND	825	ND
TG 53:7 | TG 15:0_16:1_22:6	ND	ND	ND	616	ND	827	ND
TG 54:0	ND	ND	ND	ND	ND	828	ND
TG 54:1	ND	ND	ND	ND	ND	196	ND
TG 54:1 | TG 16:0_20:0_18:1	ND	ND	ND	617	ND	ND	ND
TG 54:2	ND	ND	ND	ND	ND	830	ND
TG 54:3	ND	ND	ND	ND	ND	831	ND
TG 54:3 | TG 18:0_18:1_18:2	ND	ND	ND	140	ND	ND	ND
TG 54:4	ND	ND	ND	ND	ND	832	ND
TG 54:4 | TG 18:1_18:1_18:2	ND	ND	ND	619	ND	ND	ND
TG 54:5	ND	ND	ND	620	ND	833	ND
TG 54:6	ND	ND	ND	621	ND	834	ND
TG 54:7	ND	ND	ND	622	ND	835	ND
TG 54:8	ND	ND	ND	ND	ND	339	ND
TG 54:8 | TG 16:1_16:1_22:6	ND	ND	ND	623	ND	ND	ND
TG 54:8 | TG 16:1_18:2_20:5	ND	ND	ND	658	ND	ND	ND
TG 54:8 | TG 18:2_18:2_18:3	ND	ND	ND	659	ND	ND	ND
TG 54:9	ND	ND	ND	628	ND	ND	ND
TG 55:1	ND	ND	ND	ND	ND	837	ND
TG 55:1 | TG 18:0_19:0_18:1	ND	ND	ND	629	ND	ND	ND
TG 55:2	ND	ND	ND	ND	ND	838	ND
TG 55:2 | TG 18:0_18:1_19:1	ND	ND	ND	630	ND	ND	ND
TG 55:3	ND	ND	ND	ND	ND	839	ND
TG 55:4 | TG 18:1_19:1_18:2	ND	ND	ND	632	ND	840	764
TG 55:8 | TG 16:1_18:2_21:5	ND	ND	ND	636	ND	ND	792
TG 56:1	ND	ND	ND	ND	ND	845	ND
TG 56:10	ND	ND	ND	637	ND	ND	ND
TG 56:1 | TG 18:0_20:0_18:1	ND	ND	ND	638	ND	ND	ND
TG 56:2	ND	ND	ND	ND	ND	846	ND
TG 56:2 | TG 18:0_18:1_20:1	ND	ND	ND	639	ND	ND	ND
TG 56:3	ND	ND	ND	ND	ND	847	ND
TG 56:3 | TG 18:1_18:1_20:1	ND	ND	ND	640	ND	ND	ND
TG 56:4	ND	ND	ND	ND	ND	109	ND
TG 56:5	ND	ND	ND	ND	ND	849	ND
TG 56:5 | TG 18:1_18:2_20:2	ND	ND	ND	190	ND	ND	ND

TG 56:6	ND	ND	ND	ND	ND	850	ND	
TG 56:6 | TG 16:0_18:1_22:5	ND	ND	ND	644	ND	ND	ND	
TG 56:6 | TG 18:1_18:2_20:3	ND	ND	ND	157	ND	ND	ND	
TG 56:7	ND	ND	ND	ND	ND	851	ND	
TG 56:7 | TG 16:0_18:1_22:6	ND	ND	ND	645	ND	ND	ND	
TG 56:7 | TG 18:1_18:2_20:4	ND	ND	ND	646	ND	ND	ND	
TG 56:8	ND	ND	ND	ND	ND	852	ND	
TG 56:9	ND	ND	ND	ND	ND	853	ND	
TG 56:9 | TG 18:2_18:2_20:5	ND	ND	ND	649	ND	ND	ND	
TG 57:1	ND	ND	ND	ND	ND	854	ND	
TG 57:10 | TG 18:2_18:3_21:5	ND	ND	ND	650	ND	ND	ND	
TG 57:2	ND	ND	ND	ND	ND	855	ND	
TG 57:2 | TG 19:0_18:1_20:1	ND	ND	ND	652	ND	ND	ND	
TG 57:3	ND	ND	ND	653	ND	ND	ND	
TG 57:4 | TG 18:1_21:1_18:2	ND	ND	ND	654	ND	857		790
TG 57:5 | TG 21:1_18:2_18:2	ND	ND	ND	655	ND	858	ND	
TG 57:7 | TG 17:0_18:1_22:6	ND	ND	ND	656	ND	ND	ND	
TG 57:7 | TG 18:1_18:1_21:5	ND	ND	ND	657	ND	859		789
TG 57:9 | TG 18:2_18:2_21:5	ND	ND	ND	662	ND	861	ND	
TG 58:1	ND	ND	ND	ND	ND	862	ND	
TG 58:10	ND	ND	ND	ND	ND	863	ND	
TG 58:11 | TG 18:2_18:3_22:6	ND	ND	ND	664	ND	864	ND	
TG 58:11 | TG 18:2_20:4_20:5	ND	ND	ND	ND	ND	ND		788
TG 58:2	ND	ND	ND	ND	ND	403	ND	
TG 58:3	ND	ND	ND	ND	ND	394	ND	
TG 58:4	ND	ND	ND	ND	ND	868	ND	
TG 58:4 | TG 18:1_22:1_18:2	ND	ND	ND	669	ND	ND	ND	
TG 58:5	ND	ND	ND	ND	ND	138	ND	
TG 58:6	ND	ND	ND	ND	ND	870	ND	
TG 58:7 | TG 18:1_18:1_22:5	ND	ND	ND	673	ND	871	ND	
TG 58:8	ND	ND	ND	674	ND	872	ND	
TG 58:9	ND	ND	ND	702	ND	873	ND	
TG 59:2	ND	ND	ND	ND	ND	875	ND	
TG 59:2 | TG 23:0_18:1_18:1	ND	ND	ND	705	ND	ND	ND	
TG 59:3	ND	ND	ND	ND	ND	211	ND	
TG 59:3 | TG 23:0_18:1_18:2	ND	ND	ND	247	ND	ND	ND	
TG 59:4 | TG 18:1_23:1_18:2	ND	ND	ND	211	ND	877		786
TG 59:5 | TG 23:1_18:2_18:2	ND	ND	ND	52	ND	280		785
TG 59:7 | TG 19:0_18:1_22:6	ND	ND	ND	ND	ND	ND		784
TG 60:10	ND	ND	ND	684	ND	408	ND	
TG 60:10 | TG 18:2_20:3_22:5	ND	ND	ND	ND	ND	ND		783
TG 60:11	ND	ND	ND	685	ND	880	ND	
TG 60:12 | TG 16:0_22:6_22:6	ND	ND	ND	686	ND	881		782

TG 60:13 | TG 16:1_22:6_22:6	ND	ND	ND	ND	ND	882	ND
TG 60:2	ND	ND	ND	ND	ND	883	ND
TG 60:2 | TG 18:0_18:1_24:1	ND	ND	ND	688	ND	ND	ND
TG 60:3	ND	ND	ND	ND	ND	884	ND
TG 60:4	ND	ND	ND	ND	ND	264	ND
TG 60:5	ND	ND	ND	ND	ND	28	ND
TG 60:6	ND	ND	ND	ND	ND	18	ND
TG 60:6 | TG 18:1_20:1_22:4	ND	ND	ND	113	ND	ND	ND
TG 60:7	ND	ND	ND	ND	ND	59	ND
TG 60:8 | TG 18:1_18:2_24:5	ND	ND	ND	721	ND	ND	781
TG 60:9	ND	ND	ND	ND	ND	890	ND
TG 60:9 | TG 20:2_20:3_20:4	ND	ND	ND	ND	ND	ND	780
TG 61:1 | TG 18:0_25:0_18:1	ND	ND	ND	692	ND	ND	ND
TG 61:2 | TG 25:0_18:1_18:1	ND	ND	ND	83	ND	891	ND
TG 61:4 | TG 18:1_25:1_18:2	ND	ND	ND	694	ND	893	ND
TG 62:13 | TG 18:2_22:5_22:6	ND	ND	ND	697	ND	ND	247
TG 62:1 | TG 16:0_28:0_18:1	ND	ND	ND	699	ND	ND	ND
TG 62:2	ND	ND	ND	ND	ND	897	ND
TG 62:2 | TG 26:0_18:1_18:1	ND	ND	ND	700	ND	ND	ND
TG 62:3	ND	ND	ND	ND	ND	898	ND
TG 62:4	ND	ND	ND	ND	ND	182	ND
TG 62:4 | TG 18:1_26:1_18:2	ND	ND	ND	284	ND	ND	ND
TG 62:5 | TG 26:1_18:2_18:2	ND	ND	ND	708	ND	900	ND
TG 62:7 | TG 22:0_18:1_22:6	ND	ND	ND	ND	ND	ND	778
TG 63:2 | TG 16:0_29:0_18:2	ND	ND	ND	182	ND	ND	ND
TG 63:3 | TG 27:0_18:1_18:2	ND	ND	ND	74	ND	287	ND
TG 63:4 | TG 18:1_27:1_18:2	ND	ND	ND	ND	ND	902	ND
TG 64:11 | TG 18:2_18:2_28:7	ND	ND	ND	709	ND	3	ND
TG 64:1 | TG 16:0_30:0_18:1	ND	ND	ND	254	ND	ND	ND
TG 64:2 | TG 16:0_18:1_30:1	ND	ND	ND	711	ND	ND	ND
TG 64:3	ND	ND	ND	ND	ND	904	ND
TG 64:3 | TG 28:0_18:1_18:2	ND	ND	ND	712	ND	ND	ND
TG 64:4	ND	ND	ND	ND	ND	905	ND
TG 64:4 | TG 18:1_28:1_18:2	ND	ND	ND	235	ND	ND	ND
TG 64:5 | TG 28:1_18:2_18:2	ND	ND	ND	ND	ND	906	ND
TG 65:3 | TG 16:0_31:1_18:2	ND	ND	ND	ND	ND	99	ND
TG 65:3 | TG 29:0_18:1_18:2	ND	ND	ND	713	ND	ND	ND
TG 65:4 | TG 18:1_29:1_18:2	ND	ND	ND	714	ND	908	ND
TG 66:3 | TG 30:0_18:1_18:2	ND	ND	ND	715	ND	909	ND
TG 66:4 | TG 18:1_30:1_18:2	ND	ND	ND	716	ND	ND	ND
TG 66:4 | TG 30:0_18:2_18:2	ND	ND	ND	ND	ND	910	ND
TG 66:5 | TG 30:1_18:2_18:2	ND	ND	ND	717	ND	911	807
TG 67:4 | TG 18:1_31:1_18:2	ND	ND	ND	718	ND	418	ND

TG 67:5 | TG 31:1_18:2_18:2	ND	ND	ND	ND	ND	192	ND
TG 68:3;O2 | TG 16:0_18:1_18:1;O(FA 16:0)	ND	ND	ND	719	ND	ND	ND
TG 68:4;O2 | TG 16:0_18:2_16:0;O(FA 18:1)	ND	ND	ND	282	ND	ND	ND
TG 68:4 | TG 18:1_32:1_18:2	ND	ND	ND	ND	ND	914	ND
TG 68:5 | TG 32:1_18:2_18:2	ND	ND	ND	523	ND	915	806
TG 69:5 | TG 33:1_18:2_18:2	ND	ND	ND	ND	ND	916	ND
TG 70:3;O2 | TG 16:0_18:1_18:1;O(FA 18:0)	ND	ND	ND	521	ND	ND	204
TG 70:3 | TG 18:1_18:1_34:1	ND	ND	ND	ND	ND	ND	805
TG 70:4;O2 | TG 16:0_18:1_18:1;O(FA 18:1)	ND	ND	ND	519	ND	ND	ND
TG 70:5;O2 | TG 18:1_18:2_18:1;O(FA 16:0)	ND	ND	ND	745	ND	ND	ND
TG 70:5 | TG 34:1_18:2_18:2	ND	ND	ND	746	ND	917	804
TG 70:6;O2 | TG 16:0_18:2_18:1;O(FA 18:2)	ND	ND	ND	747	ND	ND	ND
TG 71:4 | TG 18:1_35:1_18:2	ND	ND	ND	ND	ND	918	ND
TG 72:5;O2 | TG 18:1_18:2_18:1;O(FA 18:0)	ND	ND	ND	748	ND	ND	ND
TG 72:5 | TG 36:1_18:2_18:2	ND	ND	ND	ND	ND	919	803
TG 72:6;O2 | TG 18:1_18:2_18:1;O(FA 18:1)	ND	ND	ND	749	ND	ND	ND
TG 72:7;O2 | TG 18:1_18:2_18:2;O(FA 18:1)	ND	ND	ND	750	ND	ND	ND
TG O-38:2	ND	ND	ND	751	ND	ND	ND
TG O-43:7 | TG O-13:1_15:3_15:3	ND	ND	ND	752	ND	ND	ND
TG O-43:8 | TG O-9:0_17:4_17:4	ND	ND	ND	753	ND	ND	ND
TG O-44:10 | TG O-8:0_18:5_18:5	ND	ND	ND	754	ND	ND	ND
TG O-50:0	ND	ND	ND	227	ND	920	ND
TG O-50:1	ND	ND	ND	22	ND	921	ND
TG O-50:2	ND	ND	ND	728	ND	94	ND
TG O-52:0	ND	ND	ND	95	ND	ND	ND
TG O-52:1	ND	ND	ND	729	ND	923	ND
TG O-52:2	ND	ND	ND	730	ND	924	ND
TG O-52:3	ND	ND	ND	162	ND	299	ND
TG O-52:4	ND	ND	ND	ND	ND	410	ND
TG O-52:4 | TG O-19:2_15:0_18:2	ND	ND	ND	731	ND	ND	ND
TG O-52:6	ND	ND	ND	ND	ND	20	ND
TG O-54:1	ND	ND	ND	732	ND	928	ND
TG O-54:2	ND	ND	ND	733	ND	312	ND
TG O-54:3	ND	ND	ND	734	ND	930	ND
TG O-54:4	ND	ND	ND	166	ND	96	ND
TG O-54:6	ND	ND	ND	518	ND	253	ND
TG O-54:7	ND	ND	ND	ND	ND	154	ND
TG O-55:1	ND	ND	ND	ND	ND	21	ND
TG O-56:2	ND	ND	ND	735	ND	935	ND
TG O-56:3	ND	ND	ND	ND	ND	307	ND
TG O-56:4	ND	ND	ND	ND	ND	937	ND
TG O-56:6	ND	ND	ND	736	ND	ND	ND
TG O-56:7	ND	ND	ND	31	ND	45	ND

TG O-56:8	ND	ND	ND	ND	ND	27	ND	
TG O-58:4	ND	ND	ND	ND	ND	373	ND	
TG O-58:6	ND	ND	ND	ND	ND	353	ND	
TG O-58:8	ND	ND	ND	ND	ND	181	ND	
Tapentadol	ND	ND	122	ND	ND	ND	ND	
Targinine	ND	ND	ND	ND	ND	ND		802
Testosterone	ND	402	ND	ND	ND	ND	ND	
Tetraethylene glycol	ND	55	206	ND	ND	ND	ND	
Tetraethylene glycol_b	ND	ND	ND	ND	ND	945	ND	
Tetramethylammonium	ND	56	ND	ND	ND	ND	ND	
Thiamine cation	ND	ND	ND	ND	ND	ND		799
Thiamine monophosphate	ND	ND	ND	ND	ND	ND		798
Thr-Ala	ND	ND	ND	ND	ND	388	ND	
Thr-Arg	ND	ND	ND	ND	14	948		797
Thr-Lys	ND	ND	ND	ND	ND	950		794
Thr-Ser	ND	ND	ND	ND	ND	951	ND	
Thr-Thr	ND	412	ND	ND	ND	ND	ND	
Threonine	ND	ND	386	545	503	ND		793
Trehalose	ND	ND	ND	ND	ND	ND		835
Triallyl cyanurate	ND	ND	251	ND	ND	ND	ND	
Tributyl phosphate	ND	169	ND	ND	ND	ND	ND	
Tridodecylmethylammonium	ND	ND	ND	222	ND	ND	ND	
Trigonelline	ND	ND	ND	ND	ND	42	ND	
Trimethylamine N-oxide	ND	ND	ND	ND	ND	305	ND	
Tripelennamine	ND	482	324	225	ND	ND	ND	
Tripropylamine	ND	563	ND	ND	ND	ND	ND	
Tris(2-butoxyethyl) phosphate	ND	11	ND	ND	ND	ND	ND	
Tryptophan	ND	551	391	569	ND	ND	ND	
Tyr-Arg	ND	ND	ND	581	108	954		821
Tyr-His	ND	ND	ND	ND	505	ND		820
Tyrosine	ND	ND	ND	626	ND	ND		819
Tyrosine butyl ester	ND	ND	190	ND	ND	ND	ND	
UDP GlcNAc	ND	ND	ND	ND	394	ND	ND	
UDP-N-acetylglucosamine	ND	288	ND	ND	ND	ND		818
UDP-glucuronic acid	ND	ND	ND	ND	95	ND		817
UMP	ND	523	ND	ND	140	ND		816
Urea	ND	ND	369	682	ND	955	ND	
Uridine 5'-monophosphate	ND	ND	ND	ND	ND	956	ND	
Uridine-5-diphosphoacetylgalactosamine	ND	ND	ND	ND	386	ND		814
Uridine-5-diphosphoacetylglucosamine	ND	548	ND	ND	ND	ND	ND	
Val-Ala	ND	ND	ND	ND	ND	ND		217
Val-Arg	ND	530	344	706	399	957		813
Val-Glu_a	ND	ND	ND	ND	ND	222	ND	

Val-His	ND	549	ND	ND	506	417	812
Val-Leu	ND	ND	ND	ND	ND	961	ND
Val-Lys	ND	ND	ND	ND	ND	ND	811
Val-Phe	ND	ND	ND	ND	ND	80	ND
Val-Ser	ND	ND	ND	ND	ND	173	ND
Val-Thr	ND	ND	ND	ND	ND	ND	810
Val-Val	ND	ND	ND	ND	ND	964	ND
Valinamide	ND	ND	ND	ND	ND	201	ND
[(4,6-Dimethyl-2-pyrimidinyl)amino]acetic acid	ND	92	ND	ND	ND	ND	ND
acetaminophen	ND	ND	ND	ND	115	ND	ND
acetylcarnitine	ND	ND	345	ND	ND	ND	ND
aconitic acid	ND	ND	ND	ND	ND	328	136
adenine	ND	ND	ND	ND	ND	197	ND
adenosine	ND	87	ND	ND	ND	ND	ND
adenosine-5-monophosphate	ND	289	ND	ND	ND	281	ND
adipic acid	ND	115	ND	ND	168	340	98
alanine	ND	153	ND	ND	162	184	261
alpha-Galactosamine-1-phosphate	ND	497	ND	742	ND	ND	ND
alpha-aminoadipic acid	ND	277	ND	ND	73	ND	165
alpha-ketoglutarate	ND	ND	ND	ND	ND	ND	93
aminomalonate	ND	209	ND	ND	174	ND	ND
aminomalonic acid	ND	ND	ND	ND	ND	286	838
arabinose	ND	ND	ND	ND	121	ND	ND
arabitol	ND	287	ND	ND	80	972	ND
arachidic acid	ND	ND	ND	ND	ND	370	124
asparagine	ND	325	ND	ND	ND	ND	ND
aspartate	ND	ND	ND	ND	ND	363	ND
aspartic acid	ND	ND	ND	ND	332	ND	ND
azelaic acid	ND	300	ND	ND	126	261	143
behenic acid	ND	ND	ND	ND	ND	325	ND
benzoic acid	ND	237	ND	ND	ND	ND	837
beta-Glycerolphosphate	ND	364	ND	ND	ND	ND	ND
beta-Glycerophosphate	ND	ND	ND	ND	12	ND	ND
beta-Homomethionine	ND	ND	ND	ND	ND	337	ND
beta-Nicotinamide adenine dinucleotide	ND	510	ND	ND	197	400	ND
beta-alanine	ND	274	ND	ND	97	123	118
beta-gentiobiose	ND	ND	ND	ND	ND	981	ND
beta-glycerolphosphate	ND	ND	ND	ND	ND	ND	189
beta.-Homoglutamine	ND	226	ND	ND	ND	ND	ND
butane-2,3-diol	ND	ND	ND	ND	ND	982	ND
butyrolactam	ND	251	ND	ND	ND	ND	836
cadaverine	ND	ND	ND	ND	ND	ND	139
caproic acid	ND	ND	ND	ND	ND	ND	133

caproic acid HMDB	ND	ND	ND	ND	ND	329	ND
caprylic acid	ND	ND	ND	ND	ND	ND	85
cellobiose	ND	ND	ND	ND	ND	207	233
cerotinic acid	ND	ND	ND	ND	ND	985	ND
citric acid	ND	326	ND	ND	244	425	869
conduritol-beta-epoxide	ND	72	ND	ND	ND	164	251
conduritol-beta-expoxide	ND	ND	ND	ND	177	ND	ND
creatinine	ND	90	ND	ND	171	988	ND
cyanoalanine	ND	ND	ND	ND	ND	989	246
cysteine	ND	139	ND	ND	114	404	282
cysteine sulfonic acid	ND	ND	ND	ND	ND	ND	860
cysteine-glycine	ND	ND	ND	ND	153	ND	ND
cytidine-5-monophosphate	ND	310	ND	ND	ND	ND	ND
dAMP	ND	341	ND	ND	ND	ND	ND
decane	ND	ND	ND	ND	ND	ND	123
dehydroascorbic acid	ND	75	ND	ND	ND	290	859
deoxypentitol	ND	ND	ND	ND	68	ND	858
diethanolamine	ND	ND	ND	ND	ND	ND	244
digalacturonic acid	ND	ND	ND	ND	ND	992	ND
diglycerol	ND	ND	ND	ND	ND	317	ND
dimethyl-PE 38:4	ND	ND	ND	ND	ND	ND	856
dimethyl-lysine	ND	ND	374	ND	ND	ND	ND
dodecanol	ND	ND	ND	ND	ND	241	ND
erythritol	ND	406	ND	ND	89	415	855
erythronic acid	ND	ND	ND	ND	43	ND	ND
erythronic acid lactone	ND	240	ND	ND	ND	ND	ND
erythronic acid lactone isomer	ND	ND	ND	ND	ND	ND	854
erythrose major	ND	ND	ND	ND	ND	ND	853
ethanolamine	ND	93	ND	ND	155	ND	852
flavin adenine	ND	ND	ND	ND	ND	ND	154
free fatty acid 14:0 (myristic acid)	ND	ND	153	ND	ND	ND	ND
free fatty acid 15:1	ND	ND	39	ND	ND	ND	ND
free fatty acid 16:0 (palmitic acid)	ND	ND	95	ND	ND	ND	ND
free fatty acid 16:1 (palmitoleic acid)	ND	ND	182	ND	ND	ND	ND
free fatty acid 16:2	ND	ND	144	ND	ND	ND	ND
free fatty acid 16:3	ND	ND	85	ND	ND	ND	ND
free fatty acid 16:4	ND	ND	138	ND	ND	ND	ND
free fatty acid 17:1	ND	ND	131	ND	ND	ND	ND
free fatty acid 17:2	ND	ND	14	ND	ND	ND	ND
free fatty acid 18:1	ND	ND	260	ND	ND	ND	ND
free fatty acid 18:2 (linoleic acid)	ND	ND	284	ND	ND	ND	ND
free fatty acid 18:3	ND	ND	164	ND	ND	ND	ND
free fatty acid 18:4	ND	ND	38	ND	ND	ND	ND

free fatty acid 19:0	ND	ND	36	ND	ND	ND	ND
free fatty acid 19:1	ND	ND	232	ND	ND	ND	ND
free fatty acid 19:2	ND	ND	89	ND	ND	ND	ND
free fatty acid 20:1 (eicosenoic acid)	ND	ND	47	ND	ND	ND	ND
free fatty acid 20:2 (eicosadienoic acid)	ND	ND	83	ND	ND	ND	ND
free fatty acid 20:3 (homo-gamma-linolenic acid)	ND	ND	90	ND	ND	ND	ND
free fatty acid 20:4 (arachidonic acid)	ND	ND	29	ND	ND	ND	ND
free fatty acid 20:5 (eicosapentaenoic acid)	ND	ND	46	ND	ND	ND	ND
free fatty acid 21:1	ND	ND	81	ND	ND	ND	ND
free fatty acid 21:5	ND	ND	103	ND	ND	ND	ND
free fatty acid 22:1 (erucic acid)	ND	ND	119	ND	ND	ND	ND
free fatty acid 22:2 (docosadienoic acid)	ND	ND	256	ND	ND	ND	ND
free fatty acid 22:3	ND	ND	197	ND	ND	ND	ND
free fatty acid 22:4	ND	ND	93	ND	ND	ND	ND
free fatty acid 22:5	ND	ND	45	ND	ND	ND	ND
free fatty acid 22:6 (docosahexaenoic acid)	ND	ND	86	ND	ND	ND	ND
free fatty acid 23:1	ND	ND	5	ND	ND	ND	ND
free fatty acid 24:1 (nervonic acid)	ND	ND	230	ND	ND	ND	ND
free fatty acid 24:2	ND	ND	186	ND	ND	ND	ND
free fatty acid 24:4	ND	ND	221	ND	ND	ND	ND
free fatty acid 24:5	ND	ND	105	ND	ND	ND	ND
free fatty acid 24:6	ND	ND	63	ND	ND	ND	ND
free fatty acid 25:1	ND	ND	71	ND	ND	ND	ND
free fatty acid 28:7	ND	ND	7	ND	ND	ND	ND
free fatty acid 34:1	ND	ND	74	ND	ND	ND	ND
free fatty acid 44:10	ND	ND	34	ND	ND	ND	ND
fructose	ND	392	ND	ND	144	233	851
fructose-1-phosphate	ND	ND	ND	ND	172	ND	884
fructose-6-phosphate	ND	ND	ND	ND	154	997	234
fucose	ND	ND	ND	ND	ND	ND	881
fumaric acid	ND	281	ND	ND	184	203	880
galactinol	ND	ND	ND	ND	228	ND	185
galactitol	ND	ND	ND	ND	ND	999	ND
galactonic acid	ND	ND	ND	ND	327	148	879
galacturonic acid	ND	ND	ND	ND	375	ND	ND
gamma-Aminobutyric Acid (GABA)	ND	ND	ND	ND	ND	ND	878
gamma-Glutamyl-cysteine	ND	ND	ND	ND	ND	ND	877
gamma-Glutamyl-glutamic acid	ND	ND	ND	ND	ND	169	ND
gamma.-L-Glutamyl-L-alanine	ND	ND	ND	ND	ND	ND	875
gamma.-Muricholic acid	ND	ND	ND	ND	ND	ND	296
gluconic acid	ND	256	ND	ND	260	ND	276
gluconic acid lactone	ND	ND	ND	ND	18	ND	ND
glucose	ND	342	ND	ND	185	1002	872

glucose-1-phosphate	ND	329	ND	ND	ND	1003	ND
glucose-6-phosphate	ND	350	ND	ND	25	1004	286
glucuronic acid	ND	ND	ND	ND	230	ND	241
glutamic acid	ND	ND	ND	ND	ND	344	870
glutamine	ND	ND	ND	ND	ND	1006	ND
glutaric acid	ND	242	ND	ND	147	273	220
glutathione	ND	ND	ND	ND	ND	422	ND
glyceraldehyde	ND	ND	ND	ND	ND	ND	894
glyceric acid	ND	467	ND	ND	167	1009	893
glycerol	ND	168	ND	ND	100	1010	179
glycerol-3-galactoside	ND	ND	ND	ND	205	362	280
glycine	ND	104	ND	ND	558	141	890
glycocyamine	ND	ND	ND	ND	ND	1014	ND
glycolic acid	ND	252	ND	ND	275	365	228
glycyl-glycine	ND	ND	ND	ND	ND	ND	889
guanidinosuccinate	ND	61	ND	ND	143	ND	ND
guanine	ND	301	ND	ND	ND	ND	ND
histidine	ND	ND	ND	ND	194	ND	ND
homocitrulline	ND	76	ND	ND	ND	ND	ND
homoserine	ND	269	ND	ND	ND	ND	ND
hydroxycarbamate	ND	ND	ND	ND	ND	1016	ND
hypotaurine	ND	327	ND	ND	ND	1017	142
hypoxanthine	ND	369	ND	ND	ND	ND	ND
indole-3-lactate	ND	ND	ND	ND	ND	ND	888
inosine	ND	148	ND	ND	ND	1018	ND
inositol-4-monophosphate	ND	319	ND	ND	187	1019	194
isoleucine	ND	79	ND	ND	560	306	887
isomaltose	ND	ND	ND	ND	263	ND	274
isooctanol	ND	ND	ND	ND	ND	252	229
isothreonic acid	ND	450	ND	ND	297	1022	885
itaconic acid	ND	49	ND	ND	ND	ND	ND
lactamide	ND	ND	ND	ND	ND	407	839
lactic acid	ND	166	ND	ND	215	291	906
lactose	ND	ND	ND	ND	40	ND	271
leucine	ND	100	ND	ND	ND	1025	904
levoglucosan	ND	280	ND	ND	212	1026	170
lignoceric acid	ND	ND	ND	ND	49	1027	ND
linoleic acid	ND	ND	ND	ND	352	1028	50
lysine	ND	ND	ND	ND	ND	1029	ND
lysophosphatidylcholine 16:0	ND	ND	244	ND	ND	ND	ND
lysophosphatidylcholine 18:0	ND	ND	23	ND	ND	ND	ND
lysophosphatidylcholine 18:1	ND	ND	109	ND	ND	ND	ND
lysophosphatidylcholine 18:2	ND	ND	125	ND	ND	ND	ND

lysophosphatidylcholine 20:4	ND	ND	217	ND	ND	ND	ND
lysophosphatidylethanolamine 16:0	ND	ND	52	ND	ND	ND	ND
lysophosphatidylethanolamine 18:0	ND	ND	333	ND	ND	ND	ND
lysophosphatidylethanolamine 18:1	ND	ND	150	ND	ND	ND	ND
lysophosphatidylethanolamine 18:2	ND	ND	124	ND	ND	ND	ND
lysophosphatidylethanolamine 20:4	ND	ND	268	ND	ND	ND	ND
lysophosphatidylethanolamine 22:6	ND	ND	76	ND	ND	ND	ND
lysophosphatidylethanolamine O-16:1	ND	ND	307	ND	ND	ND	ND
lysophosphatidylinositol 18:0	ND	ND	246	ND	ND	ND	ND
lyxitol	ND	223	ND	ND	90	ND	ND
maleic acid	ND	411	ND	ND	192	424	903
maleimide	ND	339	ND	ND	ND	ND	257
malic acid	ND	458	ND	ND	576	238	94
malonamide	ND	ND	ND	ND	ND	ND	901
maltose	ND	420	ND	ND	ND	1032	ND
maltotriose	ND	ND	ND	ND	ND	1033	ND
mannitol	ND	ND	ND	ND	ND	ND	301
mannose	ND	ND	ND	ND	338	341	899
melezitose	ND	ND	ND	ND	ND	ND	267
melibiose	ND	ND	ND	ND	110	1035	ND
methanolphosphate	ND	232	ND	ND	577	217	897
methionine sulfoxide	ND	401	ND	ND	276	ND	ND
methylmalonic acid	ND	ND	ND	ND	ND	278	ND
myo-inositol	ND	273	ND	ND	578	83	896
n-Propyl gallate	ND	ND	285	ND	ND	ND	ND
nicotinamide	ND	ND	ND	ND	317	ND	895
nicotinic acid	ND	ND	ND	ND	176	1040	226
octadecanol	ND	134	ND	ND	240	1041	201
oleamide	ND	ND	ND	ND	300	161	129
oleoylcarnitine	ND	ND	183	ND	ND	ND	ND
ornithine	ND	ND	ND	ND	ND	1043	ND
oxalic acid	ND	106	ND	ND	ND	142	874
oxoproline	ND	388	ND	ND	ND	1045	ND
p-Aminobenzoic acid	ND	ND	ND	ND	ND	ND	873
p-hydroxylphenyllactic acid	ND	ND	ND	ND	ND	1046	ND
palmitoylcarnitine	ND	ND	212	ND	ND	ND	ND
panose	ND	ND	ND	ND	ND	1047	157
pantothenic acid	ND	ND	ND	ND	ND	ND	871
parabanic acid	ND	ND	ND	ND	ND	1048	868
pentose	ND	ND	ND	ND	272	ND	ND
phenol	ND	ND	ND	ND	ND	ND	237
phenylalanine	ND	188	ND	ND	381	73	863
phenylethylamine	ND	ND	ND	ND	ND	ND	862

phosphate	ND	321	ND	ND	419	1050	242
phosphatidylcholine 30:0	ND	ND	338	ND	ND	ND	ND
phosphatidylcholine 32:0	ND	ND	301	ND	ND	ND	ND
phosphatidylcholine 34:0	ND	ND	355	ND	ND	ND	ND
phosphatidylcholine 34:2	ND	ND	278	ND	ND	ND	ND
phosphatidylcholine 35:1	ND	ND	292	ND	ND	ND	ND
phosphatidylcholine 35:2	ND	ND	108	ND	ND	ND	ND
phosphatidylcholine 36:3	ND	ND	340	ND	ND	ND	ND
phosphatidylcholine 36:4	ND	ND	308	ND	ND	ND	ND
phosphatidylcholine 36:5	ND	ND	199	ND	ND	ND	ND
phosphatidylcholine 37:2	ND	ND	21	ND	ND	ND	ND
phosphatidylcholine 38:3	ND	ND	273	ND	ND	ND	ND
phosphatidylcholine 38:5	ND	ND	225	ND	ND	ND	ND
phosphatidylcholine 38:6	ND	ND	136	ND	ND	ND	ND
phosphatidylcholine 40:5	ND	ND	290	ND	ND	ND	ND
phosphatidylcholine 40:6	ND	ND	261	ND	ND	ND	ND
phosphatidylcholine 40:7	ND	ND	319	ND	ND	ND	ND
phosphatidylcholine 40:8	ND	ND	314	ND	ND	ND	ND
phosphatidylcholine O-34:1	ND	ND	169	ND	ND	ND	ND
phosphatidylcholine O-34:2	ND	ND	121	ND	ND	ND	ND
phosphatidylcholine O-36:4	ND	ND	133	ND	ND	ND	ND
phosphatidylethanolaminentaethylene glycol	ND	ND	156	ND	ND	ND	ND
phosphatidylethanolaminerindopril	ND	ND	114	ND	ND	ND	ND
phosphoenolpyruvate	ND	ND	ND	ND	ND	ND	861
phosphogluconic acid	ND	ND	ND	ND	232	ND	845
phthalic acid	ND	196	ND	ND	ND	ND	ND
pimelic acid	ND	ND	ND	ND	ND	361	279
pinitol	ND	ND	ND	ND	170	39	218
pipecolinic acid	ND	ND	ND	ND	ND	ND	654
proline	ND	218	ND	ND	281	224	647
pseudo uridine	ND	183	ND	ND	329	1055	76
putrescine	ND	313	ND	ND	188	547	621
pyrophosphate	ND	381	ND	ND	159	1073	248
pyrrole-2-carboxylic acid	ND	ND	ND	ND	ND	1074	ND
pyruvic acid	ND	ND	ND	ND	ND	ND	616
raffinose	ND	ND	ND	ND	ND	ND	299
ribitol	ND	147	ND	ND	360	1075	614
ribonic acid	ND	137	ND	ND	358	1076	613
ribose	ND	70	ND	ND	208	316	946
ribose-5-phosphate	ND	ND	ND	ND	ND	ND	306
ribulose	ND	ND	ND	ND	ND	260	ND
ribulose-5-phosphate	ND	ND	ND	ND	312	ND	ND
salicylaldehyde	ND	424	ND	ND	ND	ND	230

salicylic acid	ND	ND	ND	ND	ND	1079	ND
sarcosine	ND	ND	ND	ND	268	ND	ND
serine	ND	149	ND	ND	ND	1080	ND
shikimic acid	ND	248	ND	ND	ND	1081	ND
sophorose	ND	ND	ND	ND	55	198	930
sorbitol	ND	358	ND	ND	29	1083	931
spermidine	ND	255	ND	ND	ND	ND	ND
sphingomyelin d33:1	ND	ND	104	ND	ND	ND	ND
sphingomyelin d34:0	ND	ND	270	ND	ND	ND	ND
sphingomyelin d34:1	ND	ND	111	ND	ND	ND	ND
sphingomyelin d34:2	ND	ND	327	ND	ND	ND	ND
sphingomyelin d36:1	ND	ND	213	ND	ND	ND	ND
sphingomyelin d36:2	ND	ND	352	ND	ND	ND	ND
sphingomyelin d37:1	ND	ND	112	ND	ND	ND	ND
sphingomyelin d38:2	ND	ND	171	ND	ND	ND	ND
sphingomyelin d40:2	ND	ND	49	ND	ND	ND	ND
sphingomyelin d41:2	ND	ND	27	ND	ND	ND	ND
sphingomyelin d42:3	ND	ND	356	ND	ND	ND	ND
stearic acid	ND	ND	ND	ND	ND	1084	ND
suberic acid	ND	ND	ND	ND	ND	1085	ND
succinate semialdehyde	ND	185	ND	ND	326	1086	886
succinic acid	ND	405	ND	ND	265	1072	658
sucrose	ND	303	ND	ND	156	ND	ND
sulfuric acid	ND	ND	ND	ND	ND	581	ND
taurine	ND	ND	ND	ND	ND	ND	929
terephthalic acid	ND	ND	ND	ND	ND	364	ND
tetracosane	ND	ND	ND	ND	ND	ND	147
tetracosanol	ND	ND	ND	ND	ND	ND	309
threitol	ND	ND	ND	ND	158	ND	ND
threonic acid	ND	24	ND	ND	333	58	927
threonine	ND	517	ND	ND	ND	551	ND
thymidine	ND	ND	ND	ND	ND	553	ND
thymine	ND	ND	ND	ND	ND	ND	264
trans-4-hydroxyproline	ND	233	ND	ND	304	555	925
triacylglycerol 34:0	ND	ND	10	ND	ND	ND	ND
triacylglycerol 38:0	ND	ND	147	ND	ND	ND	ND
triacylglycerol 40:0	ND	ND	315	ND	ND	ND	ND
triacylglycerol 40:1	ND	ND	305	ND	ND	ND	ND
triacylglycerol 42:1	ND	ND	19	ND	ND	ND	ND
triacylglycerol 42:2	ND	ND	214	ND	ND	ND	ND
triacylglycerol 42:3	ND	ND	313	ND	ND	ND	ND
triacylglycerol 43:3	ND	ND	275	ND	ND	ND	ND
triacylglycerol 44:1	ND	ND	254	ND	ND	ND	ND

triacylglycerol 44:2	ND	ND	247	ND	ND	ND	ND
triacylglycerol 44:3	ND	ND	349	ND	ND	ND	ND
triacylglycerol 46:1	ND	ND	155	ND	ND	ND	ND
triacylglycerol 46:2	ND	ND	9	ND	ND	ND	ND
triacylglycerol 46:3	ND	ND	56	ND	ND	ND	ND
triacylglycerol 46:4	ND	ND	318	ND	ND	ND	ND
triacylglycerol 46:5	ND	ND	328	ND	ND	ND	ND
triacylglycerol 48:0	ND	ND	98	ND	ND	ND	ND
triacylglycerol 48:1	ND	ND	174	ND	ND	ND	ND
triacylglycerol 48:3	ND	ND	18	ND	ND	ND	ND
triacylglycerol 48:4	ND	ND	157	ND	ND	ND	ND
triacylglycerol 49:0	ND	ND	216	ND	ND	ND	ND
triacylglycerol 49:2	ND	ND	245	ND	ND	ND	ND
triacylglycerol 49:4	ND	ND	294	ND	ND	ND	ND
triacylglycerol 50:2	ND	ND	239	ND	ND	ND	ND
triacylglycerol 50:3	ND	ND	24	ND	ND	ND	ND
triacylglycerol 50:4	ND	ND	31	ND	ND	ND	ND
triacylglycerol 50:5	ND	ND	296	ND	ND	ND	ND
triacylglycerol 50:6	ND	ND	118	ND	ND	ND	ND
triacylglycerol 51:1	ND	ND	209	ND	ND	ND	ND
triacylglycerol 51:2	ND	ND	57	ND	ND	ND	ND
triacylglycerol 51:3	ND	ND	54	ND	ND	ND	ND
triacylglycerol 51:4	ND	ND	96	ND	ND	ND	ND
triacylglycerol 51:5	ND	ND	193	ND	ND	ND	ND
triacylglycerol 52:0	ND	ND	145	ND	ND	ND	ND
triacylglycerol 52:1	ND	ND	220	ND	ND	ND	ND
triacylglycerol 52:2	ND	ND	8	ND	ND	ND	ND
triacylglycerol 52:3	ND	ND	116	ND	ND	ND	ND
triacylglycerol 52:4	ND	ND	180	ND	ND	ND	ND
triacylglycerol 52:5	ND	ND	293	ND	ND	ND	ND
triacylglycerol 52:7	ND	ND	332	ND	ND	ND	ND
triacylglycerol 53:1	ND	ND	312	ND	ND	ND	ND
triacylglycerol 53:2	ND	ND	185	ND	ND	ND	ND
triacylglycerol 53:3	ND	ND	97	ND	ND	ND	ND
triacylglycerol 53:5	ND	ND	60	ND	ND	ND	ND
triacylglycerol 54:1	ND	ND	235	ND	ND	ND	ND
triacylglycerol 54:2	ND	ND	250	ND	ND	ND	ND
triacylglycerol 54:3	ND	ND	69	ND	ND	ND	ND
triacylglycerol 54:6	ND	ND	178	ND	ND	ND	ND
triacylglycerol 54:7	ND	ND	287	ND	ND	ND	ND
triacylglycerol 54:9	ND	ND	134	ND	ND	ND	ND
triacylglycerol 55:1	ND	ND	342	ND	ND	ND	ND
triacylglycerol 55:2	ND	ND	143	ND	ND	ND	ND

triacylglycerol 55:3	ND	ND	195	ND	ND	ND	ND
triacylglycerol 56:1	ND	ND	264	ND	ND	ND	ND
triacylglycerol 56:10	ND	ND	236	ND	ND	ND	ND
triacylglycerol 56:2	ND	ND	346	ND	ND	ND	ND
triacylglycerol 56:3	ND	ND	330	ND	ND	ND	ND
triacylglycerol 56:5	ND	ND	26	ND	ND	ND	ND
triacylglycerol 56:6	ND	ND	194	ND	ND	ND	ND
triacylglycerol 56:7	ND	ND	51	ND	ND	ND	ND
triacylglycerol 56:8	ND	ND	58	ND	ND	ND	ND
triacylglycerol 56:9	ND	ND	347	ND	ND	ND	ND
triacylglycerol 57:2	ND	ND	240	ND	ND	ND	ND
triacylglycerol 57:4	ND	ND	282	ND	ND	ND	ND
triacylglycerol 58:10	ND	ND	78	ND	ND	ND	ND
triacylglycerol 58:3	ND	ND	234	ND	ND	ND	ND
triacylglycerol 58:4	ND	ND	204	ND	ND	ND	ND
triacylglycerol 58:5	ND	ND	298	ND	ND	ND	ND
triacylglycerol 58:6	ND	ND	184	ND	ND	ND	ND
triacylglycerol 58:9	ND	ND	177	ND	ND	ND	ND
triacylglycerol 59:3	ND	ND	258	ND	ND	ND	ND
triacylglycerol 60:11	ND	ND	191	ND	ND	ND	ND
triacylglycerol 60:4	ND	ND	154	ND	ND	ND	ND
triacylglycerol 60:5	ND	ND	55	ND	ND	ND	ND
triacylglycerol 60:6	ND	ND	12	ND	ND	ND	ND
triacylglycerol 68:4;O2	ND	ND	126	ND	ND	ND	ND
triacylglycerol 70:3;O2	ND	ND	351	ND	ND	ND	ND
triacylglycerol 70:4;O2	ND	ND	357	ND	ND	ND	ND
triacylglycerol 70:5;O2	ND	ND	226	ND	ND	ND	ND
triacylglycerol 72:5;O2	ND	ND	362	ND	ND	ND	ND
triacylglycerol 72:6;O2	ND	ND	326	ND	ND	ND	ND
triacylglycerol 72:7;O2	ND	ND	335	ND	ND	ND	ND
triacylglycerol O-38:2	ND	ND	146	ND	ND	ND	ND
triacylglycerol O-50:1	ND	ND	271	ND	ND	ND	ND
triacylglycerol O-50:2	ND	ND	135	ND	ND	ND	ND
triacylglycerol O-52:0	ND	ND	173	ND	ND	ND	ND
triacylglycerol O-52:1	ND	ND	337	ND	ND	ND	ND
triacylglycerol O-52:2	ND	ND	117	ND	ND	ND	ND
triacylglycerol O-52:3	ND	ND	16	ND	ND	ND	ND
triacylglycerol O-54:1	ND	ND	127	ND	ND	ND	ND
triacylglycerol O-54:2	ND	ND	289	ND	ND	ND	ND
triacylglycerol O-54:3	ND	ND	269	ND	ND	ND	ND
triacylglycerol O-58:2	ND	ND	304	ND	ND	ND	ND
tryptophan	ND	ND	ND	ND	183	556	924
tyrosine	ND	69	ND	ND	404	561	ND

uracil	ND	435	ND	ND	ND	259	ND
urea	ND	212	ND	ND	420	ND	298
uric acid	ND	ND	ND	ND	149	562	181
uridine	ND	ND	ND	ND	ND	565	922
valine	ND	ND	ND	ND	379	566	ND
xanthine	ND	198	ND	ND	ND	234	ND
xanthosine	ND	ND	ND	ND	ND	ND	921
xylitol	ND	417	ND	ND	401	188	920
xylonic acid	ND	ND	ND	ND	291	ND	ND
xylonic acid isomer	ND	336	ND	ND	ND	ND	ND
xylonolactone	ND	ND	ND	ND	ND	572	919
xylose	ND	304	ND	ND	203	ND	252
xylulose	ND	418	ND	ND	580	550	915
