# Supplementary material for: Multi-Tissue Metabolomic Signatures of Five Longevity Interventions Converge on Ergothioneine and Lipid Remodeling in Male UM-HET3 Mice
Source: bioRxiv. 2026 Jul 2:2026.06.24.734388. Preprint. [Version 2] doi: 10.64898/2026.06.24.734388 (PMC13345011; doi:10.64898/2026.06.24.734388)
Supplement: Supplement 2 — Supplementary table 1. Number of metabolites (features) in each tissue dataset used in this study. Tissues: brain, perigonadal fat, inguinal fat, kidney, liver, muscle, plasma. Supplemental Table 2. Category over-representation by tissue at 90% feature explainability power for each individual tissue. The “Dataset Count” is the number of times the metabolite category is found in the whole dataset for a particular tissue. The “Observed Count” is the number of times that the metabolite category is observed in the top 90% of predictive features, as given in the “Metabolites needed to explain 90% Prediction column”. The statistical test performed on the data was a binomial test for skew; raw and Benjamini-Hochberg adjusted (“FDR-BH”) p-values are reported. Supplementary table 3. Number of iterations until convergence for the XGBoost algorithm applied to each tissue type. Tissues: brain, perigonadal fat, inguinal fat, kidney, liver, muscle, plasma. Supplementary table 4. Ratio of total phosphatidylcholine (PC) in pooled male longevity interventions versus control, by tissue. For each tissue, metabolite columns classified as phosphatidylcholine (PC) were identified (LysoPC and sphingomyelin excluded). Linear-scale intensities (log2_intensity) were summed over all PC species and over samples in control or in pooled male treatment groups (rapamycin, acarbose, canagliflozin, 17α-estradiol, calorie restriction). The table reports the ratio of total treatment PC to total control PC and its log2 value (log2_PC_ratio). “n PC species” is the number of PC metabolites detected per tissue. Supplementary Table 5. Cross-tissue metabolite feature importance rankings from 1,000 XGBoost gain iterations. Each row represents a unique metabolite detected across the seven tissue panels (brain, gonadal fat, inguinal fat, kidney, liver, muscle, and plasma). Columns display the final rank of each metabolite within each tissue, determined by median XGBoost gain importance averaged over 1,000 iter [file media-2.pdf]

Supplemental Figures

Supplementary table 1. Number of metabolites (features) in each tissue dataset used in this study. Tissues: brain, perigonadal fat, inguinal fat, kidney, liver, muscle, plasma.

Tissue	Number of Features
Brain	572
Perigonadal Fat	395
Inguinal Fat	755
Kidney	946
Liver	580
Muscle	1,087
Plasma	1,051

Supplemental Table 2. Category over-representation by tissue at 90% feature explainability power for each individual tissue. The “Dataset Count” is the number of times the metabolite category is found in the whole dataset for a particular tissue. The “Observed Count” is the number of times that the metabolite category is observed in the top 90% of predictive features, as given in the “Metabolites needed to explain 90% Prediction column”. The statistical test performed on the data was a binomial test for skew; raw and Benjamini-Hochberg adjusted (“FDR-BH”) p-values are reported.

Tissue	Metabolites needed to explain 90% Prediction	Metabolite Category	Observed Count	Dataset Count	Binomial Skew P Value	FDR BH
Brain	64	Biogenic amine	5	12	1.1E-02	1.2E-01
Perigonadal Fat	58	FA (fatty acid)	17	55	2.0E-03	9.9E-03*
Perigonadal Fat	58	DG (diacylglycerol)	7	22	4.2E-02	1.0E-01
Kidney	11	PC (phosphatidylcholine)	5	105	4.4E-03	8.7E-03*
Liver	9	SM (sphingomyelin)	2	23	4.7E-02	1.2E-01
Muscle	96	DG (diacylglycerol)	9	42	1.2E-02	1.3E-01
Muscle	96	Ceramide / Glucosylceramide	5	21	3.9E-02	2.1E-01
Plasma	52	TAG (triacylglycerol)	19	170	4.1E-04	2.4E-03*
Plasma	52	DG (diacylglycerol)	6	23	9.4E-04	2.8E-03*

Supplementary table 3. Number of iterations until convergence for the XGBoost algorithm applied to each tissue type. Tissues: brain, perigonadal fat, inguinal fat, kidney, liver, muscle, plasma.

Tissue	Convergence Iterations XGBoost Gain
Brain	300
Perigonadal Fat	400
Inguinal Fat	400
Kidney	500
Liver	300
Muscle	500
Plasma	400

Supplementary table 4. Ratio of total phosphatidylcholine (PC) in pooled male longevity interventions versus control, by tissue. For each tissue, metabolite columns classified as phosphatidylcholine (PC) were identified (LysoPC and sphingomyelin excluded). Linear-scale intensities (log2_intensity) were summed over all PC species and over samples in control or in pooled male treatment groups (rapamycin, acarbose, canagliflozin, 17α-estradiol, calorie restriction). The table reports the ratio of total treatment PC to total control PC and its $\log_2$ value (log2_PC_ratio). "n PC species" is the number of PC metabolites detected per tissue.

Tissue	n PC species	PC ratio	log2 PC ratio
Brain	49	2.90	1.54
Perigonadal Fat	23	3.72	1.90
Inguinal Fat	40	4.07	2.02
Kidney	105	2.80	1.49
Liver	72	2.87	1.52
Muscle	138	3.39	1.76
Plasma	117	2.46	1.30

Supplementary Table 5. Cross-tissue metabolite feature importance rankings from 1,000 XGBoost gain iterations. Attached as a separate file.

Supplementary Table 6 Convergence of XGBoost Gain feature importance rankings across tissues. Convergence iteration indicates the first step at which Spearman rank correlation (ρ_T) >= 0.98 and total variation distance (D_T) <= 0.02 were met for two successive iteration steps, evaluated on a fixed top-feature set defined at 1,000 iterations. All tissues achieved near-perfect rank stability ($\rho_T > 0.99$, $D_T < 0.01$) by 1,000 iterations.

Tissue	N Features	Convergence Iteration	Final ρT (1,000 iter.)	Final DT (1,000 iter.)
Brain	572	300	0.9994	0.0037
Liver	580	300	1.0000	0.0038
Plasma	1,051	400	0.9992	0.0057
Gonadal Fat	395	400	0.9981	0.0054
Inguinal Fat	755	400	0.9964	0.0049
Kidney	946	500	0.9964	0.0078
Muscle	1,087	500	0.9979	0.0076
